# Dreaming during anaesthesia: a scoping review

**DOI:** 10.1016/j.bja.2026.05.002

**Published:** 2026-06-11

**Authors:** Pilleriin Sikka, Sherry Hu, Boris D. Heifets

**Affiliations:** 1 Department of Anesthesiology, Perioperative and Pain Medicine, School of Medicine, Stanford University, Stanford, CA, USA; 2 Department of Psychology, Stanford University, Stanford, CA, USA; 3 Department of Cognitive Neuroscience and Philosophy, School of Bioscience, University of Skövde, Sweden; 4 Department of Psychology, University of Turku, Finland; 5 Department of Psychiatry and Behavioral Sciences, School of Medicine, Stanford University, Stanford, CA, USA

**Keywords:** anaesthesia dreaming, disconnected consciousness, dreams, intraoperative awareness, ketamine, neural correlates, phenomenology, propofol

## Abstract

**Background:**

Dreaming during anaesthesia has been documented for over a century, yet it remains poorly understood. This scoping review synthesises evidence on the frequency, phenomenology, predictors, outcomes, and neural correlates of anaesthesia dreaming.

**Methods:**

We systematically searched multiple databases for studies examining anaesthesia dreaming, including clinical trials, observational studies, and experimental investigations in adult and paediatric patients, as well as in healthy volunteers.

**Results:**

We identified 157 studies (1921–2024) involving 87 866 participants. Dream recall varied widely, with higher rates in experimental settings (often 40%–80%) compared with large-scale clinical trials (typically 3%–8%), likely reflecting methodological differences. Ketamine (clinical: 0%–95%; experimental: 44%–100%) and propofol (clinical: 0%–64%; experimental: 0%–77%) were most consistently associated with recall. Dreams were predominantly pleasant, featuring everyday autobiographical content. Higher home dream recall frequency, preoperative suggestion or priming, and immediate post-emergence assessment were associated with higher recall. Evidence regarding clinical outcomes was limited, but unpleasant dreams were linked to lower willingness to undergo the same anaesthetic again. Dream recall was not associated with intraoperative awareness, challenging interpretations of dreams as markers of inadequate anaesthetic depth. Emerging neural evidence suggests that dreaming exhibits signatures distinct from unconsciousness and connected consciousness.

**Conclusions:**

Anaesthesia dreaming is common, predominantly positive, and pharmacologically specific. Major gaps in knowledge include inconsistent definitions, inadequate assessment methods, limited mechanistic understanding, sparse paediatric research, and minimal outcome data. Standardised approaches informed by consciousness science and sleep research are needed. The positive nature and suggestibility of anaesthesia dreams suggest potential therapeutic applications warranting investigation.

**Editor’s key points**

• Dreaming has fascinated anaesthesia researchers for more than a century. This scoping review synthesised this wealth of existing evidence and reviewed the neural correlates and adverse events associated with anaesthesia dreaming.
• Between 1921 and 2024, 157 studies involving 87 866 participants were published. Dream recall was more commonly reported in experimental settings than clinical ones and with ketamine and propofol than other agents. Dreams were mostly pleasant and were not associated with adverse postoperative outcomes, including intraoperative awareness. Emerging neural evidence suggested that dreaming is distinct from unconsciousness and connected consciousness.
• Future research should enhance our understanding of the mechanisms of anaesthesia, dreams and other issues related to connection and consciousness during anaesthesia.

Even when people appear fully unconscious under anaesthesia, they may experience vivid, internally generated experiences. These experiences, referred to as anaesthesia dreaming, challenge the long-held assumption that behavioural unresponsiveness implies unconsciousness.^1^^,^^2^ Instead, they suggest that consciousness can persist in altered or covert forms, even under pharmacologically induced disconnection from the environment.^3^

Anaesthesia dreaming is defined as subjective experiences (excluding intraoperative awareness) occurring between the onset of anaesthesia and the return of environmental awareness.^4^^,^^5^ It is considered a form of disconnected consciousness, in which internally generated experiences occur without sensory input or behavioural responsiveness.^2^^,^^6^ In contrast, connected consciousness involves preserved awareness of the external environment or internal bodily states, an experience which in the context of surgery is associated with considerable distress.^7^^,^^8^ Although intraoperative awareness has long been a focus of concern in anaesthesiology research and practice,^9^ dreaming under anaesthesia has remained comparatively overlooked.

Understanding anaesthesia dreaming is important for both clinical and theoretical reasons. Clinically, it has implications for monitoring anaesthetic depth, enhancing perioperative communication, and improving patient care.^1^ Theoretically, it offers a unique window into the nature and neural mechanisms of consciousness, highlighting the brain’s capacity to generate rich subjective experiences in the absence of external input.^3^^,^^10^^,^^11^

Despite increasing interest, the scientific literature on anaesthesia dreaming remains fragmented. Studies differ widely in terminology, methodology, anaesthetic techniques, and findings regarding frequency, predictors, clinical outcomes, and neural correlates are inconsistent. To date, no systematic or scoping reviews have synthesised this growing but disparate body of work.

We aimed to review and synthesise existing evidence on the frequency, content, predictors, and outcomes of anaesthesia dreaming in both surgical and experimental settings. We also reviewed the neural correlates and adverse events associated with anaesthesia dreaming. Through systematic exploration, we sought to clarify emerging patterns, identify gaps in current knowledge, and propose future research directions.

## Methods

We followed the Preferred Reporting Items for Systematic reviews and Meta-Analyses Extension for Scoping Reviews^12^ (see Supplementary File S1). Institutional Review Board approval was not required for the secondary analysis of published data. We pre-registered our search process (https://osf.io/w4dhz/).

### Search strategy

After consultation with a medical librarian, Medline/PubMED, EMBASE, and PsycINFO were searched for relevant articles published as far back as the databases allowed until February 26, 2024 (the date when the search was conducted). Additional articles were identified by searching references in relevant articles. Full search strings are provided in Supplementary File S2.

### Inclusion and exclusion criteria

Articles were included, if they met the following criteria:

(a) full-text articles published in English in peer-reviewed journals;
(b) employed an empirical study design (including observational, experimental, case-control studies, case series, or case reports);
(c) involved any surgical, medical, or procedural context, including non-operative procedures;
(d) included human participants of any age (adults, adolescents, or children), either patients or healthy volunteers;

Articles were excluded, if they were:

(a) animal studies;
(b) non-empirical papers (e.g. opinion papers, editorials, reviews/meta-analyses, commentaries).

Because we defined anaesthesia dreaming as a form of disconnected consciousness, we included only studies where participants experienced environmental disconnection during general anaesthesia or deep sedation, evidenced by one or more of the following: (1) unresponsiveness to verbal or tactile stimuli during at least part of the anaesthesia (e.g. Ramsay Sedation Scale score ≥5, Observer’s Assessment of Alertness/Sedation score ≤2); (2) use of anaesthetic agents/doses known to reliably produce such states; (3) use of airway management/controlled ventilation; (4) explicit description of deep sedation or general anaesthesia achieved; or (5) for dissociative anaesthetics (e.g. ketamine), dosing combined with study description indicating deep sedation or general anaesthesia regardless of behavioural responsiveness. We focused on actual sedation depth achieved rather than intended sedation depth.

We excluded studies involving conscious or moderate sedation, regional anaesthesia without co-administered sedatives inducing unresponsiveness, and sub-anaesthetic dosing of anaesthetic agents (unless clear evidence that the dosing led to the loss of responsiveness or disconnection from the external environment was provided).

### Study selection and search results

**Fig 1:**
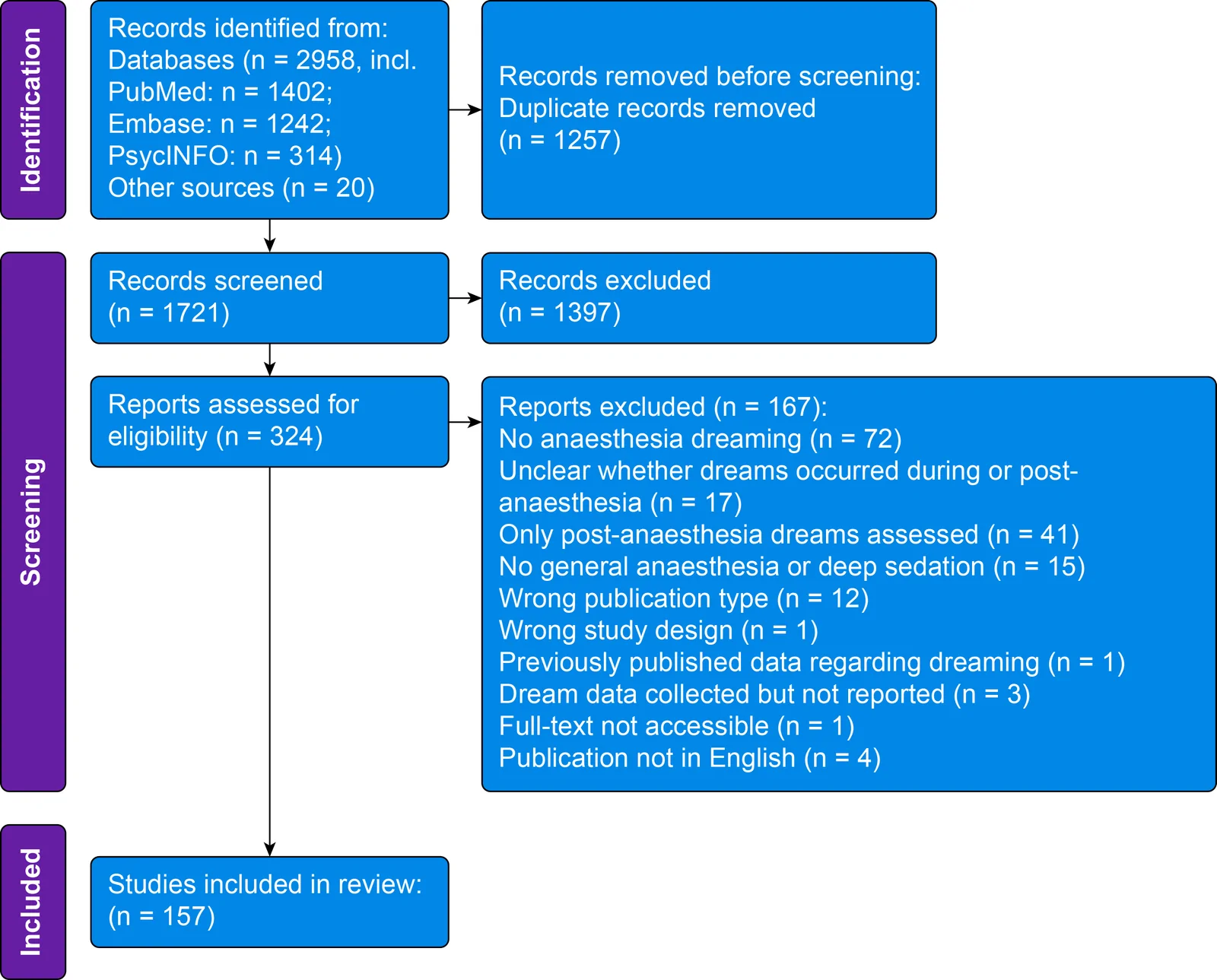
PRISMA flow diagram depicting the search, selection, and screening process. Based on the PRISMA flow diagram template.^14^ PRISMA, Preferred Reporting Items for Systematic Reviews and Meta-Analyses.

The first author (PS) conducted database searches using the predefined search strings. The search resulted in 2958 articles that were imported into Rayyan, a web-based application for systematic reviews.^13^ An additional 20 articles were identified in relevant review papers and manually added, resulting in a total of 2978 articles. After duplicate removal, 1721 articles remained (see Fig. 1).

In the first stage, two authors (PS and SH) independently screened titles and abstracts using Rayyan’s blind mode. After decisions were made, the blind mode was disabled, and inter-rater reliability was calculated. Following initial screening, the authors agreed on 1580 articles (1293 excluded, 287 included) and disagreed on 141 (8.2%). After discussion of the disagreements, consensus was reached, and 37 articles were included and 104 excluded, resulting in 324 included articles.

In the second stage, the two authors independently screened the full texts. Of the 324 articles, they agreed on 254 articles (149 included, 105 excluded) and disagreed on 70 (21.6%) articles. After discussion, the authors decided to include eight articles and exclude 62 articles. Thus, of the 324 articles, 167 were excluded, and 157 articles remained for the final review.

### Data extraction

The two authors extracted data from the final selection of articles using a standardised tabulation form, with discrepancies resolved through discussion. Extracted variables included study characteristics (design, setting, sample size, definitions of dreaming and awareness), participant characteristics (demographics, baseline traits and states, clinical characteristics, home dream recall frequency), anaesthetic and procedural variables (agents, doses, duration, processed EEG monitoring, use of instructions or suggestions), assessment timing and methods, emergence characteristics, dream recall frequency and content, frequency of awareness, postoperative outcomes, adverse events related to dreaming, and neural correlates of anaesthesia dreaming.

## Results

### Overview of included studies

**Fig 2:**
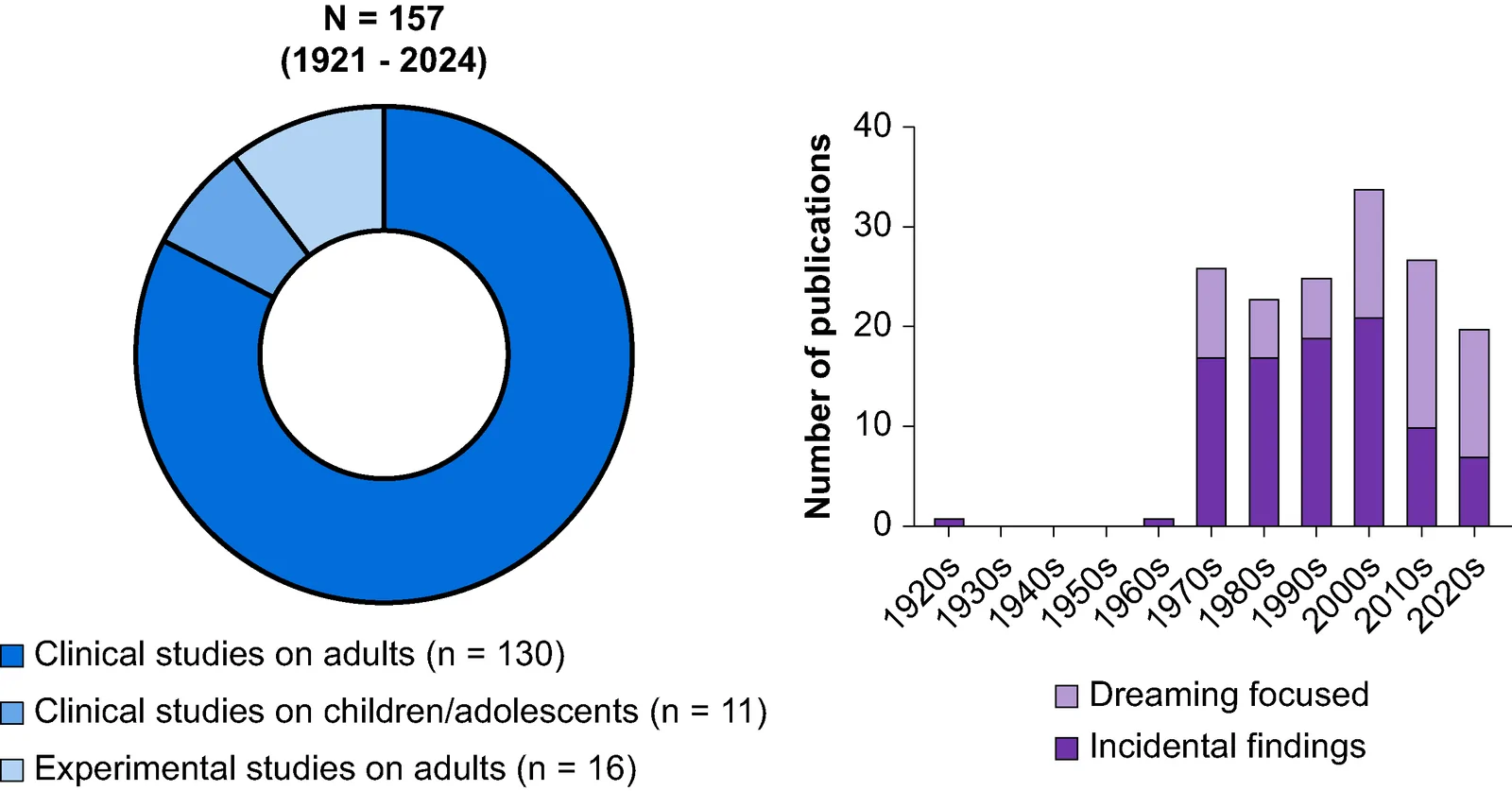
Number of publications on anaesthesia-related dreaming by decade. In the bar graph, bars represent the total number of studies per decade, segmented into those that focused explicitly on dreaming and those that reported dreaming-related findings incidentally.

Of the 157 studies included in this review, published between 1921 and 2024, 130 (82.8%) were clinical studies involving adult patients,‡ 11 (7.0%) clinical studies focused on children and/or adolescents, and 16 (10.2%) were experimental studies with healthy adult volunteers. Across all categories, there was a clear temporal shift: in earlier decades (1920s–1990s), dream-related findings were typically reported incidentally, whereas from the 2000s onward, an increasing number of studies examined dreaming as a primary outcome (see Fig. 2).

#### Definition of dreaming

Only 13 of the 141 (9.2%) clinical studies provided an explicit definition of dreaming (see Supplementary File S3). Nearly all were based on, or closely resembled, the widely adopted phrasing from Brice and colleagues^4^ (p. 536, emphasis added): *‘…any experience (excluding awareness) which a patient was able to recall and which he or she thought occurred between the induction of anaesthesia and the first moment of consciousness after anaesthesia’*. In these studies, dreaming was typically conceptually distinguished from awareness, the latter referring to the conscious recall of intraoperative events.

By contrast, nearly all experimental studies (12/16; 75%) provided an explicit and theoretically grounded definition of dreaming, typically framing it as a form of disconnected consciousness—an internally generated, stimulus-independent subjective experience occurring during unresponsiveness.^15^, ^16^, ^17^ This state was clearly contrasted with connected consciousness, defined as awareness of the external environment during the anaesthetised state.^18^^,^^19^ This conceptual distinction has become central in recent experimental anaesthesia and consciousness research.

Nevertheless, the distinction between dreaming and awareness was not always clear-cut. Conceptually, in some studies,^20^ dreaming was classified as a type of awareness, whereas in others,^21^ the definition suggested overlap between internally and externally related experiences. Empirically, some studies reported dreams incorporating elements from the surgical or research environment,^4^^,^^22^, ^23^, ^24^ raising questions about whether such experiences reflected pre-anaesthesia memories or external stimulus processing during anaesthesia. Studies differed in their approach to classifying such ambiguous reports. Some employed mutually exclusive categories, separating dreaming from definite and possible awareness.^25^, ^26^, ^27^, ^28^ Others allowed overlapping classifications,^29^ whereas some considered dreams incorporating contemporaneous stimuli as ’near-miss awareness’.^23^ In two experimental studies,^6^^,^^24^ dreaming (a purely internally generated experience) was distinguished from memory incorporation (experiences containing research setting elements encountered before anaesthesia) and awareness, finding that dream-like content and memory incorporation cooccurred in over half of anaesthesia reports. This methodological heterogeneity reflects differing approaches to handling reports that contain both internally generated and externally referenced elements.

#### Clinical studies in adults

The 130 adult clinical studies, with a total number of 84 945 participants, represent over a century of enquiry into dreaming under anaesthesia, from early case-based descriptions^30^ to large-scale studies conducted in recent years^28^^,^^31^ (see Table S1 in Supplementary File S4).

Study designs included RCTs (51.5%; 26 double-blind), prospective observational studies (38.5%), and other studies (retrospective studies, case studies, and quasi-experimental studies; 10%). Sample sizes ranged from single-case reports ^30^^,^^32^ to large epidemiological studies, the largest involving 19 575 participants,^25^ 11 101 participants,^33^ and 6991 participants.^34^ More than 10 studies enrolled over 1000 participants, including three double-blind RCTs.^27^^,^^35^^,^^36^

Most studies involved adults aged 20–60 yr. Sex representation was skewed, with 40.0% of studies including exclusively female participants, largely because of the inclusion of obstetric and gynaecological procedures. Mixed-sex samples typically had a female majority.^25^^,^^27^^,^^29^^,^^34^

Anaesthetic techniques reflected historical and regional practices. Earlier studies commonly used thiopentone for induction (35.4%), whereas propofol dominated in later studies (47.7%). Ketamine was used in 30.0% of studies, etomidate in 6.9% of studies and dexmedetomidine was used in four studies (3.1%).^37^, ^38^, ^39^, ^40^ A few older studies used thiamylal (*n*=2),^41^^,^^42^ methohexitone (*n*=4),^43^, ^44^, ^45^, ^46^ ether (*n*=1),^30^ and etoxadrol (*n*=1).^47^ Maintenance typically involved volatile anaesthetics (halothane, isoflurane, sevoflurane, desflurane, or methoxyflurane) often combined with nitrous oxide. Opioids (especially fentanyl, remifentanil, and alfentanil), benzodiazepines (especially midazolam and diazepam), and anticholinergics (atropine and scopolamine) were frequently used as premedication or adjuncts.

Processed EEG monitoring (most commonly the bispectral index [BIS]) was employed in 24.6% of studies. One study used SedLine,^32^ one used EEG monitoring without a processed index,^46^ and one used auditory evoked potentials.^48^

Dream recall and content were assessed using structured instruments in 43.0% of studies. The modified Brice questionnaire^4^ was the most common (33.8%), whereas 20.0% reported no information on assessment methods. Interview timing ranged from immediately post-emergence (21.5%) to beyond 24 h post-anaesthesia (23.8%). One third of studies (33.1%) included multiple follow-up interviews. In several studies (17.7%), interview times were either not reported or were only described in broad terms (e.g. ’postoperatively’).

#### Clinical studies on children, adolescents, or both

The 11 paediatric studies included 2301 participants aged 2 months to 17 yr (see Table S1 in Supplementary File S4). Most were prospective observational studies (*n*=7), with two RCTs,^49^^,^^50^ one retrospective/prospective observational study,^51^ and one case study.^52^

Paediatric studies showed a slight male predominance, with female representation ranging from 28.7% to 44.5%. The case study^52^ focused on a single female patient, whereas two studies did not report sex distribution.^53^^,^^54^

Ketamine was the most frequently used agent (7/11 studies; 64%), either as a sole agent^49^, ^50^, ^51^^,^^53^^,^^54^ or in combination with other agents.^52^^,^^55^ Thiopentone^56^^,^^57^ and volatile anaesthetics were also common, particularly in earlier studies. Propofol was administered to a subset of patients in only one study^26^ and no studies used dexmedetomidine. Maintenance anaesthetics included nitrous oxide/oxygen mixtures and, in some ketamine studies, continuous i.v. ketamine administration.^49^^,^^51^^,^^53^ In one study, a fixed ratio of ketamine plus propofol (‘ketofol’) was administered for both induction and maintenance.^58^

None of the paediatric studies reported use of processed EEG monitoring. Assessment of dreaming and awareness relied predominantly on structured interviews, either adapted versions of the modified Brice questionnaire or other interviews developed for the specific study. One study relied on nurse observations^50^ and three provided no information on assessment methods.^52^^,^^53^^,^^58^ Interview timing varied considerably, ranging from the day of surgery^26^^,^^49^^,^^51^^,^^57^ to 30 days post-anaesthesia.^26^^,^^55^ In three studies, the timing was unclear^54^^,^^56^^,^^58^ or not reported at all.^50^^,^^52^^,^^53^

#### Experimental studies

The 16 experimental studies comprised 620 healthy adults, primarily aged between 18 and 40 yr (see Table S1 in Supplementary File S4). However, overlapping datasets reduced the number of unique samples to 10 (*n*=356).

These studies employed a range of designs, including RCTs, within-subject studies, and between-subject studies with within-condition comparisons.

Most studies recruited medically healthy participants. The majority of studies included exclusively male participants,^6^^,^^16^^,^^17^^,^^59^ often because of concurrent positron emission tomography (PET) imaging and radiation exposure constraints. Others enrolled mixed-sex samples,^60^, ^61^, ^62^ whereas six studies did not report the sex distribution in their sample.^18^^,^^19^^,^^63^, ^64^, ^65^, ^66^

Dexmedetomidine and propofol were the most frequently studied agents, typically administered through target-controlled infusion, and often compared with other anaesthetics like ketamine (with two studies using S-ketamine^6^^,^^59^), sevoflurane, and xenon. A smaller subset of studies focused on a single anaesthetic agent, such as etoxadrol (CL-1848C),^67^ ketamine,^62^ midazolam-ketamine admixture,^64^ or dexmedetomidine alone.^65^^,^^66^ Most studies employed stepwise infusion protocols to systematically induce and reverse loss of responsiveness.

The EEG was recorded in all but one study,^64^ generally to analyse brain dynamics under anaesthesia rather than monitor anaesthetic depth. Only one study used BIS to titrate anaesthetic depth and track transitions into and out of unresponsiveness.^15^ Several studies incorporated multimodal neuroimaging approaches (e.g. PET-EEG, EEG-functional MRI) to explore the neural correlates of conscious experience during anaesthesia.^17^^,^^62^ Others combined EEG with transcranial magnetic stimulation (TMS) to assess cortical responsiveness and information integration under anaesthetic states.^60^

Interviews were typically conducted immediately upon emergence or shortly thereafter, whereas some studies incorporated repeated interviews after each awakening^16^^,^^18^ or verified dream reports after full recovery.^60^ A few studies conducted interviews months after the session,^62^ whereas three did not report interview timing.^64^^,^^66^^,^^67^ Many studies used structured interviews, often based on the modified Brice questionnaire, whereas others employed open-ended phenomenological interviews.^60^

### Frequency of anaesthesia dreaming

#### Clinical studies on adults

In adult clinical studies, reported dream recall varied widely, from 0% (16% of studies) to as high as 94.6%^68^ (see Table S1 in Supplementary File S4). The highest rates were consistently observed with ketamine. In fact, all studies reporting recall rates ≥65% used ketamine^69^, ^70^, ^71^, ^72^, ^73^ or the related dissociative anaesthetic etoxadrol.^47^ Studies using propofol reported dream recall frequencies ranging from 0%^74^, ^75^, ^76^, ^77^, ^78^ to 64%.^79^ Earlier studies using thiopentone for induction followed by volatile maintenance also showed considerable variation, from 0% to 57%.^22^ Dream recall rates in studies using volatile anaesthetics, typically in combination with nitrous oxide, ranged from 0%^80^, ^81^, ^82^ to 60%,^83^ with most values falling between 3% and 8%. The few studies using dexmedetomidine, either alone or in combination with other agents, reported recall rates from 3%^37^ to 41%.^40^

Large-scale studies generally reported lower dream recall frequencies, typically at the lower end of the spectrum: 3%–6%,^25^ 3%,^33^ and 8%.^34^ Similarly, the largest double-blind RCTs reported rates between 3% and 7%.^27^^,^^35^^,^^36^

For comparison, among the 89 studies in this dataset reporting awareness rates, half (51%) reported 0% awareness, and most reported rates between 0% and 2%. The highest awareness rate was 30.8% in a study using ketamine.^72^ The largest studies reported awareness rates of 0.1%–0.4%.^25^^,^^33^^,^^34^

#### Clinical studies on children, adolescents. or both

In paediatric studies, dream recall frequency ranged from 0%^51^ to 50%,^54^ with both extreme values observed in studies using ketamine-based anaesthesia (see Table S1 in Supplementary File S4). However, most studies reported dream recall rates of 5%–20%. No consistent pattern emerged in relation to the type of anaesthetic agent. However, some evidence suggests that premedication with muscle relaxants (tubocurarine) and midazolam is associated with lower dream recall (no tubocurarine: 16.7% *vs* tubocurarine: 2.8%^57^; ketamine: 10.9% *vs* ketamine + midazolam: 4.4%^50^), and that immediate interviews are associated with higher dream recall rates (9.7%^57^; 10.4%^26^) than when interviews are delayed 24 h or more (e.g. 5.9%^55^).

Of the five (46%) studies reporting intraoperative awareness, frequencies ranged from 0% to 2.5%,^55^ with most studies reporting 0%.^56^, ^57^, ^58^

#### Experimental studies

In experimental studies, dream recall ranged from 0%^60^ to 100%,^64^ typically exceeding rates observed in clinical settings (see Table S1 in Supplementary File S4). Reported frequencies varied notably by anaesthetic agent. Ketamine consistently yielded the highest rates, ranging from 44% (S-ketamine^6^) to 100%.^60^^,^^64^ However, high recall under ketamine may coincide with light sedation, as evidenced by high awareness rates (e.g. 25%^6^). Ketamine may also be associated with slower emergence as, in one study, none of the 20 participants could be interviewed post-infusion because of prolonged drug effects.^59^

Dexmedetomidine also showed elevated dream recall, ranging from 35.7%^66^ to 92.5%.^16^ Awareness was reported in 22.6% of participants in the latter study, suggesting that lighter sedation depth may have contributed to high dream recall. Dream recall under propofol varied widely, from 0%^60^ to 77.3%.^24^ In several other studies, rates were around 16%^18^ or 30%^6^^,^^15^ underscoring the role of other methodological factors. Xenon-based anaesthesia showed similarly broad variability, with rates ranging from 0%^60^ to 70%.^15^ Sevoflurane was also associated with variable recall rates, from 11.4%^6^ to 60%.^15^

In experimental studies, awareness ranged from 0% to 44.3%,^18^ with significantly higher rates during ketamine (e.g. 25%^6^) and dexmedetomidine sedation (22.6%^16^).

### Emotional quality and content of anaesthesia dreams

Of the 130 adult clinical studies, 90 (69%) reported data on dream valence and/or content (see Table S1 in Supplementary File S5). Pleasant dreams predominated, with only nine studies reporting a higher proportion of unpleasant dreams (involving ether,^30^ thiopentone with volatile anaesthetics,^84^^,^^85^ ketamine,^43^^,^^72^^,^^86^^,^^87^ propofol,^88^ or volatile anaesthetics^89^).

Dream content typically reflected everyday life content, such as family, friends, work, school, vacationing or travelling, enjoying outdoor or beach settings, and engaging in recreational activities. Surgery- or hospital-related dreams were also common, though they were generally positive in tone. Some studies reported existential, spiritual, or religious themes. When rated using standardised scales, dreams were generally described as vivid, moderately memorable and emotionally intense, meaningful, and not particularly strange.^23^^,^^83^^,^^90^ Sexual content was rare, reported in only two studies,^91^^,^^92^ and even then, only vaguely related to sexual themes.

Studies involving ketamine often described vivid, surreal, and disembodied experiences, such as floating or flying, intense colours and visual distortions, altered perceptions of time and space, out-of-body experiences, and themes of death or unreality. Nightmares were reported in only five studies,^30^^,^^35^^,^^71^^,^^85^^,^^93^ two of which may reflect possible awareness.^30^^,^^35^ Although nightmares occurred across different anaesthetic agents, they appeared more likely when ketamine was used.^71^^,^^93^

Clinical paediatric studies (8/11, or 72.7% reporting content) showed similar patterns: predominantly pleasant dreams featuring family, school, animals, playing sports, fantastical or travel scenarios, food, toys, and unusual sensory experiences. Hospital- and surgery-related themes were also common. Four studies reported nightmares.^26^^,^^50^^,^^52^^,^^55^

Of the eight experimental studies (50%) reporting valence or content, dreams were generally positive or emotionally balanced. Propofol and dexmedetomidine dreams tended to be static, whereas ketamine and sevoflurane dreams were more dynamic.^6^ Experiences were predominantly visual, auditory, and kinaesthetic, with other sensory modalities less frequent, and cognitive processes were common.^16^^,^^24^ Content largely mirrored that of clinical studies, featuring friends, vacationing, recreation, and beach,^15^ whereas studies with etoxadrol or ketamine also included flying and falling sensations, body distortions, unearthly environments, supernatural themes, and death.^62^^,^^67^

### Predictors of anaesthesia dreaming

In total, 71 (54.6%) adult clinical studies examined predictors of dream recall (see Table 1). Most patient characteristics showed mixed patterns: younger age, female sex, better health status (i.e. lower ASA physical status) and higher preoperative state anxiety showed some tendency toward higher dream recall, but results were inconsistent. The most consistent patient-related predictor of anaesthesia dreaming was higher baseline home dream recall frequency.^23^^,^^83^^,^^90^^,^^94^, ^95^, ^96^

**Table 1:** Predictors of anaesthesia dreaming. ↑ = increased dream recall. ↓ = decreased dream recall. BIS, bispectral index; BMI, body mass index; GI, gastrointestinal; n.s., not significant; N_2_O, nitrous oxide; PSI, patient state index. ∗Based on percentages, not on statistical significance testing. ^†^Dexmedetomidine was associated with significantly more content reports (including dreaming, memory incorporation, awareness of the environment) but not significantly more dream reports.

Domain	Predictor	Association with dream recall (and number of studies providing this evidence)	Consistency of findings
**Clinical studies on adults**			
Patient	Younger age	↑ (9)^23^^,^^25^^,^^28^^,^^29^^,^^36^^,^^94^^,^^97^^,^^98^^,^^99^; n.s. (14)^4^^,^^31^^,^^68^^,^^71^^,^^83^^,^^90^^,^^95^^,^^100^^,^^101^^,^^102^, ^103^, ^104^, ^105^, ^106^	Mixed but leaning to n.s.
Patient	Female sex	↑ (4)^29^^,^^36^^,^^99^^,^^107^; ↓ (1)^105^; n.s. (13)^23^^,^^28^^,^^68^^,^^94^^,^^95^^,^^97^^,^^100^^,^^101^^,^^98^^,^^102^, ^103^, ^104^^,^^106^	Mixed but leaning to n.s.
Patient	Lower weight/BMI	↑ (1)^99^; n.s. (11)^23^^,^^28^^,^^31^^,^^83^^,^^90^^,^^96^^,^^97^^,^^100^^,^^104^, ^105^, ^106^	Relatively consistently n.s.
Patient	Lower ASA physical status	↑ (6)^23^^,^^25^^,^^36^^,^^95^^,^^98^^,^^105^; n.s. (4)^28^^,^^94^^,^^100^^,^^104^	Mixed but leaning to ↑
Patient	Education	n.s. (4)^23^^,^^83^^,^^90^^,^^105^	Consistently n.s.
Patient	Higher preoperative state anxiety/symptoms of anxiety	↑ (3)^36^^,^^101^^,^^99^; n.s. (6)^23^^,^^28^^,^^31^^,^^46^^,^^83^^,^^108^	Mixed but leaning to n.s.
Patient	Higher preoperative trait anxiety	n.s. (3)^101^^,^^99^^,^^108^	Consistently n.s.
Patient	Lower preoperative symptoms of depression	↑ (1)^23^; n.s. (4)^28^^,^^31^^,^^36^^,^^83^	Relatively consistently n.s.
Patient	Psychiatric diagnoses	n.s. (1)^100^	Not enough evidence
Patient	Higher home dream recall frequency	↑ (6)^23^^,^^83^^,^^90^^,^^94^, ^95^, ^96^; n.s. (2)^46^^,^^105^	Mixed but leaning to ↑
Patient	Preoperative antidepressants	n.s. (4)^23^^,^^94^^,^^100^^,^^102^	Consistently n.s.
Patient	Preoperative analgesics	n.s. (1)^23^	Not enough evidence
Patient	Other preoperative psychotropic medication	n.s (3)^23^^,^^100^^,^^102^	Consistently n.s.
Patient	Lower preoperative functioning and quality of life	↑ (1)^94^	Not enough evidence
Procedure	Type of procedure	Emergency (*vs* elective): ↑ (1)^109^; ↓ (1)^25^; Abortion (*vs* GI endoscopy): ↑ (1)^90^; n.s. (4)^23^^,^^95^^,^^100^^,^^102^	Mixed
Procedure	Duration of procedure	n.s. (2)^4^^,^^28^	Consistently n.s.
Procedure	Preoperative suggestion/priming	Suggestion of being at a ‘favourite place’ during anaesthesia: ↑ (3)^92^^,^^110^^,^^111^; n.s. (although suggestion associated with ↑ positive dreams and ↓ negative dreams) (1)^71^	Consistently ↑
Anaesthesia	Type of anaesthetic	Propofol (*vs* etomidate/thiopentone/ methohexital-isoflurane/midazolam/volatile anaesthetics): ↑ (7)^23^^,^^28^^,^^46^^,^^91^^,^^97^, ^112^, ^113^; ↓ (1)^83^; n.s. (5)^88^^,^^95^^,^^110^^,^^104^^,^^114^; Propofol + Ketamine (*vs* propofol + fentanyl or thiopentone + fentanyl or methohexitone + fentanyl): ↑ (1)^45^; Etomidate + propofol (*vs* propofol): ↓ (1)^115^; Dexmedetomidine (in addition to propofol *vs* propofol or *vs* midazolam): ↓ (3)^37^, ^38^, ^39^; (in addition to remifentanil *vs* remimazolam + remifentanil): n.s. (1)^40^; Ketamine (*vs* thiopental/volatile anaesthetics/diazepam/fentanyl, droperidol + fentanyl/thiamylal) ↑ (5)^42^,^72^∗^,^^116^∗^,^^117^, ^118^∗; n.s. (2)^102^, ^119^; Thiopentone (*vs* alfathesin) ↑ (1)^120^; Volatile anaesthetics (added to thiopentone *vs* thiopentone): ↓ (1)^22^; Mixed anaesthesia (any i.v. induction drug + O_2_-N_2_O *vs* propofol TIVA or any i.v. induction drug + halogenated agents or miscellaneous techniques): ↓ (1)^107^; Propofol + remifentanil *vs* thiopentone + sevoflurane + fentanyl *vs* propofol + sevoflurane + fentanyl *vs* thiopentone + fentanyl: n.s. (1)^48^; Thiopental with or without N_2_O: n.s. (1)^121^ Opioid bolus *vs* opioid infusion *vs* isoflurane: n.s. (1)^122^	Propofol leaning to ↑ Ketamine leaning to ↑ Dexmedetomidine ↓ Volatile anaesthetics ↓
Anaesthesia	Dose/Concentration of anaesthetic	Higher dose of propofol: ↑ (4)^31^^,^^36^^,^^94^^,^^98^; n.s. (5)^23^^,^^83^^,^^90^^,^^105^^,^^106^; Lower rate of propofol infusion: ↑(1)^103^; n.s. (3)^31^^,^^36^^,^^100^; Higher dose of ketamine: n.s. (3)^42^^,^^96^^,^^123^; Higher concentration of volatile anaesthetics: ↓ (1)^101^; n.s. (2)^23^^,^^95^; Higher concentration of trichloroethylene ↓, but higher concentration of methoxyflurane ↑: (1)^84^∗ Higher dose of remifentanil: ↓ (1)^99^; Higher dose of etomidate: ↓ (1)^115^∗; n.s. (1)^31^; Remimazolam *vs* dexmedetomidine (added to remifentanil): n.s. (1)^40^; Thiopental: n.s (1)^36^	Propofol mixed but leaning to n.s. Ketamine n.s. Volatile anaesthetics n.s.
Anaesthesia	Use or dose of other drugs pre- or during anaesthesia	Sedatives/anxiolytics (Benzodiazepines): ↓ (4)^68^^,^^70^^,^^93^^,^^118^; n.s. (7)^23^^,^^36^^,^^94^^,^^124^^,^^98^^,^^123^^,^^125^; Benzodiazepine antagonists (Flumazenil) ↑ (1)^126^; Opioids (Fentanyl, Morphine) ↓ (5)^22^^,^^79^^,^^94^^,^^127^^,^^128^; n.s. (12)^23^^,^^31^^,^^36^^,^^83^^,^^90^^,^^94^^,^^95^^,^^98^^,^^102^^,^^103^^,^^105^^,^^125^; Opioid antagonists (Naloxone) ↑ (1)^129^; Scopolamine (*vs* atropine) ↓ (1)^77^; Physostigmine n.s. (1)^69^; Muscle relaxants n.s. (2)^36^^,^^103^; Pentobarbitone (*vs* droperidol) n.s.^130^ (1)	Sedatives mixed but leaning to n.s. Opioids mixed but leaning to n.s.
Anaesthesia	Duration (min)	Shorter anaesthesia ↑ (1)^36^; n.s. (11)^23^^,^^28^^,^^83^^,^^90^^,^^94^^,^^95^^,^^100^^,^^101^^,^^102^^,^^104^^,^^105^	Consistently n.s.
Anaesthesia	BIS/PSI during anaesthesia	Lower BIS values ↑ (4)^83^^,^^90^^,^^94^^,^^100^; n.s. (7)^23^^,^^36^^,^^46^^,^^95^^,^^101^^,^^99^^,^^104^	Mixed but leaning to n.s.
Anaesthesia	BIS/PSI upon awakening	Higher BIS ↑ (2)^95^^,^^101^	Consistently ↑
Anaesthesia	Emergence time	Shorter time to eye opening/faster emergence ↑ (4)^23^^,^^94^^,^^101^^,^^99^; n.s. (6)^36^^,^^83^^,^^90^^,^^95^^,^^97^^,^^105^	Mixed but leaning to n.s.
Interview	Timing of interview	Immediately upon extubation (*vs* PACU) ↑ (2)^113^^,^^124^; Number of minutes from end of infusion (all conducted within 8 min) n.s. (3)^83^^,^^90^^,^^105^	Closer to emergence leaning to ↑
**Clinical studies on children and adolescents**			
Patient	Younger age	↑ (1)^26^; n.s. (1)^56^	Mixed
Patient	Female gender	n.s. (2)^26^^,^^56^	Consistently n.s.
Patient	Lower ASA physical status	n.s. (1)^26^	Not enough evidence
Patient	Preoperative states	Calmer during pre-induction: ↑ (1)^56^; State anxiety: n.s. (1)^26^	Mixed
Patient	Preoperative traits	Temperament easy/difficult: n.s. (1)^26^; Trait anxiety: n.s. (1)^26^	Consistently n.s.
Patient	Higher home dream recall frequency	n.s. (2)^56^^,^^57^	Consistently n.s.
Procedure	Type of procedure	n.s. (2)^26^^,^^56^	Consistently n.s.
Procedure	Duration of procedure	n.s. (1)^26^	Not enough evidence
Procedure	Preoperative priming	Role play (*vs* no preparation or reading a book): ↑ (1)^26^	Not enough evidence
Anaesthesia	Type of anaesthetic	Propofol: n.s. (1)^26^; Volatile anaesthetics: n.s. (1)^26^; Thiopentone: n.s. (1)^26^	Not enough evidence
Anaesthesia	Use or dose of other drugs pre- or during anaesthesia	Sedatives/anxiolytics (Benzodiazepines): n.s. (3)^26^^,^^49^^,^^50^	Sedatives consistently n.s.
		‘Ponstan mix’: mefenamic acid, trimeprazine, atropine (*vs* no pre-medication): n.s. (2)^56^^,^^57^; Muscle relaxant suxamethodnium (*vs* atracurium): (1) ↑^56^; Tubocurarine (*vs* no pretreatment): ↓ (1)^57^; Ketamine: n.s. (1)^26^; Opiates: n.s. (1)^26^	Other drugs not enough evidence
Interview	Timing of interview	On the day of anaesthesia (*vs* 3 or more days later): ↑ (1)^26^	Not enough evidence
**Experimental studies**			
Anaesthesia	Type of anaesthetic	Dexmedetomidine (*vs* propofol or sevoflurane or S-ketamine): (3) ↑^15^^,^^16^^,^^18^∗; Ketamine (*vs* propofol or Xenon): ↑ (1)^60^∗; n.s. (dexmedetomidine *vs* propofol *vs* sevoflurane *vs* S-ketamine) (2)^6^^,†,^^24^	Dexmedetomidine leaning to ↑
Anaesthesia	Dose of anaesthetic	Higher concentration of propofol: ↑ (1)^24^; Concentration of dexmedetomidine: n.s. (1)^24^	Not enough evidence
Anaesthesia	BIS/PSI during anaesthesia	Higher BIS ↑ for dexmedetomidine, but lower BIS ↑ for propofol∗ (1)^15^	Not enough evidence

Procedure-related factors were similarly variable. The type of procedure showed no clear pattern, whereas duration of surgery was consistently unassociated with dream recall. In contrast, preoperative suggestion or priming (e.g. imagining being at a ‘favourite place’ before induction) was consistently associated with increased dream recall across the studies that assessed it.^92^^,^^110^^,^^111^

Among anaesthetic variables, propofol and ketamine were associated with higher dream recall,^23^^,^^28^^,^^42^^,^^45^^,^^46^^,^^72^^,^^91^^,^^97^, ^112^, ^113^, ^116^, ^117^, ^118^ whereas dexmedetomidine (in contrast to experimental studies) and volatile anaesthetics were associated with lower dream recall.^22^^,^^37^, ^38^, ^39^ Dose-related associations were also inconsistent, with higher propofol doses associated with increased dream recall in some studies but unrelated in others. Ketamine, volatile anaesthetics, and most other anaesthetic agents showed no systematic dose-related associations. Findings regarding adjunctive medications were also largely inconsistent.

Associations with intraoperative BIS/Patient State Index (PSI) values were mixed, although some studies found higher dream recall to be associated with deeper levels (lower BIS values) of anaesthesia.^83^^,^^90^^,^^94^^,^^100^ In contrast, higher BIS/PSI values upon awakening tended to be associated with increased dream recall.^95^^,^^101^ Findings regarding emergence time were similarly variable, with some studies reporting higher dream recall with shorter times to eye opening or faster emergence, whereas others found no significant associations.

Finally, interview timing emerged as a key methodological determinant: immediate post-emergence assessment yielded substantially higher recall rates than delayed assessments.^113^^,^^124^

Only five paediatric studies (45%) examined predictors of dream recall. Most patient-related variables showed no consistent associations. Age showed mixed results: one study reported higher dream recall in younger children,^26^ whereas another study found no association.^56^ Similarly, although one study reported that children who recalled dreams appeared calmer before induction,^56^ another found no relationship between dream recall and state anxiety.^26^ Neither the type nor duration of the procedure were significant predictors. However, one study reported that extended preoperative preparation, such as role-play, was associated with a higher likelihood of dream recall.^26^ Type of anaesthetic did not predict dream recall, and findings related to premedication and other drugs were mixed. As in adults, interviews conducted closer to emergence were associated with higher dream recall.^26^

Of the 16 experimental studies, nine (56%) examined predictors of dream recall under anaesthesia. Because several datasets overlap, only unique findings are summarised. Most studies compared dream recall rates across different anaesthetic agents, with dexmedetomidine generally associated with higher recall rates than propofol or volatile anaesthetics.^15^^,^^16^^,^^18^ Few studies assessed other predictors.

### Postoperative outcomes of anaesthesia dreaming

Only 14.6% of studies explicitly examined whether postoperative outcomes differed between patients who reported anaesthesia dreams and those who did not (see Table 2). For most outcome domains, including ICU admission, postoperative nausea and vomiting, abuse potential, pain, and other adverse events, the available evidence was insufficient to draw firm conclusions. When adequate data existed, most studies reported no significant differences between dreamers and non-dreamers in satisfaction with anaesthetic care, patient-rated quality of recovery, or postoperative anxiety or depression.

**Table 2:** Postoperative outcomes associated with anaesthesia dream recall. ↑ = increased dream recall. ↓ = decreased dream recall. n.s., not significant. ∗dreaming during anaesthesia predicted long-lasting PONV (>24 h) but not early PONV (<24 h). †Abuse potential was assessed using the Morphine–Benzedrine Group (MBG) subscale of the Addiction Research Center Inventory (ARCI) in both studies; Tezcan, and colleagues^106^ assessed at ∼15 min post-recovery, Zhao, and colleagues^31^ at 30 min and 1-week post-procedure.

Outcome domain	Association with dream recall (and number of studies providing this evidence)	Consistency of findings
**Clinical studies in adults**		
Satisfaction with anaesthetic care	n.s. (9)^23^^,^^46^^,^^83^^,^^90^^,^^94^^,^^112^^,^^105^^,^^106^^,^^108^; ↑ (2)^100^^,^^98^; ↓ (1)^36^; Rare negative appraisals reported (1)^22^	Mixed but leaning to n.s.
Willingness to repeat same anaesthesia	↑ (1)^131^; ↓ unpleasant dreams (4)^47^^,^^96^^,^^118^^,^^130^	Generally positive attitudes but negative for some of those who had negative dreams
Subjective post-operative well-being	↑^132^	Not enough evidence
Quality of recovery (patient rated)	n.s. (2)^23^^,^^94^; ↓ (1)^72^	Mixed but leaning to n.s.
Recovery speed (PACU discharge time)	n.s. (2)^94^^,^^95^; ↑ (1)^36^	Mixed but leaning to n.s.
ICU admission	↓ (1)^36^	Not enough evidence
Postoperative nausea and vomiting	↑ (1)^97^^,^∗	Not enough evidence
Postoperative anxiety and/or depression	n.s. (3)^23^^,^^46^; ↓(1)^36^	Mixed but leaning to n.s.
Intraoperative awareness	↑ (1)^34^	Not enough evidence
Abuse potential^†^	↑ (2)^31^^,^^106^	Leaning to ↑
Other postoperative adverse events	n.s. (2)^36^^,^^46^	Consistently n.s
**Clinical studies in children**		
Satisfaction/family preference	↓ unpleasant dreams (1)^49^	Not enough evidence
Postoperative behavioural disturbances	n.s. (1)^26^	Not enough evidence
Postoperative pain	n.s. (1)^26^	Not enough evidence

An important exception concerns dream valence. Although most patients who experienced dreams, including some with unpleasant content, expressed general satisfaction with their anaesthetic care, willingness to undergo the same anaesthetic again was lower among those who experienced unpleasant dreams.^47^^,^^96^^,^^118^^,^^130^

Several studies classified dreaming itself as an adverse event.^115^ This interpretation appears largely driven by earlier ketamine-based sedation studies, where unpleasant dreams were more common,^96^^,^^118^^,^^130^ or by studies that conceptualised dream reports as possible indicators of intraoperative awareness or insufficient anaesthetic depth.^29^^,^^38^ Although concerns have been raised about anaesthesia dreaming as a marker of possible awareness,^5^ studies that explicitly examined this relationship found no differences in awareness between patients who reported dreams and those who did not.^87^

Paediatric data were particularly sparse. Only two studies examined postoperative outcomes related to dreaming in children,^26^^,^^49^ precluding any conclusions.

### Neural correlates of anaesthesia dreaming

Only seven studies specifically examined neural correlates of dreaming or disconnected consciousness during anaesthesia, six of which were experimental and one clinical (see Table 3).

**Table 3:** Neural correlates of dreaming (disconnected consciousness) during anaesthesia). ACC, anterior cingulate cortex; CC, connected consciousness; DC, disconnected consciousness; EEG, electroencephalography; eLORETA, exact low-resolution brain electromagnetic tomography; Fz, frontal midline electrode; L-IFG, left inferior frontal gyrus; L-STG, left superior temporal gyrus; LZC, Lempel-Ziv complexity; MCC, middle cingulate cortex; NSTE, normalised symbolic transfer entropy; Oz, occipital midline electrode; PCC, posterior cingulate cortex; R-IFG, right inferior frontal gyrus; R-STG, right superior temporal gyrus; SEF95, spectral edge frequency 95%; SWA, slow-wave activity; UC, unconsciousness; wPLI, weighted phase lag index.

Study	Condition/Task	Neural Measurement	Time Period Analysed	Neural Correlates of Dreaming (Disconnected Consciousness)	Key Findings
Casey and colleagues^18^	Serial awakening paradigm during: Dexmedetomidine i.v. sedation (training set); propofol i.v. sedation, and natural sleep (validation set)	High-density EEG (256 channels) with source reconstruction (eLORETA)	Last 20s before awakening	For both dexmedetomidine and propofol: DC *vs* CC (markers of sensory disconnection): • Increased SWA (0.5–1 Hz) and delta (1–4 Hz) activity in fronto-medial cortices and superior portion of central sulcus; • increased theta (4–8 Hz) in right hemisphere cuneus/precuneus; • decreased beta (18–25 Hz) in right temporal lobe; • decreased gamma (28–55 Hz) in posterior temporal and frontal regions; • lower beta/delta and gamma/delta ratios widespread but especially in ventral and posterior temporal areas. UC *vs* DC (markers of unconsciousness): • Decreased beta/delta ratio in medial anterior and posterior cortices as well as in lateral left hemispheric parietal cluster (corresponding to ACC/MCC/PCC)	DC (compared with CC) shows widespread increases low-frequency activity and decreases in high-frequency power. Findings generalise across dexmedetomidine and propofol.
Casey and colleagues (2024)^19^ *Note: Reanalysis of the same dataset from Casey, and colleagues*^18^^,^^65^	Serial awakening paradigm during: Dexmedetomidine i.v. sedation (training set); propofol i.v. sedation, and natural sleep (validation set)	High-density EEG (256 channels) – evaluated 10 previously published resting-state EEG markers	Last 20s before awakening	DC *vs* CC (markers of sensory disconnection): • Decreased front-to-back NSTE (dexmedetomidine and propofol); • Decreased NSTA asymmetry (propofol) • Decreased LZC (dexmedetomidine but not propofol); • Decreased permutation entropy in gamma (dexmedetomidine) or increased permutation entropy in beta (propofol); • Increased Fz/Oz alpha power (dexmedetomidine but not propofol); • Increased frontal alpha wPLI (dexmedetomidine but not propofol); • Decreased spectral exponent (dexmedetomidine but not propofol); • Decreased SEF95 (dexmedetomidine but not propofol). UC *vs* DC (markers of unconsciousness): • Decreased spectral exponent (propofol and dexmedetomidine); • Decreased permutation entropy in the delta, beta, alpha, theta range for propofol but in the gamma, beta, alpha, theta range for dexmedetomidine; • Decreased LZC (propofol but not dexmedetomidine); • Increased frontal alpha wPLI (dexmedetomidine but not propofol) • Decreased SEF95 (dexmedetomidine but not propofol) • No association with NSTE; • No association with Fz/Oz alpha power.	Most markers associated with consciousness were also associated with connectedness. Most consistent marker for loss of connectedness was NSTE (front to back). Most consistent markers for loss of consciousness were spectral exponent and permutation entropy (mid-frequency bands).
Casey and colleagues^65^	Auditory roving oddball paradigm: Baseline (pre-drug); Dexmedetomidine i.v. sedation	High-density EEG (256 channels) with Dynamic Causal Modelling (DCM) of auditory network	0–400 ms post-stimulus onset	ERP findings (DC *vs* CC): • Emergence of a P3 in response to standard tones (absent during CC); • Decreased amplitude of N2 and P3 amplitude in response to oddballs (DC associated with a loss of discriminability between standard and oddball tones); • No significant difference between standard *vs* oddballs tones during DC. DCM effective connectivity (standards DC *vs* CC): • Increased feedback connectivity in all feedback connections; • Decreased feedforward L-STG to L-IFG; • Decreased lateral: R-STG to L-STG. DCM effective connectivity (oddball modulation DC *vs* CC): • Increased in most feedforward connections; • Decreased feedback connectivity: F-IFG to R-STG; • Decreased lateral: L-STG to R-STG.	During DC there is a disruption of normal predictive coding processes, so that all incoming auditory stimuli (both standards and oddballs) become similarly surprising, eliminating normal habituation to repeated tones.
Sarasso and colleagues (2015)^60^	TMS perturbation during: Wakefulness (Ramsay score 2); Deep unresponsiveness (Ramsay score 6) with propofol i.v., xenon inhalation, or ketamine i.v. anaesthesia	TMS-evoked EEG potentials (TEPs) with 60-channel EEG. Perturbational Complexity Index (PCI) computed from spatiotemporal activation matrices	0-400 ms post-TMS	Propofol: No dream experiences reported upon awakening (0/6 participants) UC associated with: • Low-amplitude, low-frequency positive-negative TEPs; • Local cortical activation that did not propagate from stimulation site; • Low PCI; • Increased SWA, theta, sigma; • Loss of integration (spatially restricted activation). Xenon: No dream experiences reported upon awakening (0/6 participants; 1 participant reported vague impression just before awakening with no explicit recall) UC associated with: • High-amplitude stereotypical positive-negative TEPs; • Global, long-lasting cortical spread from fixed maximum; • Low PCI; • Increased SWA, theta; decreased gamma; • Loss of differentiation (widespread stereotypical slow wave). Ketamine: All participants (6/6) reported dreams. DC associated with: • Fast-frequency recurrent TEPs resembling wakefulness; • Complex spatiotemporal activation with shifting maximum; • High PCI, comparable to those obtained during wakefulness; • Increased SWA (0.5–4.5 Hz), theta, gamma; • Preserved integration and differentiation.	PCI dissociates consciousness from responsiveness: Low PCI during propofol/xenon correlated with no dream reports (UC); High PCI during ketamine correlated with vivid dream reports (DC). DC during ketamine anaesthesia associated with both integration and differentiation.
Colombo and colleagues (2019)^61^ *Note: Reanalysis of the same dataset from Sarasso, and colleagues*^60^	Resting EEG during: Wakefulness (Ramsay score 2); Deep unresponsiveness (Ramsay score 6) with propofol i.v., xenon inhalation, or ketamine i.v. anaesthesia	Resting EEG spectral exponent (β) decay rate of power spectral density background	5 min of clean resting EEG	Spectral exponent (β) changes during anaesthesia: Broad-band (1-40Hz): • Propofol and xenon (no dream reports): large decrease (steeper PSD), β more negative • Ketamine (dream reports): no change (similar to wakefulness) Low-band (1-20Hz): • Propofol and xenon (no dream reports): large decrease (steeper PSD) • Ketamine (dream reports): small decrease (trend only) High-band (20-40Hz): • Propofol and xenon (no dream reports): decreased (steeper PSD) • Ketamine (dream reports): increased (flatter PSD) Strong correlation between spectral exponent (β) and PCI	Preservation of shallow PSD decay (similar to wakefulness) is a marker of dreaming during ketamine. Steep PSD decay during propofol/xenon is a marker of no dreams.
Darracq and colleagues (2018)^63^ *Note: Reanalysis of the same dataset from Sarasso, and colleagues*^60^	TMS-EEG during: Wakefulness; REM sleep; NREM sleep; Ketamine i.v. anaesthesia; Propofol i.v. anaesthesia	TMS-evoked EEG power in alpha (8-12Hz) and low-gamma (30-40Hz) bands	0-400 ms post-TMS over posterior cortex	DC (*vs* wakefulness): • Decreased evoked alpha power (ketamine – dream reports) UC (*vs* wakefulness): • Decreased evoked low-gamma power (propofol – no dream reports)	Suppression of evoked alpha power is a marker of dreaming (DC), whereas suppression of evoked low-gamma power is a marker of unconsciousness (UC).
Leslie and colleagues (2009)^95^	Clinical sedation using either propofol TCI or propofol + desflurane	BIS	30s segments at: (1) the middle of the operation, (2) completion of wound closure, (3) eye opening, and (4) a sequence of times (-1 min, -2 min,-3 min . . . up to-20 min) before the dream interview	Dreaming associated with: • Decreased slope at wound closure • Increased log spectral power at 30 Hz at wound closure (trend only) • Increased high-frequency activity (30 Hz) 13-17 min and 1-2 min before emergence • Fewer lower-frequency spindles (10.68 Hz) 1-5min before emergence	Dreaming is associated with more high-frequency activity and fewer spindles before emergence

Compared with connected consciousness, disconnected consciousness (dreaming) during dexmedetomidine and propofol sedation showed increased slow-wave/delta activity in fronto-medial cortices, increased theta in right cuneus/precuneus, and decreased beta and gamma in temporal and frontal regions. These patterns were consistent across both agents.^18^ Disconnected consciousness was further characterised by reduced directed information flow from frontal to posterior regions^19^ and elimination of normal evoked-response potential discriminability between standard and deviant tones, accompanied by altered feedback connectivity within a frontotemporal subnetwork.^65^

Compared with unconsciousness, disconnected consciousness (dreaming) was consistently associated with relatively preserved high-frequency activity and cortical complexity, reflected in higher beta/delta ratios,^18^ increased 30 Hz activity and fewer spindles before awakening,^95^ less steep spectral exponent, and higher mid-frequency permutation entropy.^19^ Three TMS-EEG studies analysing the same dataset further showed that dreaming with ketamine produced greater Perturbational Complexity Index values with wake-like spatiotemporal dynamics,^60^ spectral exponent similar to wakefulness,^61^ preserved evoked low-gamma power,^63^ whereas propofol and xenon (no dreams) produced localised TMS-evoked responses^60^ and steep spectral exponent decay.^61^ However, because dreaming occurred exclusively with ketamine in these studies, it is difficult to disentangle agent effects from dreaming *per se*.

Four additional studies examined neural activity during anaesthesia but had important limitations for identifying neural correlates specific to dreaming, including unclear differentiation between connected and disconnected states,^62^ analysis of responsiveness rather than dreaming,^66^ and grouping dreamers with non-dreamers under a single disconnectedness category.^17^^,^^59^

## Discussion

This scoping review synthesised over a century of research on anaesthesia dreaming, encompassing 157 studies with 87 866 participants across clinical and experimental settings.

### Key findings and insights

Anaesthesia dreaming is more common than clinical practice suggests. Ketamine and propofol were most consistently associated with higher recall than volatile anaesthetics in both cross-study and within-study comparisons,^28^^,^^113^^,^^117^ though other factors such as anaesthetic depth, emergence characteristics, emergence environment, and patient factors may also contribute. Dexmedetomidine remains comparatively understudied, with variable recall across clinical^37^^,^^39^ and experimental settings.^16^^,^^66^ The striking disparity between large clinical trials (typically 3%–8% recall^25^^,^^33^^,^^34^) and experimental studies (often 40%–80% recall^15^, ^16^, ^17^) likely reflects memory fragility, rather than differences in actual dream occurrence. Clinical settings often involve multimodal anaesthetic regimens, delayed assessment, and substantial interference during emergence, factors largely absent in experimental settings with single agents, immediate assessment, controlled emergence environments, and priming through consent. The substantial heterogeneity in study designs, measurement approaches, and control for potential confounders limits definitive causal attribution of dream experiences to specific pharmacological agents.

Across settings, populations, and agents, anaesthesia dreams were predominantly pleasant, featuring everyday autobiographical content.^23^^,^^83^^,^^90^ Even ketamine, despite being associated with distinctive phenomenology, was often described positively by patients.^42^^,^^68^^,^^70^^,^^73^ This contradicts early characterisations of anaesthesia dreaming as inherently distressing^133^ and suggests potential value in facilitating rather than suppressing these experiences. The enhanced positivity of anaesthesia dreams may reflect altered emotional processing under anaesthesia,^134^ contrasting with the negativity bias often described in sleep dreams,^135^ although the extent of this bias depends strongly on how dream affect is assessed.^136^^,^^137^ Distinct anaesthetic-specific phenomenological profiles emerged. Propofol and dexmedetomidine dreams were typically simple, static, and not particularly strange, resembling non-rapid eye movement (non-REM) sleep dreams in character and emotional intensity. ^24^ In contrast, ketamine dreams were more complex, dynamic, and bizarre, ^60^^,^^72^ potentially paralleling REM sleep dream phenomenology,^138^ though this requires systematic investigation. This pharmacological specificity suggests that distinct patterns of brain dynamics underlie distinct forms of subjective experience.^139^

Two predictors showed remarkable consistency: higher baseline home dream recall frequency^23^^,^^83^^,^^94^ and preoperative suggestion or priming.^92^^,^^110^^,^^111^ The former parallels sleep research showing stable trait differences in dream recall,^140^ suggesting common memory mechanisms across sleep and anaesthesia. Priming effects show that top-down cognitive processes, such as expectations, attentional set, and intentions, influence dream recall. This aligns with evidence from sleep research that pre-sleep experiences and instructions can influence dream content and recall.^141^ Interview timing was also a critical factor, with immediate assessment consistently yielding higher recall than delayed assessment.^113^^,^^124^ Dream memories fade rapidly, mirroring the well-established rapid forgetting of sleep dreams.^142^^,^^143^ This rapid decay likely reflects both fragile encoding and consolidation during altered brain states and difficulties with retrieval because of interference from the emergence environment.^144^

Although research on how anaesthesia dreaming relates to postoperative outcomes is scarce, one consistent pattern emerged. Those who experienced unpleasant dreams were less willing to undergo the same anaesthetic procedure again.^47^^,^^96^^,^^118^^,^^130^ Thus, subjective experiences during anaesthesia may influence patients' future healthcare decisions, even when objective outcomes are comparable. Importantly, dream recall was not associated with intraoperative awareness, challenging interpretations of dreaming as a marker of inadequate anaesthetic depth.^23^^,^^34^

Emerging evidence, albeit limited, suggests that dreaming during anaesthesia has neural signatures distinct from both unconsciousness and connected consciousness. This parallels findings from sleep dreaming^145^^,^^146^ and suggests that anaesthesia encompasses dissociable states rather than a unitary ‘unconscious’ state.^1^^,^^3^

### Critical gaps and future directions

The field’s most fundamental challenge is conceptual. Only 9.2% of clinical studies explicitly defined ‘dreaming,’ and among those that did, definitions were applied inconsistently. Moreover, the boundary between dreaming and awareness may not always be binary. Dreams incorporating environmental elements may reflect pre-anaesthesia memories and concerns or the processing of external stimuli during anaesthesia. Distinguishing the origin of such dreams is both clinically and theoretically important. Future research must employ clear operational definitions, drawing on consciousness science^1^ and sleep-dreaming literature.^142^^,^^143^ Building on established frameworks, it is important to apply refined classification systems that distinguish unconsciousness from the continuum of conscious experiences: fully disconnected (purely internally generated dream experiences, including memory incorporation), partially connected (stimulus-incorporation dream experiences), and fully connected (awareness).^1^^,^^23^^,^^24^

Methodologically, the field needs validated and standardised assessment instruments capturing the rich phenomenology of anaesthesia dreams: sensory modalities, narrative coherence, emotional valence, bizarreness, agency, sense of self, and time perception.^142^^,^^147^ The modified Brice questionnaire could serve as a starting point but should be expanded to characterise these phenomenological features,^15^^,^^24^ incorporating optimal interview techniques from sleep-dreaming research.^147^ Immediate post-emergence assessment (within 5–15 min) should become standard to minimise memory decay and interference. Studies should also systematically examine how emergence characteristics (e.g. speed, environmental stimuli, and pain levels) interfere with dream memory consolidation. Routine raw or processed EEG monitoring would enable investigation of how dream experiences relate to sedation depth across anaesthetic agents, paralleling polysomnography’s role in sleep research.^145^^,^^146^

Mechanistically, future research should use multimodal neuroimaging to compare neural dynamics preceding dream and no-dream reports under controlled conditions. Serial awakening paradigms have proved successful both in anaesthesia^18^ and sleep-dreaming research.^145^^,^^146^ Critically, serial awakening paradigms combined with continuous EEG could help determine when dreams actually occur (during maintenance, gradual emergence, or only moments before awakening) and whether emergence involves transitions through physiological sleep-like brain states or follows distinct neural trajectories. Understanding whether anaesthesia dreams arise from sleep-like *vs* anaesthesia-specific mechanisms has important implications for interpreting phenomenological similarities between anaesthesia and sleep dreams and for determining whether dreams reflect experiences during surgical anaesthesia or only emergence phenomena.

However, to bridge the gap between experimental and clinical findings, experimental protocols should also incorporate clinically realistic anaesthetic protocols. This includes systematic manipulation of induction speed, maintenance duration, emergence characteristics, and multimodal drug combinations to understand how these variables impact dream recall and content.

The phenomenological similarities between anaesthesia and sleep dreams suggest common generative mechanisms across pharmacologically and naturally occurring dreams. However, the enhanced positivity of anaesthesia relative to sleep dreams indicates that emotional processes may be differentially regulated. Systematic comparison using standardised phenomenological and neural measures is needed to understand the mechanisms underlying these similarities and differences.

Key populations remain understudied, particularly children (only 11 studies), older adult patients, and those with psychiatric conditions who may be vulnerable to distressing experiences or could potentially benefit from positive experiences. Future paediatric research should adopt developmentally informed methods adapted from paediatric sleep and dream research, potentially incorporating visual aids, simplified language, or parent-assisted recall for younger ages.^148^^,^^149^

Regarding anaesthetic contexts, dexmedetomidine remains understudied despite growing clinical use. Also, the impact of adjunctive medications and other psychotropic substances on anaesthesia dream recall and phenomenology remains largely unknown.

The predominantly pleasant nature of anaesthesia dreams, priming effects, and preliminary evidence of sustained therapeutic benefits in trauma-related conditions^32^^,^^150^ raise the question of whether anaesthesia dream experiences could be intentionally harnessed for clinical benefit. We recently conducted a feasibility study showing that a standardised multicomponent protocol incorporating verbal priming, propofol-based anaesthesia, EEG-guided emergence, minimal stimulation during pre-emergence, and immediate post-emergence interviews achieved 93% dream recall, with predominantly positive experiences and no intraoperative awareness,^151^ suggesting that systematic facilitation of anaesthesia dreaming in clinical settings is both feasible and safe. Future research should examine whether anaesthesia dream experiences may lead to reductions in mental health symptoms, examine mechanisms linking these dream experiences to lasting positive changes, identify patient populations most likely to benefit, and establish ethical guidelines for intentional manipulation of subjective states.

### Limitations

The heterogeneity in definitions, methods, and reporting limited our ability to draw firm conclusions. Some experimental studies involved overlapping datasets. More fundamentally, all dreaming research relies on subjective self-reports. Dreams cannot be directly observed or objectively verified, making it impossible to distinguish whether absent dream reports reflect absence of occurrence or failure of memory encoding, consolidation, or retrieval, or to determine when during the anaesthetic period dreams occur. Self-reports are vulnerable to memory distortion, confabulation, social desirability bias, demand characteristics, and individual differences in introspective and language abilities.^137^^,^^142^^,^^147^ Despite these constraints, self-report remains the only method for accessing subjective phenomenology, a challenge shared across consciousness research.

## Conclusions

This scoping review shows that dreaming during anaesthesia is more common than generally assumed, typically pleasant, and characterised by pharmacologically distinct phenomenological profiles. Moving forward, the field must adopt standardised definitions and validated assessment instruments, prioritise immediate post-emergence assessment, and employ multimodal neuroimaging to elucidate how different anaesthetic agents generate distinct subjective experiences. The consistency of priming effects and the predominantly positive nature of anaesthesia dreams suggest therapeutic potential warranting rigorous investigation. By addressing these gaps with greater conceptual clarity and methodological rigour, future research can advance our understanding of subjective experiences during anaesthesia, with implications for clinical practice, ethical issues related to patient care, and theoretical frameworks of consciousness.

## Authors’ Contributions

Conception and study design: PS

Literature search, screening, and data extraction: PS, SH

Data analysis and interpretation: PS

Tables and figures: PS

Drafting the manuscript: PS

Review and revision of the manuscript: PS, BDH

## Declaration of generative AI and AI-assisted technologies in the writing process

During the preparation of this work the author(s) used ChatGPT 5.3 (Open AI) and Claude (Anthropic) to improve readability. After using this tool/service, the authors reviewed and edited the content as needed and take full responsibility for the content of the publication.

## Declarations of Interest

BDH is a Scientific Adviser to Osmind Mental Health and Journey Clinical and is a consultant to Arcadia Medicine, Inc., Vida Ventures LLC, and Tactogen, LLC.

## Footnotes

‡ Sixteen studies (12.3%) classified as adult samples also included adolescents (three including participants ≥11 yr, one ≥12 yr, one ≥13 yr, two ≥14 yr, four ≥15 yr, and five ≥16 yr), and one study (0.8%) included children aged ≥4 yr (among studies that reported age ranges in addition to mean or median age). As these studies predominantly enrolled adults and analysed age groups jointly, they are presented within the adult category.
