# Supplementary material for: Dreaming during anaesthesia: a scoping review
Source: Br J Anaesth. 2026 Jun 11;137(2):506–24. doi: 10.1016/j.bja.2026.05.002 (PMC13494102; doi:10.1016/j.bja.2026.05.002)
Supplement: Supplementary file 2 [file mmc2.docx]

**Keyword Strings and Filters Used in Databases**

The following keyword strings were used in each of the databases:

Medline/PubMed:

(anesthesia[Title/Abstract] OR anaesthesia[Title/Abstract] OR anesthe*[Title/Abstract] OR anaesthe*[Title/Abstract] OR sedat*[Title/Abstract]) AND (dream*[Title/Abstract] OR disconnect*[Title/Abstract] OR “subjective experience*”)

(anesthesia[Title/Abstract] OR anaesthesia[Title/Abstract] OR anesthe*[Title/Abstract] OR sedat*[Title/Abstract]) AND (dream*[Title/Abstract] OR disconnected[Title/Abstract] OR hallucination*[Title/Abstract] OR fantas*[Title/Abstract] OR illusion*[Title/Abstract] OR nightmare*[Title/Abstract] OR sexual[Title/Abstract]) AND (adverse*[Title/Abstract] OR complications[Title/Abstract] OR "side effects"[Title/Abstract])

The search results were filtered to include studies on humans and English language articles.

EMBASE:

(anesthesia OR anaesthesia OR anesthe* OR anaesthe* OR sedat*) AND (dream* OR disconnect* OR “subjective experience*”)

(anesthesia OR anaesthesia OR anesthe* OR anaesthe* OR sedat*) AND (dream* OR disconnected OR hallucination* OR fantas* OR illusion* OR nightmare* OR sexual) AND (adverse* OR complications OR "side effects")

The search results were filtered to include studies on humans, in the English language, and the following publication types: article, conference paper, article in press, letter, short survey, chapter, note

PsycINFO:

(anesthesia OR anaesthesia OR anesthe* OR anaesthe* OR sedat*) AND (dream* OR „disconnected")

(anesthesia OR anaesthesia OR anesthe* OR anaesthe* OR sedat*) AND (dream* OR disconnected OR hallucination* OR fantas* OR illusion* OR nightmare* OR sexual) AND (adverse* OR complications OR "side effects")

Search results were filtered to include scholarly journals and articles in the English language.
