# Supplementary material for: Dreaming during anaesthesia: a scoping review
Source: Br J Anaesth. 2026 Jun 11;137(2):506–24. doi: 10.1016/j.bja.2026.05.002 (PMC13494102; doi:10.1016/j.bja.2026.05.002)
Supplement: Supplementary file 3 [file mmc3.docx]

**Table S1.** Definitions of dreaming and awareness in included studies

| **Study (Author, Year)** | **Definition of Dreaming** | **Definition of Awareness** |
| --- | --- | --- |
| Clinical Studies on Adults | | |
| Brice & Hetherington (1970)^1^ | “…any experience (excluding awareness) which a patient was able to recall and which he or she thought occurred between the induction of anesthesia and the first moment of consciousness after anesthesia.” (p. 536) | “…the ability to recall, with or without prompting, any events which occurred during the period at which it was thought the patient was fully unconscious.” (p. 535) |
| Harris et al. (1971)^2^ | “…any experience, other than awareness, which the patient was able to recall and which he or she thought occurred between the induction of anesthesia and the first moment of consciousness after the anesthetic was over.” (p. 172) | “…the ability to recall, with or without prompting, any event which occurred during the period at which it was thought that the patient was fully unconscious.” (p. 172) |
| Wilson et al. (1975)^3^ | “…a train of thoughts or images passing through the mind, which the patient believed occurred between the induction of anesthesia and the first moment of consciousness after the completion of the anesthetic procedure.” (p. 609) | “…the ability of the patient to recall, with or without prompting, any event occurring during the period when the patient was believed to be unconscious.” (p. 609) |
| Iqbal et al (1985)^4^ | – | “…the ability of a patient to recall with or without prompting any event during anesthesia…” (p.8) |
| Moore & Seymour (1987)^5^ | – | “…the factual recall of events during anesthesia…” (p. 45) |
| Bogod et al (1990)^6^ | – | “…recall of specific events, to the rare but very serious complication of full consciousness with total paralysis.” (p. 279) |
| Liu et al. (1991)^7^ | – | “…the ability of the patients to recall, with or without prompting, any event which occurred between induction of anaesthesia and recovery of consciousness at the end of anaesthesia.” (p. 435) |
| Lyons & Macdonald (1991)^8^ | – | “The patient was deemed to be aware if the content of the dream represented unequivocal recall of an event that took place in the operating theatre.” (p. 62) |
| Ghoneim et al. (2000)^9^ | – | Not explicit definition but refer to "Conscious recall of intraoperative events…" (p. 133) |
| Chaiwat et al. (2002)^10^ | – | “…spontaneous recall of events occurring under general anesthesia.”; “…explicit memory of intraoperative events, which the patient can relate to after anesthesia.” (p. 8949) |
| Vallejo et al. (2002)^11^ | “…patient recall of ideas or images unrelated to perioperative events.” (p. 428) | – |
| Yeo & Lo (2002)^12^ | – | “…the spontaneous recall of an event occurring during general anaesthesia”. (36) |
| Aceto et al. (2003)^13^ | “…a subconscious state of awareness characterized by vivid thoughts and images related to external stimuli.” (p. 631) | – |
| Myles et al. (2004)^14^ | – | “…the postoperative recollection of events occurring during general anaesthesia.” (p. 1757) |
| Sebel et al. (2004)^15^ | – | No specific definition but it has been stated that awareness was categorized if “…recalled event was confirmed by attending personnel, or the investigators were convinced that the memory was real, but no confirmation could be obtained.” (p. 834) |
| Leslie et al. (2005)^16^ | – | No specific definitions but it has been stated that “Anaesthetists also recorded any clinical signs of possible awareness, such as purposeful movement, or autonomic signs (unexplained tachycardia, hypertension, lacrimation or sweating).” (p. 240) |
| Aceto et al. (2007)^17^ | – | “…conscious recollection of intraoperative experience…” (p. 823) |
| Leslie et al. (2007)^18^ | “…any experience that was described by the patient as dreaming and was thought by the patient to have occurred between the induction of anesthesia and the first moment of consciousness after anesthesia”. (p. 35) | “…postoperative recall of intraoperative events.” (p. 35) |
| Errando et al. (2008)^19^ | – | “…when the patient stated or remembered that he or she had been awake at a time when consciousness was not intended. “(p. 178) |
| Samuelsson et al. (2008)^20^ | – | “…explicit recall of real events during intended [general anesthesia]”. (p. 805) |
| Bejjani et al (2009)^21^ | – | The paper focused on explicit memory instead of awareness per se. “Explicit memory is a conscious memory involving the intended recollection of previously learned information…” (p. 175) |
| Leslie et al. (2009)^22^ | “…any experience that was described by the patient as dreaming and was thought by the patient to have occurred between induction of anesthesia and emergence after anesthesia.” (p. 548) | – |
| Paech et al. (2009)^23^ | – | “…postoperative recall of events occurring during the period of general anesthesia.” (p. 299) |
| Xu et al. (2009)^24^ | – | “Possible awareness: There were no explicit or definite descriptions about the operation procedure, but the descriptions may be associated with the procedure, or complaints of vague memories about the operation procedure.  Awareness: There were explicit descriptions about the events or scenes during the operation procedure and could be determined as occurring in the operative period, which were not denied by surgery staff.” (p. 874) |
| Kim et al. (2011)^25^ | “…as any experience (excluding the explicit recall of intraoperative events) that was described by the patients as dreaming and was thought by the patient to have occurred between the induction of sedation and the first moment of consciousness after sedation.” (p. 1077) | – |
| Zhang et al. (2011)^26^ | – | "…possible awareness: There was no explicit or definite descriptions about the operation procedure, but the descriptions might be associated with the procedure, or complaints of vague memories about the operation procedure.  […]  …awareness: There were explicit descriptions about the events or scenes during the operation procedure and could be determined as occurring in the operative period, and were not denied by the surgery staff.” (p. 3666) |
| Czarko et al. (2013)^27^ | – | “…recall of events that occur during anesthesia. Recall can be overt (evoked spontaneously) or latent (evoked by psychological tests).” (p. 200) |
| Russell (2013)^28^ | – | “…two things are required: the patient must have been conscious during the procedure; and in the postoperative period, the patient must be able to recall that same episode of the intraoperative consciousness.” (p. 1011) |
| Chikkahanumanthappa et al. (2016)^29^ | “Dreaming during sedation was defined as any experience that was described by the patient as dreaming and was thought by the patient to have occurred between the induction of sedation and the first moment of awakening after surgery.” (p. 329) | – |
| Kabukcu et al. (2016)^30^ | – | “Awareness is the patient’s being awake during the operation, having a bad dream, or remembering some major events.” (p. 181) |
| Cascella et al. (2017)^31^ | – | “…explicit recall of sensory perceptions of patient during anesthesia.” (p. 79249) |
| Villegas et al. (2019)^32^ | – | “…when a patient can recall the surroundings or an event related to the surgery while under general anaesthesia…” (p. 557) |
| Chen et al. (2020)^33^ | “…subjective experience which is produced both physically and psychologically during physical sleeping.” (p. 2)  “…any experiences and thoughts that occur between anesthesia induction and the time of awakening.” (p. 5) | – |
| Alam et al (2022)^34^ | – | “Consciousness and subsequent explicit recall of intra-operative events, whether the experience is spontaneously reported by the patient, or detected by direct questioning or prompting.” (p. 3) |
| Ghanaie et al. (2022)^35^ | – | “…the postoperative recall of any event that occurred during surgery.” (p. 214) |
| Imantalab et al. (2022)^36^ | – | “…a condition in which the patients can recall their surroundings or an event related to the surgery. It can be reported as pain, auditory perception, loss of motor function, helplessness, and panic.” (p. 1) |
| Schäfer et al. (2022)^37^ | “…any recalled experience (excluding awareness) that occurred between induction of anesthesia and the first moment of consciousness upon emergence…” (p. 2) | – |
| Dikkala et al. (2023)^38^ | – | “Intraoperative awareness (IA), or the unexpected and explicit recall by patients of events that occurred intraoperatively…” (p. 212) |
| Clinical Studies on Children/Adolescents | | |
| McKie & Thorp (1973)^39^ | – | “An obvious correlation with events occurring, or words spoken, during the procedure.” (p. 412) |
| Hobbs et al. (1988)^40^ | “…any experience (excluding awareness) which a patient *thought* occurred between the induction of anesthesia and the first moment of consciousness after anesthesia”. (p. 560) | – |
| Huang et al. (2005)^41^ | “…any experience (excluding awareness) which a patient thought occurred between the induction of anaesthesia and the first moment of consciousness after anaesthesia.” (p. 854) | “Events that the child equated with a plausible real event…” (p. 855) |
| Experimental Studies | | |
| Noreika et al. (2011)^42^ | “…the occurrence of purely internally generated, stimulus-independent subjective experiences during the unresponsive state.” (p. 328) | “…veridical awareness of external stimuli during the unresponsive state…” (p. 328) |
| Sarasso et al. (2015)^43^ | “Experience was defined as any kind of mental activity,” which included thoughts, dreams, images, emotions, etc.” (Supplementary Materials) | – |
| Darracq et al. (2018)^44^ | “…a state in which sensory stimuli are not incorporated into conscious experience (disconnected consciousness).” (p. 1) | – |
| Radek et al (2018)^45^ | “We defined internally generated experiences as dreaming (implying disconnected consciousness)” (p. 261) | “…externally generated experiences as accurate awareness of the research environment (implying connectedness).” (p. 261) |
| Colombo et al. (2019)^46^ | “Experience was defined as “any kind of mental activity,” which included thoughts, dreams, images, emotions, etc.” (p. 633) | – |
| Radek et al. (2021)^47^ | “Purely internally-generated hallucinatory experiences, that is, reports of contents of consciousness that are not directly related to or do not originate from the research environment.” (p. 5) | “Externally-generated experiences which are related to objects/persons that have been present, or events that have occurred, during anesthetic administration, but which have been not been present beyond the confines of the administration period, and which thus cannot be memory incorporation.” (p. 5) |
| Scheinin et al. (2021)^48^ | “In a disconnected state, the contents of consciousness are seldom related to incoming sensory information and typically consist of only internally generated experiences.” (p. 1770) | “…externally generated experiences referred to awareness of the current environment (experiences related to verifiable stimuli that the participant could not have expected to occur during the experimental session).” (p.1772) |
| Casey, Tanabe, et al. (2022a)^49^ | “…disconnected consciousness […] a conscious experience but no awareness of the environment.” (p.1008) | “…connected consciousness: conscious awareness of the environment…” (p. 1008) |
| Casey et al. (2022b)^50^ | “…consciousness without sensory awareness (disconnected consciousness).” (p. 1) | “…consciousness with sensory awareness (connected consciousness)” (p. 1) |
| Kantonen et al. (2023)^51^ | “Disconnectedness may indicate total absence of internal conscious experiences, but is also consistent with the presence of internally generated, externally disconnected conscious experiences (e.g., dreams).” (p. 4885) | “Connectedness is here defined as the ability to consciously perceive external stimuli and to either perform meaningful voluntary behavioral responses or later report having experienced external events.” (p. 4885) |
| Valli et al. (2023)^52^ | “Purely internally generated hallucinatory content that was not directly related to or did not originate from the research environment.” (p. 352) | “Externally generated experiences that were related to objects/persons that had been present, or events that had occurred, during the session, but the occurrence of which the participant could not have anticipated, and which thus could not be categorised as memory incorporation.” (p. 352) |
| Casey et al. (2024)^53^ | “…consciousness […] refers to an individual's state of phenomenological experience…” “…consciousness is not limited to waking states but it can also exist when an individual is unaware of their environment, such as during dreaming sleep”. (p. 301) | “…connectedness […] refers to an individual's sensory awareness of the world around them…” (p. 301) |

**References**

1.	Brice DD, Hetherington RR, Utting JE. A simple study of awareness and dreaming during anaesthesia. *Br J Anaesth*. 1970;42(6):535-542. doi:10.1093/bja/42.6.535

2.	Harris TJB, Brice DD, Hetherington RR, Utting JE. Dreaming associated with anaesthesia: the influence of morphine premedication and two volatile adjuvants. *Br J Anaesth*. 1971;43(2):172-178. doi:10.1093/bja/43.2.172

3.	Wilson SL, Vaughan RW, Stephen CR. Awareness, dreams, and hallucinations associated with general anesthesia. *Anesth Analg*. 1975;54(5):609. doi: 10.1213/00000539-197509000-00010

4.	Iqbal KM, Hussain P, Afzalunnessa B. Study of awareness during noninhalational anaesthesia. *Bangladesh Med Res Counc Bull*. 1985;11(1):8-14.

5.	Moore JK, Seymour AH. Awareness during bronchoscopy. *Ann R Coll Surg Engl*. 1987;69(2):45-47.

6.	Bogod DG, Orton JK, Yau HM, Oh TE. Detecting awareness during general anaesthetic Caesarean section: an evaluation of two methods. *Anaesthesia*. 1990;45(4):279-284. doi:10.1111/j.1365-2044.1990.tb14732.x

7.	Liu WHD, Thorp TAS, Graham SG, Aitkenhead AR. Incidence of awareness with recall during general anaesthesia. *Anaesthesia*. 1991;46(6):435-437. doi:10.1111/j.1365-2044.1991.tb11677.x

8.	Lyons G, Macdonald R. Awareness during caesarean section. *Anaesthesia*. 1991;46(1):62-64. doi:10.1111/j.1365-2044.1991.tb09321.x

9.	Ghoneim MM, Block RI, Dhanaraj VJ, Todd MM, Choi WW, Brown CK. Auditory evoked responses and learning and awareness during general anesthesia. *Acta Anaesthesiol Scand*. 2000;44(2):133-143. doi:10.1034/j.1399-6576.2000.440202.x

10.	Chaiwat O, Vacharaksa K, Prakanrattana U. Awareness under general anesthesia. *J Med Assoc Thai*. 2002;85 Suppl 3:S948-955.

11.	Vallejo MC, Romeo RC, Davis DJ, Ramanathan S. Propofol-ketamine *versus* propofol-fentanyl for outpatient laparoscopy. *J Clin Anesth*. 2002;14(6):426-431. doi:10.1016/S0952-8180(02)00391-4

12.	Yeo SN, Lo WK. Bispectral index in assessment of adequacy of general anaesthesia for lower segment Caesarean section. *Anaesth Intensive Care*. 2002;30(1):36-40. doi:10.1177/0310057X0203000106

13.	Aceto P, Valente A, Gorgoglione M, Adducci E, De Cosmo G. Relationship between awareness and middle latency auditory evoked responses during surgical anaesthesia. *Br J Anaesth*. 2003;90(5):630-635. doi:10.1093/bja/aeg113

14.	Myles PS, Leslie K, McNeil J, Forbes A, Chan MTV. Bispectral index monitoring to prevent awareness during anaesthesia: the B-Aware randomised controlled trial. *The Lancet*. 2004;363(9423):1757-1763. doi:10.1016/S0140-6736(04)16300-9

15.	Sebel PS, Bowdle TA, Ghoneim MM, et al. The incidence of awareness during anesthesia: a multicenter United States study. *Anesth Analg*. 2004;99(3):833. doi:10.1213/01.ANE.0000130261.90896.6C

16.	Leslie K, Myles PS, Forbes A, Chan MTV, Swallow SK, Short TG. Dreaming during anaesthesia in patients at high risk of awareness. *Anaesthesia*. 2005;60(3):239-244. doi:10.1111/j.1365-2044.2004.04087.x

17.	Aceto P, Congedo E, Lai C, Valente A, Gualtieri E, De Cosmo G. Dreams recall and auditory evoked potentials during propofol anaesthesia. *NeuroReport*. 2007;18(8):823-826. doi:10.1097/WNR.0b013e3280e129f5

18.	Leslie K, Skrzypek H, Paech MJ, Kurowski I, Whybrow T. Dreaming during anesthesia and anesthetic depth in elective surgery patients: a prospective cohort study. *Anesthesiology*. 2007;106(1):33-42. doi:10.1097/00000542-200701000-00010

19.	Errando CL, Sigl JC, Robles M, et al. Awareness with recall during general anaesthesia: a prospective observational evaluation of 4001 patients. *Br J Anaesth*. 2008;101(2):178-185. doi:10.1093/bja/aen144

20.	Samuelsson P, Brudin L, Sandin RH. Intraoperative dreams reported after general anaesthesia are not early interpretations of delayed awareness. *Acta Anaesthesiol Scand*. 2008;52(6):805-809. doi:10.1111/j.1399-6576.2008.01634.x

21.	Bejjani G, Lequeux PY, Schmartz D, Engelman E, Barvais L. No evidence of memory processing during propofol-remifentanil target-controlled infusion anesthesia with bispectral index monitoring in cardiac surgery. *J Cardiothorac Vasc Anesthesia*. 2009;23(2):175-181. doi:10.1053/j.jvca.2008.09.016

22.	Leslie K, Sleigh J, Paech MJ, Voss L, Lim CW, Sleigh C. Dreaming and electroencephalographic changes during anesthesia maintained with propofol or desflurane. *Anesthesiology*. 2009;111(3):547-555. doi:10.1097/ALN.0b013e3181adf768

23.	Paech MJ, Scott KL, Clavisi O, Chua S, McDonnell N. A prospective study of awareness and recall associated with general anaesthesia for caesarean section. *Int J Obstet Anesth*. 2008;17(4):298-303. doi:10.1016/j.ijoa.2008.01.016

24.	Xu L, Wu AS, Yue Y. The incidence of intra-operative awareness during general anesthesia in China: a multi-center observational study. *Acta Anaesthesiol Scand*. 2009;53(7):873-882. doi:10.1111/j.1399-6576.2009.02016.x

25.	Kim DK, Joo Y, Sung TY, Kim SY, Shin HY. Dreaming in sedation during spinal anesthesia: a comparison of propofol and midazolam infusion. *Anesth Analg*. 2011;112(5):1076-1081. doi:10.1213/ANE.0b013e3182042f93

26.	Zhang C, Xu L, Ma Y qun, et al. Bispectral index monitoring prevent awareness during total intravenous anesthesia: a prospective, randomized, double-blinded, multi-center controlled trial. *Chin Med J*. 2011;124(22):3664.

27.	Czarko K, Kwiatosz-Muc M, Fijałkowska A, Kowalczyk M, Rutyna R. Intraoperative awareness—comparison of its incidence in women undergoing general anesthesia for caeserean section and for gynaecological procedures. *Anaesthesiol Intensive Ther*. 2013;45(4):200-204. doi:10.5603/AIT.2013.0039

28.	Russell IF. The ability of bispectral index to detect intra‐operative wakefulness during isoflurane/air anaesthesia, compared with the isolated forearm technique. *Anaesthesia*. 2013;68(10):1010-1020. doi:10.1111/anae.12357

29.	Chikkahanumanthappa NB, Dasara SG, Pawar SD, Takkalki VV. A comparative study of sedation with dexmedetomidine or midazolam during spinal anesthesia. *Anaesth. pain intensive care*. 2016;20.

30.	Kabukcu HK, Sahin N, Ozkaloglu K, Golbasi I, Titiz TA. Bispectral index monitoring in patients undergoing open heart surgery. *Braz J Cardiovasc Surg*. 2016; 31(2):178-82. doi:10.5935/1678-9741.20160038

31.	Cascella M, Fusco R, Caliendo D, et al. Anesthetic dreaming, anesthesia awareness and patient satisfaction after deep sedation with propofol target controlled infusion: a prospective cohort study of patients undergoing day case breast surgery. *Oncotarget*. 2017;8(45):79248-79256. doi:10.18632/oncotarget.17238

32.	Villegas S, Suarez S, Owuor J, et al. Intraoperative awareness and experience with a ketamine-based anaesthesia package to support emergency and essential surgery when no anaesthetist is available. *Afr J Emerg Med*. 2019;9:S56-S60. doi:10.1016/j.afjem.2018.07.003

33.	Chen L, Zhang J, He W, Liu W. Comparative effects of dexmedetomidine and midazolam on dreaming of patients undergoing flexible bronchoscopy during general anesthesia. *Med Sci Monit*. 2020;27. doi:10.12659/MSM.929000

34.	Alam T, Qureshi R, Aziz M, Khan A. Incidence of accidental awareness during general anesthesia – a tertiary cancer care hospital experience. *J Postgrad Med Inst*. 2022;36(1):3-8. http://doi.org/10.54079/ jpmi.36.1.2996

35.	Ghanaie MM, Hosseinzadeh F, Soltanipour S, Sorouri ZR, Madani ZH, Biazar G, Dalir H, Ashrafiyeh MT. Intraoperative awareness during Cesarean delivery under general anesthesia. *Caspian J Neurol Sci*. 2022;8(4):213-221. doi:10.32598/CJNS.4.31.2

36.	Imantalab V, Mohammadzadeh Jouryabi A, Sedighinejad A, et al. Intraoperative awareness in patients undergoing cardiac surgery in an academic center in the north of Iran. *Anesth Pain Med*. 2022;12(6). doi:10.5812/aapm-131795

37.	Schäfer B, Klose P, Wiltfang J, Fudickar A. Incidence of dreaming and subjective quality of general anesthesia with and without standardized stress relaxation and dream suggestion by the anesthesiologist: A prospective interventional randomized trial using an adaptive design. *Dreaming*. 2023;33(3):264-274. doi:10.1037/drm0000215

38.	Dikkala DB, Vamshi Yaswanth, Guntreddy AS. Clinical effectiveness of Bispectral Index to reduce the risk of awareness for elective surgical patients under general anesthesia: an observational study. *Asian J Pharm Clin Res*. 2023:212-215. doi:10.22159/ajpcr.2023.v16i12.49940

39.	McKie DBD, Thorp EA. Awareness and dreaming during anaesthesia in a paediatric hospital. Anaesth Intensive Care 1973; 1: 404–14. doi: 10.1177/0310057X7300100507

40.	Hobbs AJ, Bush GH, Downham DY. Peri‐operative dreaming and awareness in children. *Anaesthesia*. 1988;43(7):560-562. doi:10.1111/j.1365-2044.1988.tb06687.x

41.	Huang GH, Davidson AJ, Stargatt R. Dreaming during anaesthesia in children: incidence, nature and associations. *Anaesthesia*. 2005;60(9):854-861. doi:10.1111/j.1365-2044.2005.04259.x

42.	Noreika V, Jylhänkangas L, Móró L, et al. Consciousness lost and found: subjective experiences in an unresponsive state. *Brain Cogn*. 2011;77(3):327-334. doi:10.1016/j.bandc.2011.09.002

43.	Sarasso S, Boly M, Napolitani M, et al. Consciousness and complexity during unresponsiveness induced by propofol, xenon, and ketamine. *Curr Biol*. 2015;25(23):3099-3105. doi:10.1016/j.cub.2015.10.014

44.	Darracq M, Funk CM, Polyakov D, et al. Evoked alpha power is reduced in disconnected consciousness during sleep and anesthesia. *Sci Rep*. 2018;8(1):16664. doi:10.1038/s41598-018-34957-9

45.	Radek L, Kallionpää RE, Karvonen M, et al. Dreaming and awareness during dexmedetomidine- and propofol-induced unresponsiveness. *Br J Anaesth*. 2018;121(1):260-269. doi:10.1016/j.bja.2018.03.014

46.	Colombo MA, Napolitani M, Boly M, et al. The spectral exponent of the resting EEG indexes the presence of consciousness during unresponsiveness induced by propofol, xenon, and ketamine. *Neuroimage*. 2019;189:631-644. doi:10.1016/j.neuroimage.2019.01.024

47.	Radek L, Koskinen L, Sandman N, et al. On no man’s land: subjective experiences during unresponsive and responsive sedative states induced by four different anesthetic agents. *Conscious Cogn*. 2021;96:103239. doi:10.1016/j.concog.2021.103239

48.	Scheinin A, Kantonen O, Alkire M, et al. Foundations of human consciousness: imaging the twilight zone. *J Neurosci*. 2021;41(8):1769-1778. doi:10.1523/JNEUROSCI.0775-20.2020

49.	Casey CP, Tanabe S, Farahbakhsh Z, et al. Distinct EEG signatures differentiate unconsciousness and disconnection during anaesthesia and sleep. *Br J Anaesth*. 2022;128(6):1006-1018. doi:10.1016/j.bja.2022.01.010

50.	Casey CP, Tanabe S, Farahbakhsh Z, et al. Dynamic causal modelling of auditory surprise during disconnected consciousness: the role of feedback connectivity. *NeuroImage*. 2022;263:119657. doi:10.1016/j.neuroimage.2022.119657

51.	Kantonen O, Laaksonen L, Alkire M, et al. Decreased thalamic activity Is a correlate for disconnectedness during anesthesia with propofol, dexmedetomidine and sevoflurane but not S-ketamine. *J Neurosci*. 2023;43(26):4884-4895. doi:10.1523/JNEUROSCI.2339-22.2023

52.	Valli K, Radek L, Kallionpää RE, et al. Subjective experiences during dexmedetomidine- or propofol-induced unresponsiveness and non-rapid eye movement sleep in healthy male subjects. *Br J Anaesth*. 2023;131(2):348-359. doi:10.1016/j.bja.2023.04.026

53.	Casey CP, Tanabe S, Farahbakhsh ZZ, et al. Evaluation of putative signatures of consciousness using specific definitions of responsiveness, connectedness, and consciousness. *Br J Anaesth*. 2024;132(2):300-311. doi:10.1016/j.bja.2023.09.031
