# Supplementary material for: Dreaming during anaesthesia: a scoping review
Source: Br J Anaesth. 2026 Jun 11;137(2):506–24. doi: 10.1016/j.bja.2026.05.002 (PMC13494102; doi:10.1016/j.bja.2026.05.002)
Supplement: Supplementary file 4 [file mmc4.docx]

**Table S1.** Overview of studies included in the scoping review, including clinical studies on adults (N = 130), clinical studies on children/adolescents (N = 11), and experimental studies (N = 16).

| **Study (Author, Year)** | **Focus on dreaming?** | **Country** | **Study design** | **Sample size** | **Age** | **Sex** | **Type of procedure** | **Induction anaesthetics** | **Maintenance anaesthetics** | **Use of BIS or EEG** | **Time of interview** | **What was asked?** | **Frequency of dreaming** | **Frequency of awareness** |
| --- | --- | --- | --- | --- | --- | --- | --- | --- | --- | --- | --- | --- | --- | --- |
| Clinical Studies on Adults | | | | | | | | | | | | | | |
| Smith (1921)^1^ | no | UK | Case study | 1 | - | F | Tonsil and adenoid removal | Ether | - | - | - | - | 1 dream | - |
| Hutchinson (1960)^2^ | no | UK | Prosp. obs. | 656 | - | - | General, emergency, gynaecological, and neurosurgical | - | - | - | - | “Did you dream during your operation?” | 2.9% | 1.2% |
| Brice et al. (1970)^3^ | yes | UK | Prosp. obs. | 57 | M = 48.3;  Range:  20 – 77 | 56% F, 44% M | General surgical and gynaecological | Thiopentone sodium | N₂O/O₂ | - | (1) a few minutes post-op;  (2) 24-48 h post-op;  (3) 7-8 days post-op | A few minutes post-op, patients were asked if they had dreamed. If the answer was yes, the patient was asked to give a details about the dreams.  Interviews were conducted 24-48h and 7-8 days postop:  1. "What was the last thing you remembered happening before you went to sleep?"  2. "What is the first thing you remember happening on waking?"  3. "Did you dream or have any other experiences whilst you were asleep?"  4. "What was the worst thing about your  operation?"  5. "What was the next worst?" | 43.8% | 1.8% |
| Harris et al. (1971)^4^ | yes | UK | RCT (Partial) | 120  (4 groups) | Group 1:  M = 51 | 46% F, 54% M | General surgical, orthopaedic,  and gynaecological procedures | Group 1 Thiopentone sodium | Group 1:  N₂O/O₂ | - | (1) Immediately post-op;  (2) 24-48hr post-op | Brice interview (Brice et al., 1970):  1. “What was the last thing you remembered happening before you went to sleep?"  2. "What is the first thing you remember happening on waking?"  3. "Did you dream or have any other experiences whilst you were asleep?”  4. "What was the worst thing about your operation?"  5. "What was the next worst?" | Group 1: 57% | 0% |
|  |  |  |  |  | Group 2:  M = 49 |  |  | Group 2 Thiopentone sodium + Morphine before induction | Group 2 (Premedication with Morphine): N₂O/O₂ |  |  |  | Group 2: 23% |  |
|  |  |  |  |  | Group 3:  M = 48 |  |  | Group 3: Thiopentone sodium | Group 3: Halothane (0.3-0.5%) |  |  |  | Group 3: 0% |  |
|  |  |  |  |  | Group 4:  M = 51 |  |  | Group 4: Thiopentone sodium | Group 4: Methoxyflurane (0.1-0.3%) |  |  |  | Group 4: 23% |  |
| Smith et al. (1972)^5^ | yes | UK | RCT (Partial) | 90 | - | - | General surgical, orthopaedic or gynaecological procedures | Thiopentone sodium | Group 1: N₂O/O₂ | - | 24-48 h post-op | - | Group 1: 33% | 2.2% |
|  |  |  |  |  |  |  |  |  | Group 2: N₂O/O₂  + Halothane (0.5%) for the first 15min |  |  |  | Group 2: 17% |  |
|  |  |  |  |  |  |  |  |  | Group 3: N₂O/O₂+ Halothane (0.5%) for the last 15-30 min |  |  |  | Group 3: 20% |  |
| Crawford & Davies (1975)^6^ | no | UK | Quasi-RCT | 405 | - | 100% F | Caesarean section (elective or emergency) | Thiopentone | Group 1: N₂O/O₂ + Methoxyflurane (0.1%) | - | 1^st^ post-op day | No specific questions reported. Each patient was asked whether or not she recollected dreaming during the operation period. | Group 1: 2.4% | Group 1: 1.8% |
|  |  |  |  |  |  |  |  |  | Group 2: N₂O/O₂ + Trichloroethylene (0.1%) |  |  |  | Group 2: 3.2% | Group 2: 5.7% |
| Hejja & Galloon (1975)^7^ | yes | Canada | RCT | 150 | - | 100% F | Surgical abortion | Ketamine | Ketamine | - | Within 24 h post-op | No specific questions reported. | 34.7% | - |
| Wilson et al. (1975)^8^ | yes | USA | Prosp. obs. | 490 | M = 44.4; Range:  15 – 89 | 65% F, 35% M | General surgical, Gynaecologic, Orthopaedic, Plastic, Oral, Genitourinary | N₂O/O₂: 100%  Halothane: 64% Narcotics: 19% Enflurane: 9% Innwar@: 4% Ketamine: 2% N₂O/O₂ - + relaxant: 0.2% Other: 1.8% | | - | (1) 24 h post-op  (2) 48-72 h post-op | Modified Brice interview (Brice et al., 1970):  1. “What was the last thing you remembered  before going to sleep?”  2. “What was the first thing you remembered  on awakening?”  3. “Did you dream or have any other experience  while you were asleep?”  4. “What was the worst thing about your  operation?”  5. “Have you any unpleasant memories  associated with any previous anaesthesia?” | 8% | 1% |
| Abouleish & Taylor (1976)^9^ | yes | USA | Prosp. obs. | 68 | - | 100% F | Caesarean section | Thiamylal | N₂O/O₂ | - | 24-36h postop | Modified Brice interview (Brice et al., 1970):  1. “What was the last thing you remember before going to sleep?”  (2) “What was the first thing you remembered on waking?”  (3) “Do you remember anything between going to sleep and waking?”  (4) “While you were sleeping, during the operation, did you dream? If yes, was the dream pleasant, unpleasant, or indifferent?”  (5) “Do you usually remember your dreams vividly?”  […]  (8) “If you must have another operation, would you like the same anaesthetic or not, and why?” | 4.4% | 1.5% |
| Crawford et al. (1976)^10^ | no | UK | Prosp. obs. | 358 | - | 100% F | Caesarean section | Thiopentone | Methoxyflurane (0.1% or 0.2%) or Trichloroethylene (0.1% or 0.2%) + N₂O/O₂ | - | 1^st^ post-op day | No specific questions reported. Patients were asked if they had experienced pleasant or unpleasant dreams whilst under anaesthesia | 0.1 % methoxyflurane: 2.9%  0.1% trichloroethylene: 3.2%  0.2% methoxyflurane: 2.9%  0.2% trichloroethylene: 2.7% | 0.1 % |
| Downing et al. (1976)^11^ | no | South Africa | Prosp. obs.. | 50 | - | 100% F | Caesarean section | Ketamine | N₂O/O₂  before delivery, followed by the addition of Halothane (0.5%) or Enflurane (0.6%) after delivery | - | 1^st^ post-op day | - | 30% | 0% |
| Famewo et al. (1976)^12^ | yes | Nigeria | Prosp. obs. | 100 | - | 100% F | Emergency Caesarean section | Thiopentone | N₂O/O₂ | - | Within 24h post-op | Modified Brice interview (Brice et al., 1970):  1. “What was the last thing you remembered before going to sleep?”  2. “When you were asleep did you have any dreams? If so, describe the details.”  3. “How often do you dream at home - rarely, occasionally or frequently?”  4. “Were you aware of pain or any other events during the operation?”  5. “What was the first thing you remembered when you woke up?” | 17% | 4% |
| Frederickson et al. (1976)^13^ | no | USA | Prosp. open-label trial | 28 | 20-40: 43%;  0-60: 32%;  >60: 14% | 29% F, 71% M | Superficial surgeries (eg skin grafts, burn dressings, excisions, etc), orthopaedic, urologic, dilation and curettage, thoracotomy with lobectomy | Etoxadrol | Etoxadrol;  11 patients received also N₂O/O₂ | - | - | - | 75% | 0% |
| Freuchen et al. (1976)^14^ | no | Denmark | RCT (Double-Blind) | 136  (140 enrolled) | Mdn = 27;  Range: 14 – 48 | 100% F | Surgical abortion | Group A: Ketamine + flunitrazepam | Ketamine | - | 1^st^ post-op day | No specific questions reported. Patients were asked whether they remembered their dreams. | Group A:: 1.47% | - |
|  |  |  |  |  |  |  |  | Group B: Ketamine + placebo |  |  |  |  | Group B: 54.6% |  |
| Mahomedy et al. (1976)^15^ | no | South Africa | Prosp. obs. | 50 | - | 100% F | Caesarean section | Ketamine | N₂O/O₂ | - | Immediately post-op | - | 30% | 0% |
| Ellingson et al. (1977)^16^ | no | Norway | RCT | 26  (2 groups of 13) | Group 1:  M = 25; Range: 21 – 33 | 100% F | Vaginal forceps delivery | Group 1: Ketamine | Group 1: Ketamine (as needed) | - | Interviews were conducted when pts were fully conscious | No specific questions reported. Patients were asked about their experience of anaesthesia. | Group 1 (Ketamine): 76.9% | Group 1 (Ketamine): 30.8% |
|  |  |  |  |  | Group 2:  M = 24; Range: 18 – 32 |  |  | Group 2: Diazepam | Group 2: N₂O/O₂ |  |  |  | Group 2 (Diazepam): 0% | Group 2 (Diazepam): 0% |
| Figallo, Casali et al. (1977)^17^ | no | USA | RCT (Double-Blind) | 135 | Range: 21 – 35 | 100% F | Laparoscopic sterilization | Ketamine | Ketamine | - | 12h post-op | - | Overall: 34.7% | - |
| Figallo, McKenzie et al. (1977)^18^ | no | USA | Prosp. Non-RCT Trial | 64 | Overall range: 15 – 34;  Group 1: M = 22.60 ± 6.45 | 100% F | Dilatation, evacuation, and curettage | Group 1: Methohexitone | Group 1: N_2_O/O_2_ |  | Every 15 minutes | No specific questions reported. Patients were asked if they experienced dreams. | Group 1 Methohexitone + N_2_O/O_2_): 9.4% | - |
|  |  |  |  |  | Group 2: M = 22.78 ± 5.53 |  |  | Group 2: Ketamine. | Group 2: Ketamine i.v. (fractional doses) |  |  |  | Group 2 (Ketamine): 18.8% |  |
| Latto & Waldron (1977)^19^ | no | UK | Prosp. obs. | 30  (2 groups of 15) | Group 1:  M = 28.9 ± 6.5;  Range: 18 – 39 | 100% F | Elective Caesarean section | Sodium Thiopentone | Group 1: Halothane (0.65%) + N₂O/O₂ | - | 1) 1h post-op; (2) 24h post-op | No specific questions reported. Whether the patients had dreamt during the operation. | 6.7% overall  Group 1: 0% | 0% |
|  |  |  |  |  | Group 2:  M = 25.6 ± 4.3;  Range: 19 – 35 |  |  |  | Group 2: Halothane (0.2%) + N₂O/O₂ |  |  |  | Group 2: 13.3% |  |
| Tammisto & Tigerstedt (1977a)^20^ | no | Finland | Prosp. obs. | 82 | M = 46; Range: 19 – 77 | 60% F, 40% M | Biliary or gastric surgery | Thiopental + fentanyl | Fentanyl + N₂O/O₂ | - | 1^st^ post-op day | - | 0% | 0% |
| Tammisto & Tigerstedt (1977b)^21^ | no | Finland | Prosp. obs. | 113 | M = 48; Range: 20 – 75 | 47.8% F, 52.2% M | Biliary or gastric surgery | Thiopental | Thiopental + Halothane (0.5%) + N₂O/O₂ | - | 1^st^ post-op day | - | 5% | 0.9% |
| Dunn et al. (1978)^22^ | no | Canada | RCT (Double-Blind) | 54  (Group 1: 30; Group 2: 24) | Group 1: M = 22.2 ± 3.6 | 100% F | Minor gynaecological surgery | Group 1: Fentanyl + Thiopentone | Group 1: Thiopentone + N₂O/O₂ | - | 1^st^ post-op day | A questionnaire was designed to assess the occurrence of dreams, but no specific questions reported. | Group 1: 27% | - |
|  |  |  |  |  | Group 2: M = 23.0 ± 3.5 |  |  | Group 2: Fentanyl + Alfathesin | Group 2: Alfathesin + N₂O/O₂ |  |  |  | Group 2: 0% |  |
| Drummond et al. (1979)^23^ | no | Canada | RCT (Double-Blind) | 111  (postop questionnaire responses received from 103) | Range: 18 – 35 | 100% F | Surgical abortion | Ketamine | Ketamine | - | 1^st^ post-op day | - | 65% | - |
| Mattila et al. (1979)^24^ | yes | Finland | RCT (Double-Blind) | 109  (Group 1: 55; Group 2: 54) | M = 28; Range: 14 – 49 | 100% F | Abortion or diagnostic curettage | Group 1: Ketamine + Diazepam | Ketamine (as needed) | - | 6 h post-op | No specific questions reported. Patients were interviewed using a systematic dream-interview. | Group 1 (Diazepam group): 41.8% | - |
|  |  |  |  |  |  |  |  | Group 2: Ketamine |  |  |  |  | Group 2 (Placebo group): 72.2% |  |
| White et al. (1980)^25^ | no | USA | RCT (Double-Blind) | 60  (Group 1: 20; Group 2: 21; Group 3: 19) | Range: 18 – 68 | - | Elective outpatient surgery (minor operations) | Group 1: Racemic ketamine (2mg/kg) | Group 1: Ketamine bolus (1mg/kg) as needed | - | As soon as possible in the recovery room | - | Overall: 84%  Group 1 (Racemic ketamine): 85% | 0% |
|  |  |  |  |  |  |  |  | Group 2:  S-Ketamine (1mg/kg) | Group 2: Ketamine bolus (0.5 mg/kg) as needed |  |  |  | Group (S-Ketamine): 84% |  |
|  |  |  |  |  |  |  |  | Group 3:  R-Ketamine (3 mg/kg) | Group 3: Ketamine bolus (1.5 mg/kg) as needed |  |  |  | Group 3 (R-Ketamine): 83% |  |
| Purdell-Lewis et al. (1981)^26^ | no | Canada | RCT | 99 | - | - | A variety of surgical procedures; not specified | Fentanyl + Thiopentone | N₂O/O₂ + Fentanyl | - | Within the first two post-op days | No specific questions reported. Patients were asked about the last event before induction of anaesthesia, dreaming, first memory on awakening and willingness to accept this type of anaesthesia again. | 8.2% | 1.0 - 2.0% |
| Dich-Nielsen et al. (1982)^27^ | no | Sweden | Prosp. obs. | 100 | - | 100% F | Caesarean section | Ketamine | N₂O/O₂ | - | 1^st^ post-op day | No specific questions reported. Patients were asked about their recall of the operation, dreams, and overall experience | 11% | 0% |
| White (1982)^28^ | no | USA | RCT | 80  (Group 1: 20; Group 2: 20; Group 3: 20; Group 4: 20) | Group 1:  M = 29 ± 2; | Group 1: 50% F, 50% M; | Emergency surgery (general/gynaecologic, orthopaedic) | Group 1: Thiopental | Enflurane (1- 2%) + N₂O/O₂ | - | 1-2h post-op | 1. "Do you recall having a dream like experience during or after your operation?” (no/yes)  2. "If yes, was it unpleasant?" (no/yes/unable to recall) 3. "After you woke up from your operation: (a) did you have trouble separating the dream world from reality? (b) were you afraid? (c) were you worried?” 4. “Since leaving the recovery room have you had any trouble separating the dream world from reality?” | Group 1 (Thiopental): 15% | - |
|  |  |  |  |  | Group 2:  M = 31 ± 2; | Group 2: 25% F, 75% M; |  | Group 2: Ketamine |  |  |  |  | Group 2 (Ketamine): 55% |  |
|  |  |  |  |  | Group 3:  M = 32 ± 2; | Group 3: 20% F, 38% M; |  | Group 3: Midazolam |  |  |  |  | Group 3 (Midazolam): 0% |  |
|  |  |  |  |  | Group 4:  M = 33 ± 3 | Group 4: 45% F, 55% M |  | Group 4: Ketamine + Midazolam |  |  |  |  | Group 4 (Ketamine + Midazolam): 5% |  |
| Carli et al. (1983)^29^ | no | UK | Prosp. obs. | 40 | Range: 19 – 79; Weighted M (based on data provided separately for types of surgeries) = 57.4 | 20% F, 80% M | Thoracic surgery | Etomidate | Etomidate + Fentanyl | - | 24h post-op | No specific questions reported. Patients were asked about recall of unpleasant feelings, incidence of dreams, nausea, last pre-operative memory, and first postoperative memory. | 0% | 0% |
| Klausen et al. (1983)^30^ | no | Denmark | RCT | 40  (Group 1: 20; Group 2: 20) | Range: 23 – 50 | 100% F | Elective gynaecological procedures | Group 1 (Neuroleptanaesthesia): Droperidol + Fentanyl | Group 1 (Neuroleptanaesthesia): Fentanyl (when necessary) + N₂O/O₂ | - | (1) 1^st^ post-op day;  (2) 3 months post-op | - | Group 1 (Neuroleptanaesthesia): 10% | Group 1 (Neuroleptanaesthesia): 20% |
|  |  |  |  |  |  |  |  | Group 2: group: Ketamine | Group 2: Ketamine + N₂O/O₂ |  |  |  | Group 2 (Ketamine): 40% | Group 2 (Ketamine): 5% |
| Cartwright & Pingel (1984)^31^ | yes | UK | RCT | 60d | Group 1: M = 29.3 ± 9.8 | 100% F | Minor gynaecological procedures of less than 15 minutes duration | Group 1: Preinduction with Midazolam, then Ketamine | Additional boluses of Ketamine (if necessary) | - | Within 12h post-op | No specific questions reported. The questionnaire included five questions about the recall of the operation or of dreams, pleasant or unpleasant, the incidence of nausea and vomiting and whether or not she would be happy to receive the same anaesthetic again. | Group 1 (Preinduction with Midazolam): 26.7% | 0% |
|  |  |  |  |  | Group 2: M= 30.1 ± 10.4 |  |  | Group 2: Preinduction with Diazepam, then Ketamine |  |  |  |  | Group 2 (Preinduction with Diazepam): 46.7% |  |
| Mankowitz et al. (1984)^32^ | no | South Africa | RCT | 55  (65 enrolled) | Group 1: M = 25.5 ± 0.9; | 100% F | Elective Caesarean section | Thiopentone | Group 1: N₂O/O₂ + Enflurane (0.6–0.8%) | - | 1^st^ post-op day | No specific questions reported. Participants were "interviewed the next day in an attempt to elicit incidence of factual recall and/or dreaming". | Overall: 14.5%  Group 1: 7.4% | Overall: 10.9% Group 1: 7.4% |
|  |  |  |  |  | Group 2: M = 25.8 ± 0.8 |  |  |  | Group 2: N₂O/O₂ + Ketamine |  |  |  | Group 2: 21.4% | Group 2: 14.3% |
| Sechzer (1984)^33^ | yes | USA | Prosp. obs. | 748 | - | 100% F | Obstetrical delivery | Ketamine  and/or  Thiamylal | N_2_O/O_2_ (in some cases) | - | - | - | Ketamine: 35%  Non-ketamine: 5.6% | - |
| Crawford et al. (1985)^34^ | no | UK | RCT | 777 | - | 100% F | Caesarean section (elective or emergency) | Thiopentone | N₂O/O₂ + Trichloroethylene (0.2 or 0.3 %) OR Halothane (0.2, 0.3, 0.4, or 0.5 %) | - | 1^st^ post-op day | No specific questions reported; Participants were asked if they had had any dreams during the operation. If the answer was affirmative, the possibility of "awareness" was pursued. | Trichloroethylene 0.2%: 4.4%  Trichloroethylene 0.3%: 0%  Halothane 0.2 %: 3.1%  Halothane 0.3 %: 2.3%  Halothane 0.4 %: 0%  Halothane 0.5 %: 0% | Trichloroethylene 0.2%: 5.2%  Trichloroethylene 0.3%: 0.8%  Halothane 0.2%: 7.0%  Halothane 0.3%: 2.3%  Halothane 0.4%: 1.6%  Halothane 0.5%: 0% |
| Iqbal et al (1985)^35^ | no | Bangladesh | Prosp. obs. | 45  (Group 1: 25; Group 2: 20) | Group 1 (male): M = 36.4;  Range: 24 – 60;  Group 2 (female): M = 39.4,  Range: 29 – 62 | 44.4% F, 55.6% M | Group 1: upper abdominal operations;  Group 2: lower abdominal operations | Diazepam + Pethidine + Thiopentone sodium | Incremental Pethidine (every 45 mins) and Gallamine (every 30-40 mins) + inhalational anaesthetics, if needed | - | (1) Immediately post-op;  (2) 4h post-op; (3) 24h post-op | - | 0% | 0% |
| Coad et al. (1986)^36^ | no | UK | RCT | 50  (in 2 groups of 25) | Overall range: 15 – 39  Group 1: M = 22.9 ± 5.7;  Group 2: M = 23.2 ± 5.5 | 100% F | Suction termination of pregnancy | Group 1: Methohexitone  Group 2: Ketamine | N₂O/O₂ + incremental doses of Methohexitone or Ketamine, respectively | - | Unclear (late post-op) | No specific questions reported. Participants were asked about recall of events leading up to and during surgery, and about dreaming. | 0% | - |
| Schultetus et al. (1986)^37^ | yes | USA | RCT | 36 | Group 1: M = 23.5 ± 5.3; | 100% F | Elective Caesarean section | Group 1: Ketamine (1mg/kg) | N₂O/O₂ | - | (1) After patients had awakened fully;  (2) 1^st^ post-op day | No specific questions reported. Patients were asked if they remembered anything from during their operation. | Overall: 11%  Group 1 (Ketamine): 0%; | 8.3% |
|  |  |  |  |  | Group 2: M = 23.5 ± 4.5; |  |  | Group 2: Thiopental |  |  |  |  | Group 2 (Thiopental): 15.38%; |  |
|  |  |  |  |  | Group 3: M = 25.5 ± 5.2 |  |  | Group 3: Ketamine + Thiopental |  |  |  |  | Group 3 (Ketamine + Thiopental): 18% |  |
| de Grood et al. (1987)^38^ | no | Netherlands | RCT | 30  (in 2 groups) | Overall range: 20 – 79;  Group 1: M = 55.5 ± 13.2; | - | Micro-laryngeal surgery | Group 1: Propofol, | Group 1: Propofol | - | - | - | Group 1 (Propofol): 6.7% | 0% |
|  |  |  |  |  | Group 2: 54.9 ± 12.7 |  |  | Group 2: Etomidate | Group 2: Etomidate |  |  |  | Group 2 (Etomidate): 0% |  |
| Moore & Seymour (1987)^39^ | no | UK | RCT (Double-Blind) | 104 | Group 1 (noise group): M = 63.6 ± 1.3; Range: 39 – 81 | Group 1: 14% F, 86% M; | Elective bronchoscopy | Thiopentone | Thiopentone (as needed) | - | 6h post-op | Modified Brice interview (Brice et al., 1970): 1. “What was the last thing you remember before going to sleep?” 2. “What was the next thing you remember after that?” 3. “Do you remember having any dreams while you were asleep?” | Overall: 3.8%  Group 1 (noise): 0% | Overall: 6.8%  Group 1 (noise): 5.9% |
|  |  |  |  |  | Group 2 (no noise group): M = 61.2 ± 1.7;  Range: 15 – 77 | Group 2: 13% F, 87% M |  |  |  |  |  |  | Group 2 (no noise): 7.7% | Group 2 (no noise): 7.7% |
| Abu Khalaf et al. (1988)^40^ | no | Jordan | Prosp. obs. | 12 | M = 36.8; Range: 11 – 61 | 25% F; 75% M | Open liver biopsy | Ketamine | Ketamine (as necessary) | - | 1^st^ post-op day | - | 50% | Not directly mentioned but seems that 0% |
| Galletly & Short (1988)^41^ | no | New Zealand | Prosp. obs. | 50 | M = 51; Range: 13 – 80 | 46% F, 54% M | Elective surgery, mostly upper gastrointestinal, but also craniotomy, spinal surgery, vascular, gynaecological | Fentanyl + Propofol | Propofol | - | During recovery | No specific questions reported. Patients were asked about intraoperative dreaming and awareness. | 6% | 0% |
| Ghaly et al. (1988)^42^ | no | UK | Prosp. obs. | 50  (Group 1: 25, Group 2: 25) | Isoflurane series:  M = 28 ± 6  Halothane series:  M = 28 ± 4) | 100% F | Elective Caesarean section | Thiopentone | Halothane (0.5%) or Isoflurane (0.75%) + N₂O/O₂ | - | 1^st^ post-op day | No specific questions reported. Patients were asked about intraoperative dreaming and awareness. | 0% | 0% |
| Gunawardene (1989)^43^ | yes | UK | Unclear | 50 | - | 100% F | Minor gynaecological procedures | Propofol + Enflurane + N₂O/O | | - | - | - | 24% | - |
| Bogod et al (1990)^44^ | yes | Hong Kong | Prosp. obs. | 74 | - | 100% F | Caesarean section (emergency and elective) | Thiopentone | Enflurane (1%) + N₂O/O₂ | - | ~24h post-op | No specific questions reported. Patients were asked a series of questions about dreaming, awareness and pain during the period of anaesthesia. | 12%  (elective: 7%; emergency: 28%) | 2.7% |
| Eger et al. (1990)^45^ | no | USA | RCT (Double-Blind) | 270 | N₂O group: M = 54 ±18 | N₂O group: 74.4% F,  25.6% M; | Elective total hip arthroplasty, carotid endarterectomy, or transsphenoidal hypophysectomy. | Thiopental | Isoflurane, with or without N₂O/O₂ or O₂ | - | 90 min post-op | 1. "Did you dream during anaesthesia?"  2. "Do you remember events during anaesthesia?" | N₂O group: 5.3% | N₂O group: 0.8% |
|  |  |  |  |  | No N₂O group: M = 54 ± 17 | No N₂O group: 78.1% F,  21.9% M |  |  |  |  |  |  | No N₂O group: 8.8% | No N₂O group: 4.5% |
| Piggott et al. (1990)^46^ | no | UK | RCT | 200  (O₂ group: 103,  N₂O/O₂ group: 97) | - | 100% F | Caesarean sections (elective and emergency) | Thiopentone | Isoflurane in 100% O₂ or in 50% N₂O/O₂ | - | 24h post-op | - | 2% | 0% |
| Restall et al. (1990)^47^ | no | UK | RCT (Double-Blind) | 50 | Overall range: 16 – 60  Group 1: M = 35.9 ± 7.7 | 100% F | Gynaecological surgeries | Midazolam + Ketamine + Alfentanil | Group 1: Ketamine + Midazolam + Alfentanil | - | Upon return to ward | No specific questions reported. Patients were asked whether they had had any dreams and, if so, whether they were pleasant or not; whether the anaesthetic regimen was to their satisfaction and whether they felt sick and (or) vomited at any time after their anaesthetic. | Group 1 (Placebo): 8% | - |
|  |  |  |  |  | Group 2: 34.8 ± 8.9 |  |  |  | Group 2: Ketamine + Midazolam + Alfentanil + Flumazenil post-anaesthesia |  |  |  | Group 2 (Flumazenil): 52% |  |
| Guit et al. (1991)^48^ | no | Netherlands | RCT (Double-Blind) | 18  (Group 1: 9 Group 2: 9) | Group 1: M = 35 ± 12  Group 2: M = 39 ± 12 | 72% F, 28% M | Non-cardiac surgery (abdominal, gynaecological, orthopaedic, plastic, ear, nose and throat) | Group 1: Propofol (TIVA)+ Ketamine  Group 2: Propofol (TIVA) + Fentanyl | Propofol (TIVA) | - | 1^st^ post-op day | - | 0% | - |
| Liu et al. (1991)^49^ | yes | UK | Prosp. obs. | 1000  (some patients excluded from post-operation interview) | Range: 16 – >85 | ~52% F, ~48% M | General: n = 324  Gynaecological: n = 298  Orthopaedic: n = 160  Ear, Nose, and Throat: n = 113  Ophthalmological: n = 97  Other: n = 8 | Those 9 patients who dreamed received Thiopentone (n = 6)  or  Propofol (n = 2),  or Etomidate (n = 1) | - | - | 20 - 36h post-op | Modified Brice interview (Brice et al., 1970): 1. “What was the last thing you remember before you went to sleep for your operation?” 2. “What was the first thing you remember after your operation?” 3. “Can you remember anything in-between these two periods?” 4. “Did you dream during your operation? 5. “What was the worst” thing about your operation?” | 0.9% | 0.2% |
| Lyons & Macdonald (1991)^50^ | no | UK | Retrosp. obs. | 3076 | - | 100% F | Caesarean section | Thiopentone | Up to year 1985: Halothane (0.5%) + N₂O/O₂ | - | Post-op | 1. “Do you remember anything about your operation?"  2. “Did you dream during your operation?”  And, if appropriate, the content of the dream was reported. | Overall: 6.1%  Up to year 1985: 7.4% | Overall: 0.9%  Up to year 1985: 1.3% |
|  |  |  |  |  |  |  |  |  | Since 1985: Isoflurane (1%) + N₂O/O₂ |  |  |  | Since 1985: 4.7% | Since 1985: 0.4% |
|  |  |  |  |  |  |  |  |  |  |  |  |  |  |  |
| Marsch et al. (1992)^51^ | yes | Switzerland | RCT | 60 (Group 1: 30;  Group 2: 29, 1 dropout) | Group 1: M = 38 ± 16; | 42% F, 58% M | Elective ear, nose, and throat surgery | Group 1: Propofol + Fentanyl | Group 1: Propofol + Fentanyl (as needed) + oxygen enriched air (40% oxygen) | - | (1) Immediately after extubation;  (2) In the Recovery room;  (3) In the evening of the day of the surgery | "Did you dream or have any other experiences while asleep?" If yes, further asked to specify whether the experience was "very pleasant", "pleasant", "unpleasant", "very unpleasant", or "unspecific" | Group 1:  Recall upon extubation: 43%  Recall in recovery room: 10% | 0% |
|  |  |  |  |  | Group 2: M = 36 ± 13 |  |  | Group 2: Thiopentone + Fentanyl | Group 2: N_2_O/Enflurane/Oxygen (40%) + Fentanyl (as needed) |  |  |  | Group 2:  Recall upon extubation: 10%  Recall in recovery room:  3% |  |
| Jakobsson et al. (1993)^52^ | no | Sweden | RCT | 200 | Range: 18 – 45 | 100% F | Termination of pregnancy (dilatation and vacuum curettage of the uterine cavity) | Group 1: Propofol + Ketamine | N₂O/O₂ + additional doses of Propofol, Methohexitone, or Thiopentone, if necessary | - | At the time of discharge | No specific questions reported. The questionnaire included questions about memories of the operation, dreams during anaesthesia, pain, emesis and experiences during the recovery period. | Group 1 (Propofol + Ketamine): 58% | 0% |
|  |  |  |  |  |  |  |  | Group 2: Propofol + Fentanyl |  |  |  |  | Group 2 (Propofol + Fentanyl): 22% |  |
|  |  |  |  |  |  |  |  | Group 3: Thiopentone + Fentanyl |  |  |  |  | Group 3 (Thiopentone + Fentanyl): 14% |  |
|  |  |  |  |  |  |  |  | Group 4: Methohexitone + Fentanyl |  |  |  |  | Group 4 (Methohexitone + Fentanyl): 8% |  |
| Oddby-Muhrbeck & Jakobsson (1993)^53^ | no | Sweden | RCT | 60  (in 2 groups of 30) | Overall: M = 31 ± 7  Group 1:  M = 32 ± 7 | 100% F | Elective laparoscopic surgery for infertility, abdominal pain, or sterilization | Propofol TIVA | Group 1: Propofol TIVA + N₂O/O₂ | - | 24h post-op | 1. “Do you remember anything from the operation?” 2. “Did you have any good experience from the operation?” 3. “Did you have any bad experience from the operation?” 4. “Did you have any dreams during the operation?” 5. “Do you think you have heard any music?” (after this patients were played 7 pieces of music and asked if they were exposed to any of these during the operation) | Overall: 6.7%  Group 1 (Propofol): 10% | 0% |
|  |  |  |  |  | Group 2:  M = 30 ± 6. |  |  |  | Group 2: Isoflurane (1.2%) + N₂O/O₂ |  |  |  | Group 2 (Isoflurane): 3.3% |  |
| Krissel et al. (1994)^54^ | no | Germany | RCT | 75  (in 3 groups of 25) | Group 1: M = 29.7, SEM = 0.9 | 100% F | Caesarean section (elective) | Group 1: Thiopentone | Group 1:  Before delivery: N₂O/O₂ + Enflurane (0.8%)  After delivery: N₂O/O₂+ Enflurane (1–1.5%) | - | 24h post-op | No specific questions reported. Patients were asked about possible anaesthesia-related side-effects, episodes of consciousness during the operation and dreams occurring during the procedure or in the recovery phase. | Overall: 5.3%  Group 1 (Thiopentone): 0% | 1.3% |
|  |  |  |  |  | Group 2: M = 30.3, SEM = 0.8 |  |  | Group 2: Thiopentone + Ketamine | Group 2: Before delivery: N₂O/O₂ + Enflurane (0.8%)  After delivery: N₂O/O₂+ Enflurane (1–1.5%) |  |  |  | Group 2 (Thiopentone + Ketamine): 4% |  |
|  |  |  |  |  | Group 3: M = 30.4, SEM = 0.9 |  |  | Group 3: Ketamine | Group 3: Before delivery: N₂O/O₂  After delivery: N₂O/O₂+ Enflurane (1–1.5%) |  |  |  | Group 3 (Ketamine): 25% |  |
| Liou et al. (1994)^55^ | yes | Taiwan | Prosp. obs. | 100  (in 2 groups of 50) | Group 1: M = 43.52 ± 10.88  Group 2: M = 44.56 ± 7.51 | Overall: 95% F, 5% M;  Group 1: 94% F, 6% M;  Group 2: 96% F, 4% M | Intraabdominal surgeries (Gynaecology, Urology, General Surgery, CRS) | Group 1 (GA): Propofol or Thiopentone  Group 2 (GA + epidural): Induction followed the same regimen as in group 1 | N₂O/O₂+ Isoflurane | - | 20-36h post-op | First, patients were asked whether they had awakened with recall or heard anything or dreamed intraoperatively. If yes, then Modified Brice interview (Brice et al., 1970): 1. “What was the last thing you remember before you went to sleep for your operation?” 2. “What was the first thing you remember after your operation?” 3. “Can you remember anything between these two periods?” 4. “Did you dream during your operation?” 5 “What was the worst thing about your operation?” | 0% | 0% |
| Oxorn et al. (1994)^56^ | yes | Canada | RCT (Double-Blind) | 56 (Group 1: 29; Group 2: 27) | Group 1: M = 45.6 ± 11.7 | 100% F | Outpatient diagnostic dilation and curettage | Group 1: Propofol | Group 1: Propofol + N₂O/O₂ | - | (1) 1h post-op; (2) 24h post-op | - | Group 1:  1h postop: 20.7%  24h postop: 17.2% | 0% |
|  |  |  |  |  | Group 2: M = 44.0 ± 10.0 |  |  | Group 2: Thiopental | Group 2: Thiopental: + N₂O/O₂ |  |  |  | Group 2:  1h postop: 14.8%  24h postop:  18.5% |  |
| Suárez et al. (1994)^57^ | no | Mexico | RCT | 400 (Group 1: 149; Group 2: 251) | M = 46 ± 18 | 50.2% F, 49.8% M | Different surgical procedures (neurosurgery, ophthalmology, gastrosurgery, ear/nose/throat, neck surgery, urology, others) | Propofol + Fentanyl | Continuous infusion of Propofol + Fentanyl i/v bolus:  Group 1: Propofol infusion rate < 6 mg/kg/h  Group 2: Propofol infusion rate > 6mg/kg/h | - | 8-24h post-op | Modified Brice interview (Brice et al., 1970): 1. “What is the last thing you remember before falling asleep?” 2. “What is the first thing you remember when you woke up?” 3. “Do you remember anything between these two periods?” 4. “Do you remember having had any dreams between these two periods?” | Group 1 (infusion < 6 mg/kg/h): 11.4% | Group 1 (infusion < 6 mg/kg/h): 17.4%; |
|  |  |  |  |  |  |  |  |  |  |  |  |  | Group 2 (infusion > 6 mg/kg/h): 2% | Group 2 (infusion > 6 mg/kg/h): 0.8% |
| Dutton et al. (1995)^58^ | no | USA | Prosp. intv. | 326 | Range: 18 – 42 | 100% F | Pelvic laparoscopy | Thiopental | Isoflurane + N_2_O  or  Isoflurane + N_2_O + Fentanyl  or  N_2_O + Fentanyl  or  Isoflurane + Fentanyl | - | After recovery from anaesthesia, but prior to discharge | No specific questions reported. Patients were asked if they recalled hearing or experiencing anything during surgery or having unpleasant dreams. | 0% | 0% |
| Adesunkanmi (1997)^59^ | no | Nigeria | Prosp. obs. | 282 | Range: 4 – 75 | 37.2% F, 62.8% M | Major and minor surgical procedures. (57.4% elective and 42.6% emergency procedures) | Ketamine (72% procedures); Thiopentone (3.9% procedures); Spinal (11.3% procedures); Local (12.8%) | Ketamine or Thiopentone | - | - | - | Ketamine: 5.4% (not reported for others) | Not directly mentioned but seems that 0% |
| Brandner et al (1997)^60^ | yes | UK | RCT | 112 | Group 1: M = 45.9 ± 11.5 | 62.5% F, 37.5% M | Day-case varicose vein surgery | Group 1: Propofol (TIVA) | Group 1: Propofol | - | When patients were orientated in time, place, and person | No specific questions reported. Patients were asked if they had any dreams or images during the anaesthetic or thought they had had a dream but were unable to recall the content. The patients were then asked whether they had any of the six feelings: angry, apprehensive, happy, sad, confused, sexual. They were also asked whether they experienced any of the six bodily states: sick, hungry, high, sedated, dizzy, light-hearted, headache. | Overall: Dreams with Recall: 21.4% Dreams without Recall: 14.3%  Group 1 (Propofol TIVA): Dreams with Recall: 27.8% Dreams without Recall: 27.8% | - |
|  |  |  |  |  | Group 2: M = 44.6 ± 9.1 |  |  | Group 2: Propofol (induction only) | Group 2: N₂O/O₂+ Isoflurane (1-1.5%) |  |  |  | Group 2 (Propofol induction): Dreams with Recall: 18.9% Dreams without Recall: 13.5% |  |
|  |  |  |  |  | Group 3: M = 44.7 ± 11.6 |  |  | Group 3: Thiopentone | Group 3: N₂O/O₂+ Isoflurane (1-1.5%) |  |  |  | Group 3 (Thiopentone):  Dreams with Recall: 17.9% Dreams without Recall: 2.6% |  |
| da Silva et al. (1997)^61^ | no | UK | RCT (Double-Blind) | 60  (in 3 groups of 20) | Group 1: M = 43.6; Range 21 – 65  Group 2: M = 45.4; Range 25 –63  Group 3: M = 45.4; Range 20 –65 | Group 1: 70% F, 30% M;  Group 2: 70% F, 30% M;  Group 3: 50% F, 50% M | Upper or lower abdominal or superficial surgery | Etomidate | Group 1: Halothane    Group 2: Enflurane  Group 3: Isoflurane | - | 1^st^ post-op day | Modified Brice interview (Brice et al., 1970):  (1) “What is the last thing you remember before going to sleep?” (2) “What is the first thing you remember after waking up?” (3) “Did you have any dreams? (if yes, what were they)” (4) “Would you accept the same type of anaesthetic again?” | 0% | - |
| Kee et al. (1997)^62^ | no | Hong Kong | RCT (Double-Blind) | 38  (40 recruited; Group 1: 18; Group 2: 20) | Group 1: M = 32.5; Range 29–34  Group 2: M = 31; Range 29–34 | 100% F | Elective Caesarean section | Group 1: Ketamine  Group 2: Thiopental | N₂O/O₂+ Isoflurane (0.5%) | - | 24h post-op | No specific questions reported. Patients were asked whether they had any recall of intraoperative events and whether they had experienced pleasant or unpleasant intraoperative dreams. | Overall: 7.9%  Group 1 (Ketamine): 5.6% | 0% |
|  |  |  |  |  |  |  |  |  |  |  |  |  | Group 2 (Thiopental): 10% |  |
| Nordström et al. (1997)^63^ | no | Sweden | Prosp. obs. | 1000 (second interview) | M = 42 ± 15; Range: 16 – 89 | 71.8% F, 28.2% M | Different types of surgeries (gynaecological, ENT, ophthalmological, dental, general surgery, orthopaedic) | Alfentanil + Propofol (TIVA) | Propofol (TIVA) + Alfentanil | - | (1) At PACU discharge;  (2) 1^st^ post-op day;  (3) 1 week post-op | Modified Brice interview (Brice et al., 1970): 1. “What was the last thing you remembered before you went to sleep?” 2. “What was the first thing you remembered after your operation?” 3. “Can you remember anything in between?” 4. “Can you remember if you had any dreams during your operation?” 5. “What was the worst thing about your operation?” | After the 2^nd^ interview:  2.7%  After the 3^rd^ interview (includes patients who had not previously reported dreaming): additional 2.1% | 0.2% |
| Oxorn et al. (1997)^64^ | yes | Canada | RCT (Double-Blind) | 60 | Group 1: M = 49.4 ± 2.2 | 100% F | Outpatient diagnostic dilatation and curettage with or without hysteroscopy | Group 1: (Pre-induction Midazolam) + Propofol | Propofol (boluses) + N₂O/O₂ | - | (1) immediately upon emergence;  (2) 30 min post-op in PACU;  (3) 1^st^ post-op day (by telephone) | - | Group 1:  Immediately upon emergence: 27%;  In PACU: 12%;  1^st^ post-op day: 13% | - |
|  |  |  |  |  | Group 2: M = 48.9 ± 2.2 |  |  | Group 2: (Pre-induction Placebo) + Propofol |  |  |  |  | Group 2:  Immediately upon emergence: 22%  In PACU: 12%;  1^st^ post-op day: 12% |  |
| Ranta et al. (1998)^65^ | no | Finland | Prosp. obs. | 2612  (4818 enrolled) | > 12; Dreamers: 41 ± 15; Non-dreamers: 45 ± 17 | 77% F, 23% M | Different elective surgeries | Thiopental or Propofol | N₂O/O₂+ volatile anaesthetic (Isoflurane or Enflurane) + opioids (Fentanyl or alfentanil) | - | Immediately before PACU discharge | Brice interview (Brice et al., 1970) but no specific questions reported. Patients were also asked to rate their dreams as pleasant, neutral, or unpleasant. | 13.1% | 0.4% |
| Gray et al. (1999)^66^ | no | UK | Prosp. obs. | 19 | M = 61; Range: 18 – 80 | 31.6% F, 68.4% M | Different surgeries | Ketamine TCI + Propofol TIVA | Propofol TIVA + Ketamine TCI | - | 1^st^ post-op day | No specific questions reported. Patients were asked about the occurrence of vivid dreams or hallucinations. | 0% | 0% |
| Kudoh et al. (1999)^67^ | no | Japan | RCT | 40  (in 2 groups of 20) | Overall range: 20 – 30  Group 1: M = 25.6 ± 0.8; | - | Reduction of lower and upper limb fractures | Group 1 and 2: Ketamine + Fentanyl | Group 1: Ketamine (2 mg/kg/h) + Fentanyl (5μg/kg/h for the first 60min and 3 5μg/kg/h for the next 90min) | - | Unclear but postop | 1. "Do you remember anything in between? 2. "Did you have any dreams or have any other experiences while you were asleep?” | Group 1: 25% | 0% |
|  |  |  |  |  | Group 2: M = 24.8 ± 0.7 |  |  |  | Group 2: Ketamine (2 mg/kg/h) + Fentanyl (50-100 μg intermittently) |  |  |  | Group 2: 50% |  |
| Yeh et al. (1999)^68^ | no | Taiwan | RCT | 60 | Overall range: 16 – 64 years  Group 1: M = 40.3 ± 3.3 | 33% F, 67% M | Various procedures that could be facilitated by spinal anaesthesia (orthopaedic, urologic, general, plastic) | Group 1: Ketamine (0.5 mg/kg) + Midazolam (0.05 mg/kg) | None | - | In PACU when patients were fully awake | - | Group 1: 23.3% | 0% |
|  |  |  |  |  | Group 2: M = 41.6 ± 3.7 |  |  | Group 2: Ketamine (0.5 mg/kg) + Midazolam (0.1 mg/kg) |  |  |  |  | Group 2: 13.3 % |  |
| Chudnofsky et al. (2000)^69^ | no | USA | Prosp. obs. | 70  (77 enrolled) | M =31;  Range: 18 – 68 | 59% F, 41% M | Various procedures (abscess incision and drainage, fracture/joint reduction, other procedures) | Midazolam + Ketamine | - | - | Prior to discharge (not specified) | No specific questions reported. Patients were asked if they experienced any dreams while sedated, and if so, whether these were pleasant, unpleasant, both, or neither. | 25% | - |
| Ghoneim et al. (2000)^70^ | no | USA | RCT | 180  (Group 1: 100; Group 2: 40; Group 3: 16; Group 4: 24) | Overall range: 18 – 50  Group 1: M = 34.2 ± 0.9 | 72% F, 28% M | Elective surgeries | Group 1: Fentanyl + Thiopental | Group 1: N₂O/O₂+ Fentanyl boluses (as needed) | - | (1) The first 24 patients: 1^st^ post-op day;  (2) The next 72 patients: 3–4 days post-op in the hospital or by telephone;  (3) The last 84 patients: 1^st^ post-op day in the hospital | No specific questions reported but modified Brice interview (Brice et al., 1970) was used:  Patients were asked about the last thing they remembered before going to sleep; the first thing they remembered when they woke up and anything in between, including sounds, dreams or fantasies. | Overall: 6.7%  Group 1 (Fentanyl bolus): 8% | 3.4% |
|  |  |  |  |  | Group 2: M = 33.7 ± 1.4 |  |  | Group 2: Alfentanil +Thiopental | Group 2: Alfentanil infusion + N₂O/O₂ |  |  |  | Group 2 (Alfentanil infusion): 5% |  |
|  |  |  |  |  | Group 3: M = 34.3 ± 2.1 |  |  | Group 3: Fentanyl + Thiopental | Group 3: N₂O/O₂+ Isoflurane ( 0.3%)+ Fentanyl boluses (as needed) |  |  |  | Group 3 (Isoflurane 0.3%): 6.3% |  |
|  |  |  |  |  | Group 4: M = 31.6 ± 1.9 |  |  | Group 4: Fentanyl + Thiopental | (4) Group 4: N₂O/O₂+ Isoflurane (0.7%) |  |  |  | Group 4 (Isoflurane 0.7%): 4.2% |  |
| Grace et al. (2001)^71^ | no | Vanuatu | RCT | 45  (Group 1: 21, Group 2: 24) | Group 1: M = 33.9 ± 4.9 | 100% F | Post-partum tubal ligation | Group 1: Ketamine (+ Diazepam) | Ketamine | - | (1) 1hr post-op  (2) 4hr post-op | No specific questions reported. Patients were asked if they had dreamed and to rate the dream content on a scale 1 to 5 (from 1 = very unpleasant to 5 = very pleasant) | Group 1 (Ketamine + Diazepam): 52% | - |
|  |  |  |  |  | Group 2: M = 34.7 ± 6.1 |  |  | Group 2: Ketamine (+ Lignocaine) |  |  |  |  | Group 2 (Ketamine + Lignocaine): 54% |  |
| Aceto et al. (2002)^72^ | yes | Italy | RCT | 32  (in 4 groups of 8) | Range: 18 – 70 | - | Elective laparoscopic cholecystectomy | Thiopental sodium + Fentanyl | Group 1: Sevoflurane + O_2 (_40%)  Group 2: Sevoflurane + N₂O/O₂  Group 3: Isoflurane + O_2 (_40%)  Group 4: Isoflurane + + N₂O/O₂ | - | 24h post-op | No specific questions reported but modified Brice interview (Brice et al., 1970) was used: last thing they remember before going to sleep, first thing they remember when they woke up, anything which happened in between, including sounds, dreams and imagination. | 3.1% | 0% |
| Chaiwat et al. (2002)^73^ | no | Thailand | Prosp. obs. | 802 | M = 47.25 ± 13.12); Range: 16 – 84 | 82% F, 18% M | Elective general (abdomen, thyroid/breast) and gynaecological) | Thiopental: n = 798  Ketamine: n = 4 | Combination of agents including N_2_O and volatile anaesthetics:  Volatiles: None: n = 1;  Halothane: n = 675;  Isoflurane: n = 124;  Sevoflurane: n = 2  N_2_O: None: n = 11;  less than  50%: n = 13;  50-60%: n = 138;  61-70%: n = 640 | - | (1) 12-15h post-op;  (2) 3^rd^ post-op day, if awareness was suspected | Modified Brice interview (Brice et al., 1970): 1. “What was the last thing you remembered before sleeping?” 2. “What was the first thing you remembered after you regained consciousness?” 3. “Did you dream during anaesthesia?” 4. “Did you recall anything during anaesthesia?” 5. “Did you feel anything during surgery?” 6. “Did you try to tell someone about your awakening?” 7. “Did you fear or worry before operation?” 8. “Have you ever been previously operated or under general anaesthesia?” 9. “What was the worst feeling during general anaesthesia?” | 12% | 0.6% |
| Vallejo et al. (2002)^74^ | no | USA | RCT | 119  (Group 1: 59, Group 2: 60) | Group 1: M = 34.1 ± 5.9; | 100% F | Elective outpatient laparoscopic tubal ligation | Group 1. Propofol + Ketamine | Isoflurane (0.5%–1.5%) + N₂O/O₂ | - | on PACU admission | No specific questions reported. Patients were asked whether they remembered dreaming, and they were encouraged to describe their experience as pleasant or unpleasant. | Group 1 (Propofol + Ketamine): 16.9% | - |
|  |  |  |  |  | Group 2: M = 34.3 ± 6.1 |  |  | Group 2: Propofol + Fentanyl |  |  |  |  | Group 2 (Propofol + Fentanyl): 1.7% |  |
| Yeo & Lo (2002)^75^ | no | Singapore | Prosp. obs. | 20 | M = 31 ± 5.7; Range: 25.3 – 36.7 | 100% F | Elective Caesarean section | Thiopentone | Isoflurane + N₂O/O₂ | Yes (BIS; Mdn ≤ 70 on most occasions during surgery; range: 52-70) | 1^st^ post-op day | No specific questions reported. Patients were asked about the experience of dreaming or recall. | 0% | 0% |
| Aceto et al. (2003)^76^ | no | Italy | RCT | 40  (in 4 groups of 10) | Overall range: 39 – 59  Group 1: M = 54; Range: 50 – 58 | 52% F, 48% M | Elective laparoscopic cholecystectomy | Thiopental sodium + Fentanyl | Group 1: Sevoflurane + O_2 (_40%) | - | 24h post-op | No specific questions reported but modified Brice interview (Brice et al., 1970) was used: Patients were asked about the last thing they remembered before going to sleep; the first thing they remembered when they woke up; and anything  which happened in between, including sounds, dreams, and  imagination. | Overall: 2.5%  Group 1: 0% | 0% |
|  |  |  |  |  | Group 2: M = 49; Range: 39 – 59 |  |  |  | Group 2: Sevoflurane + N₂O/O₂ |  |  |  | Group 2: 2.5% |  |
|  |  |  |  |  | Group 3: M = 52; Range: 47 – 57 |  |  |  | Group 3: Isoflurane + O_2 (_40%) |  |  |  | Group 3: 0% |  |
|  |  |  |  |  | Group 4: M = 50; Range: 43 – 57 |  |  |  | Group 4: Isoflurane + + N₂O/O₂ |  |  |  | Group 4: 0% |  |
| Grace (2003)^77^ | yes | Vanuatu | Field RCT (Double-Blind) | 400 | Range: 18 – 60 | 61% F, 39% M | Minor procedures (e.g., manipulation of fractures, incision and drainage, dilatation and curettage) | (All groups received Fentanyl, followed by 0 mg/kg/min Diazepam, 0.025 mg/kg/min Diazepam, 0.05 mg/kg/min Diazepam, 0.1 mg/kg/min Diazepam, or 0.175 mg/kg/min Diazepam)  + Ketamine (infusion) | - | - | Before discharge from recovery | No specific questions reported. Patients were asked whether they had dreamed and, if so, to rate their dreams on a scale from 1 = very unpleasant to 5 = very pleasant. | 0 mg/kg Diazepam: 94.6%  0.025 mg/kg Diazepam: 85%  0.05 mg/kg Diazepam: 84.6%  0.1 mg/kg Diazepam: 67.5%  0.175 mg/kg Diazepam: 73.8% | - |
| Hellwagner et al. (2003)^78^ | yes | Austria | RCT | 50  (in 2 groups of 25) | Overall range: 20 – 60;  Group 1: M = 46 ± 7) | 100% F | Elective placement of applicators for brachytherapy for breast cancer | Group 1: Propofol + Fentanyl | Group 1: Propofol | Yes (EEG) | (1) Immediately post-anaesthesia  (2) 10 min post-awakening  (3) 30 min post-awakening  (4) 3h post-awakening  (5) 3 months post- awakening | No specific questions reported. The standardized questionnaire asked patients to recall whether there were any erotic aspects in their dreams. | Group 1:  Immediately post-anaesthesia: 40%;  10min post-awakening: 40%;  30min post-awakening: 40%;  3h post-awakening:  40%;  3m post-awakening: 36%; | 0% |
|  |  |  |  |  | Group 2: M = 40 ± 5 |  |  | Group 2: Methohexital-Isoflurane + Fentanyl | Group 2: Isoflurane (1%) |  |  |  | Group 2:  Immediately post-anaesthesia: 24%;  10min post-awakening: 24%;  30min post-awakening: 16%;  3h post-awakening: 16%;  3m post-awakening: 8% |  |
| Iwama et al. (2003)^79^ | no | Japan | Prosp. obs. | 262 (enrolled 265) | M = 47 ± 2; Range: 18 – 79 | 80.8 % F,  19.2% M | Lower extremity or lower abdominal gynaecological surgery | Propofol | Propofol | - | (1) Immediately post-op;  (2) 1^st^ post-op day | - | 54% | 0% |
| Kudoh et al. (2004)^80^ | no | Japan | RCT | 150  (2 groups of 75) | Group 1: M = 75.9 ± 4.0 | Group 1: 92% F, 8% M; | Total knee arthroplasty | Propofol | Group 1: Propofol | - | (1) Immediately after extubation or LMA removal;  (2) 1h later | 1. "Did you dream while asleep?”  2. “Was the dream pleasant, unpleasant, or unspecific?" | Group 1: 64% | - |
|  |  |  |  |  | Group 2: M = 75.1 ± 4.2 | Group 2: 88% F, 12% M |  |  | Group 2: Propofol + Fentanyl (given until skin incision) |  |  |  | Group 2: 13% |  |
| Kwok et al. (2004)^81^ | no | Hong Kong | RCT (Double-Blind) | 135  (3 groups of 45) | Overall range: 18 – 65;  Group 1: M = 34 ± 7 | 100% F | Laparoscopic gynaecologic surgery | Group 1: Propofol and Fentanyl + Ketamine before incision | N₂O/O₂ + Isoflurane (0.5%–1%) | - | Patients were interviewed frequently during the first postoperative day and then daily until hospital discharge (unclear when they were asked about dreaming) | - | Overall: 2.2%  Group 1: 0% | 0% |
|  |  |  |  |  | Group 2: M = 32 ± 5 |  |  | Group 2: Propofol and Fentanyl + Ketamine after wound closure |  |  |  |  | Group 2: 2.2% |  |
|  |  |  |  |  | Group 3: M = 34 ± 6 |  |  | Group 3: Propofol and Fentanyl; no Ketamine) |  |  |  |  | Group 3: 4.4% |  |
| Myles et al. (2004)^82^ | no | Australia | RCT (Double-Blind) | 2463  (BIS group: 1225; No-BIS group: 1238) | BIS group:  M = 58.1 ± 16.5 | BIS group: 39% F, 61% M; | High-risk procedures, including cardiac surgery, trauma, caesarean section, bronchoscopy, and laryngoscopy | Different combinations of:  Propofol, Thiopentone, or Midazolam | Propofol (TIVA) or N_2_O and/or Volatile anaesthetics and/or Fentanyl | Yes  (BIS group: BIS target range was 40–60 (time-averaged throughout the procedure was 44.5 ± 6.8, lowest 33.6, and highest 55.5; No-BIS group: BIS was attached but not turned on) | (1) 2–6h post-op;  (2) 24–36h post-op;  (3) 30 days post-op | No specific questions reported. A structured questionnaire was used. | BIS group: 5.2% | BIS group:  0.2% |
|  |  |  |  |  | No-BIS group:  M = 57.5 ± 16.9 | No-BIS group: 37% F, 63% M |  |  |  |  |  |  | No-BIS group: 6.9% | No-BIS group: 0.9% |
| Sebel et al. (2004)^83^ | no | USA | Prosp. obs. | 19575 | ≥18  Awareness (n = 25): M = 52 ± 15;    Possible awareness (n = 46): M = 48 ± 14;  No awareness (n = 19504): M = 48 ± 16 | 56.8% F, 43.2% M | Different surgeries (abdominal/thoracic, cardiac, orthopaedic, other) | - | - | Yes (BIS) | (1) In PACU;  (2) 1-2 weeks post-op | Modified Brice interview (Brice et al., 1970): 1. “What is the last thing you remember before going to sleep?” 2. “What is the first thing you remember waking up?” 3. “Do you remember anything between going to sleep and waking up?” 4. “Did you dream during your procedure?” 5. “What was the worst thing about your operation?” | (1) In PACU: 6%  (2) 1-2 weeks post-op: 3.4% | 0.1% |
| Wanna et al. (2004)^84^ | no | Thailand | RCT (Double-Blind) | 100  (2 groups of 50) | Overall range: 21 – 42  Group 1: M = Propofol: 28.54 ± 5.89;  Group 2: M = 27.16 ± 4.73 | 100% F | Elective Caesarean section | Group 1: Propofol  Group 2: Ketamine | Sevoflurane + N₂O/O₂ | - | 24h post-op | - | Unclear (it is only said: "In the propofol group 8% had a good dream showing a higher incidence than the ketamine group" but no data presented for ketamine group. Unclear what is the overall recall %) | 0% |
| Leslie et al. (2005)^85^ | yes | Australia | RCT (Double-Blind) | 2385 | Dreamers: M = 52; Range 37 – 68  Non-dreamers: M = 61; Range 46 – 71 | 38% F, 62% M | High-risk procedures, including cardiac surgery, trauma, Caesarean section, bronchoscopy, laryngoscopy | Anaesthetics were used according to the anaesthetists’ discretion:  Propofol, Thiopental | Anaesthetics were used according to the anaesthetists’ discretion:  Propofol (TIVA), Inhalational agents (not specified), N_2_O, Fentanyl | Yes  (BIS-group: 40-60 during surgical procedure)  No (No-BIS group: no BIS used) | (1) 2-4h post-op;  (2) 24–36h post-op;  (3) 30 days post-op | No specific questions reported. A structured questionnaire was used. | Overall: 5.6%  (1) 2–4 h post-op: 4.2%  (2) 24–36h post-op: 3.9%  (3) 30 days post-op: 3.4% | Dreamers: 4%  Non-dreamers: 7%  (Relied on behaviour as assessed by anaesthetists, not on patients’ reports) |
| Aceto et al. (2007)^86^ | yes | Italy | RCT | 58 | M = 47 ± 11; Range: 18 – 65 | 60% F, 40% M | Elective laparoscopic cholecystectomy | Propofol + Remifentanil | Propofol + Remifentanil | - | (1) Immediately upon opening eyes;  (2) 24h post-op | Immediately: No specific questions reported. Patients were asked whether they recalled any dreams, and if so, to describe the details.  24h post-op: Modified Brice interview (Brice et al., 1970) was used:  1. “What is the last thing you remember before falling asleep?”  2. “What is the first thing you remember after waking up?”  3. “Did you dream while you were asleep?”  4. “Did you hear any words or sounds while you were asleep?” | 10.3% | 0% |
| Leslie et al. (2007)^87^ | yes | Australia | Prosp. obs. | 300 | M = 35 ± 9;  Range: 18 – 50 | 63% F, 37% M | Pelvic/abdominal/thoracic,  head and neck,  orthopaedic | Anaesthetics were used according to the anaesthetists’ discretion:  Mostly Propofol, seven patients induced with Sevoflurane and three with Thiopentone bolus | Anaesthetics were used according to the anaesthetists’ discretion:  Propofol TCI,  Desflurane, Sevoflurane, N_2_O, Fentanyl, Remifentanil | Yes  (BIS > 60:  Mdn = 0; Range: 0–31  BIS = 40–60: Mdn = 33;  Range: 0 –100  BIS < 40:  Mdn = 66;  Range: 0 –100) | (1) Immediately upon emergence;  (2) 2-4h post-op | Modified Brice interview (Brice et al., 1970):  1. “What was the last thing you remember before going to sleep?”  2. “What was the first thing you remember when you woke up?”  3. “Can you recall anything between?”  4. “Did you have any dreams during your anaesthetic?” After narrative dream reports, the following five-point Likert scales were used: 1. Emotional content (from 1 = very negative to 5 = very positive) 2. Memorability (from 1 = can’t remember narrative of dream to 5 = most memorable ever) 3. Visual vividness (from 1 = not at all vivid to 5 = most vivid ever) 4. Amount of sound (from 1 = no sound to 5 = most sound ever) 5. Emotional intensity (from 1 = not at all intense to 5 = most intense ever) 6. Meaningfulness (from 1 = not at all meaningful to 5 = most meaningful ever) 7. Amount of movement (from 1 = no movement to 5 = most movement ever) 8. Strangeness (from 1 = not at all strange to 5 = most strange ever) | (1) Immediately upon emergence: 22%  (2) 2-3h post-op:  25% | 0.33%  (1 possible awareness, where dream content mixed with signs of awareness) |
| Toscano et al. (2007)^88^ | yes | Italy | RCT (Double-Blind) | 97  (100 enrolled) | 27 ± 6;  Range: 19 –39 | 100% F | Minor gynaecologic surgery | Propofol  (30 min before procedure, 50 patients received scopolamine and 50 received atropine) | Propofol | - | (1) 20 min after propofol stopped (2) 6h later, during discharge | No specific questions reported. Patients were asked whether they had had any dreams and whether the dream content was pleasant or unpleasant. | Scopolamine group: 0%  Atropine group: 46.9% | - |
| Errando et al. (2008)^89^ | no | Spain | Prosp. obs. | 3968 | M = 51 ± 18 | 57.7% F, 42.3% M | Various types of surgeries, with the most common being general-gastrointestinal, orthopaedic, and ear-nose-throat | Group 1: (TIVA): Propofol | Group 1 (TIVA): Propofol | - | (1) Immediately in PACU;    If awareness detected or considered possible, follow-up interviews:  (2) 7^th^ post-op day; and  (3) 30^th^ post-op day | A structured interview (available in the article). Dream-related questions included:  - “Do you remember having had dreams during surgery or anaesthesia?”  -“If yes, were these dreams pleasant, unpleasant, indifferent, or don’t remember how they were?” | Overall: 52.6%  Group 1: TIVA (Propofol): 52.9% | 1.0% |
|  |  |  |  |  |  |  |  | Group 2 (Balanced Anaesthesia): Any i.v. induction drug | Group 2 (Balanced Anaesthesia): Inhalation anaesthetics |  |  |  | Group 2: Balanced Anaesthesia: 53.7% |  |
|  |  |  |  |  |  |  |  | Group 3: (Mixed Anaesthesia): Any i.v. drug, | Group 3 (Mixed Anaesthesia): N₂O/O₂ |  |  |  | Group 3: Mixed Anesthesia:30.8% |  |
|  |  |  |  |  |  |  |  | Group 4 (Other Aesthetic Combinations): Various induction methods (e.g., bolus dose of Propofol for short procedures, Midazolam-Ketamine, and Midazolam- Opioids) | Group 4 (Other Aesthetic Combinations): Ketamine, Midazolam, Opioids |  |  |  | Group 4: Other Aesthetic Combinations: 53.0% |  |
| Samuelsson et al. (2008)^90^ | yes | Sweden | Prosp. obs. | 2653 | Specific ages were not given in the article, analyses in two groups: (1) ≤ 50; (2) > 50 | 56.8% F, 43.2% M | Different surgeries | Thiopentone  or  Propofol | Sevoflurane or Sevoflurane + N_2_O  or Propofol | Yes  (BIS; M < 40) | (1) Before discharge from PACU;  (2) 1-3 days post-op;  (3) 7-14 days post-op | Modified Brice interview (Brice et al., 1970). No specific questions reported. Patients were asked whether they could remember any dream that they had experienced during the anaesthesia. Dreams reported were classified, in accordance  with the patient’s own opinion, as unpleasant or pleasant/neutral. | Overall: 8.0%  (1) Before discharge from PACU: 6.1%  (2) 1-3 days post-op: 5.5%  (3) 7-14 days post-op: 4.6% | 0% |
| Samuelsson et al. (2008)^91^ | yes | Sweden | Prosp. obs. | 6991 | Mdn ≥ 48 (specific range not specified) | 56% F, 44% M | Various elective surgeries | Thiopentone  or  Propofol | Volatile anaesthetics (no specific agents mentioned) | - | (1) Before discharge from PACU;  (2) 1-3 days post-op;  (3) 7-14 days post-op | Modified Brice interview (Brice et al., 1970). No specific questions reported. Reported dreams were classified, in accordance with the patient’s own opinion,  as unpleasant or pleasant/neutral. | 3.3%  1) Before discharge from PACU: 0.2%  (2) 1-3 days post-op: 2.3%  (3) 7-14 days post-op: 2.3% | 0.1% |
| Stait et al. (2008)^92^ | yes | Australia | Prosp. obs. | 200 | M = 58 ±14; Range: 19 – 96 | 59% F, 41% M | Colonoscopy | Propofol + Midazolam and/or Fentanyl | Same as induction – intermittent IV bolus, not continuous infusion | - | Immediately upon emergence | Modified Brice interview (Brice et al., 1970):  1. “What was the last thing you remember before going to sleep?” 2. “What was the first thing you remember when you woke up?” 3. “Can you recall anything between?” 4. “Did you have any dreams during your colonoscopy?” If patients reported dreaming, they were also asked to rate the following: 1. Emotional content (from 0 = very negative to 5 = very positive). 2. Memorability (from 0 = only remember dreaming to 5 = most memorable ever). 3. Visual vividness (from 0 = not at all vivid to 5 = most vivid ever). 4. Amount of sound (from 0 = no sound to 5 = most sound ever). 5. Emotional intensity (from 0 = not at all intense to 5 = most intense ever). 6. Meaningfulness (from 0 = not at all meaningful to 5 = most meaningful ever). 7. Amount of movement (from 0 = no movement to 5 = most movement ever). 8. Strangeness (from 0 = not at all strange to 5 = most strange ever). | 25.5% | 4% |
| Bejjani et al (2009)^93^ | no | Belgium | RCT (Double-Blind) | 38 | 67 ± 11; Range: 51 – 77 | 32% F, 68% M | Elective cardiac surgery with cardiopulmonary bypass | Propofol | Propofol + Remifentanil | Yes  (BIS; range: 55 – 62) | 1^st^ post-op day (when patients were adequately responsive) | No specific questions reported. Patients were asked if they had recall of any event, and they were questioned about their last memory before falling asleep, their first memory on awakening, and if they had dreamed or had any recall in between. | 0% | 0% |
| Eer et al. (2009)^94^ | yes | Australia | Prosp. (Partial-RCT) | 200  (Group 1: 97; Group 2: 44; Group 3: 16; Group 3: 43) | >18 | 50.5% F,  49.5% M | Colonoscopy | Group 1: Propofol only  Group 2: Propofol + Midazolam  Group 3: Propofol + Fentanyl  Group 4: Propofol + Fentanyl + Midazolam | | Yes (BIS) | Before PACU discharge | Modified Brice interview (Brice et al., 1970):  1. “What was the last thing you remember before going to sleep?” 2. “What was the first thing you remember when you woke up?”  3. “Can you recall anything between?” 4. “Did you have any dreams during your colonoscopy?” | 19%  (not reported separately for groups) | Possibly 1 |
| Leslie et al. (2009)^95^ | yes | Australia | RCT (Double-Blind) | 300  (2 groups of 150) | Overall range: 18 – 50  Group 1: M = 36 ± 9 | 68% F,  32% M | Elective noncardiac surgery (gynaecological, general, other) | Fentanyl + Propofol | Group 1: Propofol TCI | Yes  (BIS; Anaesthesia titrated to BIS 40 - 55 during maintenance) | (1) Immediately upon emergence;  (2) 2-4h post-op | Modified Brice interview (Brice et al., 1970): 1. “What was the last thing you remember before going to sleep?”  2. “What was the first thing you remember when you woke up?”  3. “Can you recall anything between?”  4. “Did you have any dreams during your anaesthetic?” | Group 1 (Propofol): 27% | 0% |
|  |  |  |  |  | Group 2: M = 36 ± 9 |  |  |  | Group 2: Desflurane |  |  |  | Group 2 (Desflurane): 28% |  |
| Padmanabhan et al. (2009)^96^ | no | Australia | RCT (Single-Blind) | 200  (2 groups of 100) | ≥18 | 53.5% F,  46.5% M | Elective outpatient colonoscopy | Group 1: Propofol | Propofol | Yes (BIS) | Before PACU discharge | Modified Brice interview (Brice et al., 1970). No specific questions reported. | Group 1: 22% | - |
|  |  |  |  |  |  |  |  | Group 2: Propofol + Midazolam and/or Fentanyl |  |  |  |  | Group 2: 16% |  |
| Paech et al. (2009)^97^ | no | Australia and New Zealand | Prosp. obs. | 1095  (from 13 hospitals) | M = 30.4 ± 6.2 | 100% F | Caesarean section (emergency and elective) | The most common anaesthetics used: Thiopental (83% cases), Propofol (15% cases), Ketamine (1.6% cases) | The most common anaesthetics used: Sevoflurane (63% cases), Isoflurane (28% cases), Desflurane (8.5% cases), Propofol (1.3% cases) N_2_O (69% cases) | Yes  (BIS; used in 32% of cases) | (1) 2-6 h post-op;  (2) at least 48h post-op but before discharge from hospital | Modified Brice interview (Brice et al., 1970): 1. “What was the last thing you remember before going to sleep?’’ 2. “What was the first thing you remember when you woke up?’’  3. “Can you recall anything in between?’’  4. ‘‘Did you have any dreams during your anaesthetic?’’ In case of dreams, a narrative report was collected. | 3.3% | 0.3% |
| Xu et al. (2009)^98^ | no | China | Prosp. obs. | 11101 | M = 49 ± 16; Range: 14 –99 | 54.2% F, 45.8% M | Various types of surgery (neurosurgery, craniofacial and cervical, heart, obstetrics, chest and abdominal, urinary, spine and limbs, other) | Various anaesthetic techniques:  TIVA, inhalation anaesthesia. balanced anaesthesia, general anaesthesia combined with nerve blockage  (anaesthetic agents not specified), with or without Opiates | | - | (1) 1^st^ post-op day;  (2) 4^th^ post-op day | Modified Brice interview (Brice et al., 1970): 1. “What is the last thing you remembered before you went to sleep?” 2. “What is the first thing you remembered when you woke up?” 3. “Can you remember anything between these two periods?” 4. “Did you dream during your operation?” 5. “What was the worst thing about your operation?” | 3.2% | Definite: 0.4% Possible 0.4% |
| Cheong et al. (2011)^99^ | yes | South Korea | RCT | 125 (Suggestion group: 62, Control group: 63) | Overall range: 20 – 65  Suggestion group: M = 44.6 ± 12.6; | 52.8% F, 47.2% M | Surgical procedure on the lower extremities, genitals, or perianal area | (1. spinal anaesthetic bupivacaine) +  2. i/v Ketamine | | Yes  (>70 in most patients) | In the recovery room | 1. “Have you dreamed, do you remember what the dream was about?” 2. “Please tell me the contents of the dream, and indicate whether any of the following moods occurred in the dream. Use a number from 1 to 5, where 1 is very unpleasant, 2 is quite unpleasant, 3 is neither or mixed, 4 is quite pleasant, and 5 is very pleasant.” | Suggestion group: 83.9% | - |
|  |  |  |  |  | Control group: 44.3 ± 14.1 |  |  |  |  |  |  |  | Control group: 79.4% |  |
| Kim et al. (2011)^100^ | yes | South Korea | RCT | 215  (Group 1: 108; Group 2: 107) | Range: 18 - 70 | 26% F,  74% M | Deep sedation during spinal anaesthesia | Group 1: Propofol | | - | (1) When the patients initially emerged from sedation and were oriented to the time, place, and person  (2) 30min later | Modified Brice interview (Brice et al., 1970): 1. “What was the last thing you remember before going to sleep?” 2. “What was the first thing you remember when you woke up?” 3. “Can you recall anything in between?” 4. “Did you have any dreams during sedation?” Patients also provided a narrative dream report and rated dreams on a 5-point scale: (1) Emotional content (from 1 = very negative to 5 = very positive 2. Memorability (from 1 = only remember dreaming to 5 = very memorable). 3. Visual vividness (from 1 = not at all vivid to 5 = very vivid). 4. Emotional intensity (from 1 = not at all intense to 5 = very intense). 5. Strangeness (from 1 = not at all strange to 5 = very strange). | Group 1 (Propofol): 39.8% | - |
|  |  |  |  |  |  |  |  | Group 2: Midazolam | |  |  |  | Group 2 (Midazolam): 12.1% |  |
| Zhang et al. (2011)^101^ | no | China | RCT (Double-Blind) | 5228  (BIS group: 2919; No-BIS group: 2309) | BIS group: M = 46.95 ± 14.86; | BIS group: 57.2% F, 42.8% M; | Various types of surgery (neurosurgery, craniofacial and cervical surgery, heart surgery, gynaecological and obstetric surgery, chest and abdomen surgery, urinary surgery, spine and limb surgery, other) | Midazolam + Propofol (TIVA) | Midazolam + Propofol (TIVA) | Yes  (BIS group: between 40-60)  vs  No (No-BIS group: BIS was measured but not monitored online) | (1) 1st post-op day;  (2) 4th post-op day | Modified Brice interview (Brice et al., 1970): 1. “What is the last thing you remembered before you went to sleep?”  2. “What is the first thing you remembered when you woke up?”  3. “Can you remember anything between these two periods?”  4. “Did you dream during your operation?”  5. “What was the worst thing about your operation?” | BIS group: 3.1% | BIS group: 0.1% |
|  |  |  |  |  | No-BIS group: M = 46.06 ± 14.59 | No-BIS group: 57.4% F, 42.6% M |  |  |  |  |  |  | No-BIS group: 3.1% | No-BIS) group: 0.7% |
| Xu et al. (2012)^102^ | yes | China | Prosp. obs. | 200 | Range: 20 – 45 | 100% F | Group 1: Upper gastrointestinal endoscopy; | Propofol + Fentanyl | None | Yes (BIS) | Immediately upon emergence | Modified Brice interview (Brice et al., 1970): 1. “What was the last thing you remember before going to sleep?”  2. “What was the first thing you remember when you woke up?”  3. “Can you recall anything between?”  4. “Did you have any dreams?” If dream was reported, patients rated dreams on a 5-point Likert scale: 1. Emotional content (from 1 = very negative to 5=very positive). 2. Memorability (from1 = cannot remember narrative of dream to 5 = most memorable ever experienced). 3. Visual vividness (from 1 = not at all vivid to 5 = most vivid ever experienced). 4. Amount of sound (from 1 = no sound to 5 = most sound ever experienced). 5. Emotional intensity (from 1 = not at all intense to 5 = most intense ever experienced). 6. Meaningfulness (from 1 = not at all meaningful to 5 = most meaningful ever experienced). 7. Amount of movement (from 1 = no movement to 5 = most movement ever experienced). 8. Strangeness (from 1 = not at all strange to 5 = strangest ever experienced) | Overall: 25.5%  Group 1: 18% | - |
|  |  |  |  |  |  |  | Group 2: Surgical abortion |  |  |  |  |  | Group 2: 33% |  |
| Xu et al. (2012)^103^ | yes | China | RCT | 200  (2 groups of 100) | Range: 20 – 46 | 100% F | Surgical abortion | Group 1: Propofol (iv bolus) | Group 1: Propofol (infusion) | Yes (BIS) | Immediately upon emergence | Modified Brice interview (Brice et al., 1970):  1. “What was the last thing you remember before going to sleep?” 2. “What was the first thing you remember when you woke up?” 3. “Can you recall anything between?”  4. “Did you have any dreams during your procedure?” If the patient reported dreaming under sedation, details of dream content were collected.  Characteristics of the dream were assessed with a 5-point Likert scale: 1. Emotional content (from 1 = very negative to 5 = very positive). 2. Memorability (from 1 = can’t remember narrative of dream to 5 = most memorable ever experienced). 3. Visual vividness (from 1 = not at all vivid to 5 = most vivid ever experienced). 4. Amount of sound (from 1 = no sound to 5 = most sound ever experienced). 5. Emotional intensity (from 1 = not at all intense to 5 = most intense ever experienced). 6. Meaningfulness (from 1 = not at all meaningful to 5 = most meaningful ever experienced). 7. Amount of movement (from 1 = no movement to 5 = most movement ever experienced). 8. Strangeness (from 1 = not at all strange to 5 = strangest ever experienced). | Group 1 (Propofol): 33% | - |
|  |  |  |  |  |  |  |  | Group 2: Sevoflurane | Group 2: Sevoflurane |  |  |  | Group 2 (Sevoflurane): 60% |  |
| Aceto et al. (2013)^104^ | yes | Italy | RCT | 106  (Experimental group: 54, Control group: 52) | Range: 18 – 70  Experimental group: M = 49.2 ± 12.8; | Experimental group:  41% F,  59% M; | Elective laparoscopic cholecystectomy | Propofol + Remifentanil | Sevoflurane + Remifentanil  (Experimental group: auditory presentation of a story during anaesthesia;  Control group: no auditory stimulation) | Yes  (BIS) | (1) Immediately upon opening eyes;  (2) 24h post-op | No specific questions reported.  (1) Immediately upon opening eyes: patients were asked whether they recalled any dreams, and if so, to describe the details.  (2) 24h post-op: Patients were asked questions from the Brice interview (Brice et al., 1970) plus whether they heard any words or sounds while they were asleep. | Experimental group: 31% | 0% |
|  |  |  |  |  | Control group: M = 48.11 ± 13.9 | Control group: 60% F, 40% M |  |  |  |  |  |  | Control group: 19% |  |
| Aktas et al (2013)^105^ | no | Turkey | Prosp. RCT-like (in 3 groups of 20) | 60 | Range: 18 – 80 | 93% F,  7% M | Elective abdominal surgery (laparoscopic and open abdominal) | Propofol + Remifentanil (TIVA) | Propofol + Remifentanil (TIVA)  (Participants in the 3 groups were presented different auditory stimulation during anaesthesia) | Yes  (BIS; values maintained between 40 – 60) | 2h post-op | 1. “Do you remember anything between the beginning and the end of the surgery?” 2. “Did you have any dreams during the surgery?” | 18% | 1.7% |
| Czarko et al. (2013)^106^ | no | Poland | RCT | 337 | Group 1: M = 48 ± 10 | 100% F | Elective gynaecological procedures and Caesarean sections. | Group 1: Propofol + Remifentanil (TIVA) | Group 1: Propofol + Remifentanil (TIVA) | Yes  (sleep depth was monitored using auditory evoked potentials with AEP monitor) | (1) 2 hours after anaesthesia;  (2) 7^th^ post-op day;  (3) 30^th^ post-op day | A questionnaire consisting of 22 questions. Dream-related questions: 5. “Did you dream during anaesthesia? (yes/no)” 6. “What dreams did you have? (pleasant/unpleasant/unidentified/I don't remember the dreams)” Questions relating to awareness: 9. “Do you remember anything from the period of surgery? (yes/no/I don't know)” 10. “What do you remember from the period of surgery? (enquire further)” 11. “Did you hear anything during surgery? (yes/no/I don't know)” 12. “What did you hear during surgery? (ask further about noises, conversations, voices, etc.)” 13. “Did you feel anything during surgery? (yes/no/I don't know)” 14. “What did you feel during surgery? (ask further about touch, pain, skin incision, pressure, presence of something in the mouth, throat, etc.)” | Group 1: 13.7% | 0.9% |
|  |  |  |  |  | Group 2: M = 44 ± 12 |  |  | Group 2: Thiopentone + Fentanyl | Group 2: Sevoflurane  + N₂O/O_2_ |  |  |  | Group 2: 8.4% |  |
|  |  |  |  |  | Group 3: M = 50 ± 15 |  |  | Group 3: Propofol + Fentanyl | Group 3: Sevoflurane  + N₂O/O_2_ |  |  |  | Group 3: 12.5% |  |
|  |  |  |  |  | Group 4: M = 31 ± 6 |  |  | Group 4: Thiopentone + Fentanyl | Group 4: Sevoflurane  + N₂O/O_2_ |  |  |  | Group 4: 14.9% |  |
| Matsuyama et al. (2013)^107^ | yes | Japan | Retrosp. cohort | 2704 | M = 58 ± 17 | 52.7% F, 47.3% M | Different types of elective surgery (abdominal, obstetric, gynaecological, urological, orthopaedic, otolaryngological, thoracic, cardiovascular, and other surgeries) | - | Different maintenance agents:  Inhalational Anaesthesia: N_2_O + Isoflurane or Sevoflurane (45.6% cases)  Inhalational Anaesthesia with Epidural: N_2_O + Isoflurane or Sevoflurane + Epidural (15.1% cases)  TIVA: Propofol TCI + Remifentanil (8.8% cases)  Intravenous: Propofol TCI + Remifentanil + Epidural (30.5% cases) | - | Post-op (specific times not reported) | "Have you recalled dreaming during anaesthesia postoperatively?" | 3.3% | - |
| Russell (2013)^108^ | no | UK | Prosp. obs. | 34 | M = 47.2 ± 9.8 | 100% F | Gynaecological surgery | Fentanyl + Propofol | Isoflurane | Yes  (BIS; The goal was to maintain between 55-60) | Before returning to the ward | Modified Brice interview (Brice et al., 1970). Questions related to dreams: 5. “What was the last thing you remember before going to sleep?” 6. “What was the first thing you remember on waking up?”  7. “Can you remember anything in between these two?” 8. “Did you have any dreams?” | 3% | 0% |
| Xu et al. (2013)^109^ | yes | China | Prosp. obs. | 200 | Range: 25 – 68 | 50% F, 50% M | Upper gastrointestinal endoscopy | Propofol (bolus) + Fentanyl | Propofol (infusion) | - | Immediately upon emergence | Modified Brice interview (Brice et al., 1970): 1. “What was the last thing you remember before going to sleep?” 2. “What was the first thing you remember when you woke up?” 3. “Can you recall anything between?” 4. “Did you dream?” If the patient reported dreaming, details of dream content were collected.  Characteristics of the dream were assessed with a 5-point Likert scale: 1. Emotional content (from 1 = very negative to 5 = very positive). 2. Memorability (from 1 = can’t remember narrative of dream to 5 = most memorable ever experienced). 3. Visual vividness (from 1 = not at all vivid to 5 = most vivid ever experienced). 4. Amount of sound (from 1 = no sound to 5 = most sound ever experienced). 5. Emotional intensity (from 1 = not at all intense to 5 = most intense ever experienced). 6. Meaningfulness (from 1 = not at all meaningful to 5 = most meaningful ever experienced). 7. Amount of movement (from 1 = no movement to 5 = most movement ever experienced). 8. Strangeness (from 1 = not at all strange to 5 = strangest ever experienced). | 31% (for men);  17% (for women) | - |
| Tezcan et al. (2014)^110^ | no | Turkey | Prosp. obs. | 50 | M = 47.2 ± 16.3 | 66% F, 34% M | Colonoscopy | Propofol | Propofol (additional injections as needed) | - | 15 min after recovery (orientation to the time and place) | Modified Brice interview (Brice et al., 1970): 1. “What was the last thing you remembered before going to sleep?”  2. “What was the first thing you remembered when you woke up?”  3. “Can you recall anything between?”  4. “Did you have any dreams during your anaesthesia?” If dreaming reported, then 5-point Likert scales administered to assess memorability, emotional content, visual vividness, emotional intensity, and strangeness | 42% | - |
| Aceto et al. (2015)^111^ | yes | Italy | RCT | 127  (BIS group: 63; HP group: 64) | BIS group: M = 46.9 ± 14.2; | 72% F, 28% M | Elective thyroidectomy | Propofol + Fentanyl | Sevoflurane | Yes  (BIS group: anaesthesia titrated to maintain BIS between 40-50;  Non-BIS group: BIS was recorded but not available) | (1) Immediately upon opening eyes;  (2) 24h post-op | (1) Immediately upon opening eyes: whether patients recalled any dreams, and if so, to describe the details.  (2) 24h post-op: Questions from Brice interview (Brice et al., 1970). No specific questions reported but patients were asked about the last thing they remembered before falling asleep, the first thing they remembered when they woke up, and anything which happened in between, including sounds, dreams, and imagination. | Overall: 22.8%  BIS group: 27% | 0% |
|  |  |  |  |  | Non-BIS group: M = 45.9 ± 13.5 |  |  |  |  |  |  |  | Non-BIS group: 18% |  |
| Gyulaházi et al. (2015)^112^* | yes | Hungary | RCT | 270 (Suggestion group 1: 97; Suggestion group 2: 90; Control group: 82) | 11-18y: n = 20 19-30y: n = 91  31-50y: n = 78 51-75y: n = 79 >75y: n = 2 | 62.6% F, 37.4% M | Elective maxillofacial surgery | In all 3 groups, 3 subgroups:  Subgroup 1: Etomidate | In all 3 groups, 3 subgroups:  Subgroup 1: Sevoflurane | Yes  (BIS kept between 40-60; M = 41.37) | (1) 10min after recovery;  (2) 60min after recovery | - | Spontaneous dreams during anaesthesia: 35%  Subgroup 1 (Etomidate + Sevoflurane): group: 40% | - |
|  |  |  |  |  |  |  |  | Subgroup 2: Propofol | Subgroup 2: Sevoflurane |  |  |  | Subgroup 2 (Propofol + Sevoflurane): 26% |  |
|  |  |  |  |  |  |  |  | Subgroup 3: Propofol | Subgroup 3: Propofol (TIVA) |  |  |  | Subgroup 3 (Propofol TIVA): 39% |  |
| Chikkahanumanthappa et al. (2016)^113^ | yes | India | RCT | 120  (2 groups of 60) | Range: 30 – 60 | 100% F | Elective gynaecological surgeries | Group 1: Dexmedetomidine | Group 1: Dexmedetomidine (as needed) | - | (1) Upon emergence;  (2) 30 minutes later in the recovery room | No specific questions reported, but patients were questioned on incidences of dreaming and recall. | Group 1 (Dexmedetomidine): 3% | - |
|  |  |  |  |  |  |  |  | Group 2: Midazolam | Group 2: Midazolam (as needed) |  |  |  | Group 2 (Midazolam): 16% |  |
| Gyulaházi et al. (2016)^114^* | yes | Hungary | RCT | 270  (Suggestion group 1: 100); Suggestion group 2: 90; Control group: 80;  The two suggestion groups were combined in the analyses; n = 190) | Suggestion groups:  11–18y: n = 15  19–30y: n = 66  31–50y: n = 57  51–75y: n = 50  75 < y: n = 2  Control groups:  11–18y: n = 5  19–30y: n = 25  31–50y, n = 21  51–75y: n = 29  75 < y: n = 0 | Suggestion groups:  62.1% F, 37.9% M;  Control group: 63.7% F, 36.3% M | Surgeries related to accidents, cancer, inflammatory conditions, reconstruction, other | In all 3 groups, 3 subgroups:  Subgroup 1: Etomidate  Subgroup 2: Propofol  Subgroup 3: Propofol | In all 3 groups, 3 subgroups:  Subgroup 1: Sevoflurane  Subgroup 2: Sevoflurane  Subgroup 3: Propofol (TIVA) | Yes  (BIS kept between 40-60) | (1) 10min after recovery;  (2) 60min after recovery | - | Suggestion groups:  (1) 10min after recovery: 43.2%  (2) 60min after recovery: 37.4%  Control groups:  (1)10min after recovery: 20%  (2) 60min after recovery: 16.3% | - |
| Kabukcu et al. (2016)^115^ | no | Turkey | RCT | 70  (BIS group: 35, Non-BIS group: 35) | BIS group:  M = 58.7 ± 11.4) | - | Open heart surgery | Fentanyl + Etomidate | Propofol + Remifentanil | Yes  (BIS attached in both groups but monitored by the anaesthesiologist only in Group1; BIS kept between 35 - 45) | 1^st^ post-op day | Modified Brice interview (Brice et al., 1970):  1. “What is the last thing you remember before you fell asleep?”  2. “What is the first thing you remembered when you woke up?”  3. “Do you have any memories between these two events?”  4. “Did you dream during anaesthesia and surgery?”  5. “What is the most unpleasant thought you had during the intervention?” | BIS group: 2.9% | 1.4% |
|  |  |  |  |  | Non-BIS group:  M = 56.2 ± 10.4 |  |  |  |  |  |  |  | Non-BIS group: 2.9% |  |
| Cascella et al. (2017)^116^ | yes | Italy | Prosp. obs. | 51 | M = 29.35 ± 9.24 | 100% F | Day-case breast surgery (fibroadenoma excision) | Propofol (TCI) | Propofol (TCI) | Yes  (BIS kept between 60 - 75) | (1) Upon emergence;  (2) 24 hours post-op;  (3) 1 month post-op;  (4) 6 months post-op | Modified Brice interview (Brice et al., 1970) questionnaire: “1. What is the last thing you remember before going to sleep? 2. What is the first thing you remember when you woke up? 3. Can you recall anything in between going to sleep and waking up? 4. Did you have any dreams during your procedure? 5. Please assign a score for expressing your satisfaction with the sedation” (0 no satisfaction, 100 maximum satisfaction)” | Upon emergence: 12%  Across all follow-ups: 14% | 0% |
| Kundra et al. (2017)^117^ | no | India | RCT (Double-Blind) | 500  (2 groups of 250) | Group 1: 56.76 ± 10.33 | Group 1:  50.4% F, 49.6% M; | Elective cardioversion for supraventricular arrhythmias | Group 1: Dexmedetomidine + Propofol | Propofol | - | After full recovery | Modified Brice interview (Brice et al., 1970): 1. “What was the last thing you remembered before the procedure?” 2. “What was the next thing you remember?” 3. “Can you remember anything in-between these two periods?” 4. “Can you remember dreaming during your procedure?” 5. “If yes, did you notice sounds or conversations, paralysis, touch or movement, visual perceptions or pain.” | Group 1 (Dexmedetomidine + Propofol): 14% | - |
|  |  |  |  |  | Group 2: 55.06 ± 11.29 | Group 2: 52.8% F, 47.2% M |  | Group 2: Saline + Propofol |  |  |  |  | Group 2 (Propofol): 31.6% |  |
| Villegas et al. (2019)^118^ | no | Kenya | Prosp. obs. | 47  (37 completed the follow-up interview) | 38 ± 12 | 93.6% F, 6.4% M | Procedural sedation (78.7%) and various emergency surgeries (21.3%) | Ketamine | Ketamine | - | (1) Within 24h after procedure and prior to discharge;  (2) By telephone 4 -6 weeks later | 1. “Do you recall dreaming during the procedure?” 2. “If so, was your dream:  Pleasant  Frightening Neither  I do not remember” | 56.8% (procedural sedation)  50% (emergency surgery) | 24.3% (procedural sedation)  20% (emergence surgery) |
| Chen et al. (2020)^119^ | yes | China | RCT (Double-Blind) | 80  (Group 1: 40; Group 2: 40) | Overall range: 37 – 71  Group 1: M = 54.5 ± 8.7 | 46.2% F, 53.8% M | Flexible bronchoscopy | Group 1: (Pre-induction Dexmedetomidine) + Fentanyl + Propofol | Propofol (TIVA) | Yes (BIS) | Immediately upon emergence | Modified Brice interview (Brice et al., 1970): 1. “What is the last thing you remember before going to sleep?” 2. “What is the first thing you remember when you woke up?” 3. “Can you remember anything between going to sleep and waking up?” 4. “Did you dream during your procedure?”  Patients who reported dreaming completed 5-point Likert scale: 1) Dream is pleasant or unpleasant: from 1 = very unpleasant; to 5 = very pleasant; 2) Memorability of dream: from 1 = not remember at all to 5 = remember completely; 3) Talking in dream: from 1 = no talking at all to 5 = constant talking; 4) Action in dream: from 1 = no action to 5 = constant action. | Group 1 (Dexmedetomidine pre-induction): 17.5% | - |
|  |  |  |  |  | Group 2: M = 55.6 ± 7.3 |  |  | Group 2: (Pre-induction Midazolam) + Fentanyl + Propofol |  |  |  |  | Group 2 (Midazolam pre-induction): 37.5% |  |
| Hao et al. (2020)^120^ | yes | China | RCT | 300  (3 groups of 100) | Range: 20 – 65 | 50% F, 50% M | Painless gastroscopy | Group 1. Propofol | If needed during the procedure, a top-up dose | - | 5 min after emergence | Specific questions not reported. Patients were evaluated whether they were dreaming or not and the characteristics of dreams during anaesthesia | Overall: 35%  Group 1 (Propofol): 54.8% | 0% |
|  |  |  |  |  |  |  |  | Group 2: Propofol + Etomidate (10ml) |  |  |  |  | Group 2 (Propofol + 10ml Etomidate): 15.7% |  |
|  |  |  |  |  |  |  |  | Group 3: Propofol + Etomidate (5ml) |  |  |  |  | Group 3 (Propofol + 5ml Etomidate): 41.2% |  |
| Yoshida et al. (2021)^121^ | yes | Japan | Prosp. obs. | 1100 | M = 60.5 ± 15.6 | 51.5% F, 48.5% M | Different types of elective surgeries (not specified) | - | Propofol + Remifentanil or  Sevoflurane + Remifentanil or  Desflurane + Remifentanil | Yes  (BIS kept between 40 – 60) | Immediately upon emergence | Modified Brice interview (Brice et al., 1970): 1. “What was the last thing you remember before going to sleep?” 2) “What was the first thing you remember from when you woke up?” 3) “Can you recall anything between?” 4) “Did you have any dreams while under anaesthesia?” 5) “How did you feel about your dream: pleasant, indifferent or unpleasant?” | 26.6% | 0% |
| Alam et al. (2022)^122^ | no | Pakistan | Prosp. obs. | 1000 | Rest of the sample: M = 46.9 ± 14.4  Patients who developed awareness: 26 ± 18.3 | 48.4% F, 51.6% M | Different types of elective and emergency surgeries (breast, gastrointestinal, gynaecological, hepatobiliary, maxillofacial/otolaryngology, neurosurgery, orthopaedic, thoracic, thyroid/parathyroid, urology) | Propofol (TIVA)  Or  Other i.v. induction agents (not specified) | Propofol (TIVA) or  Sevoflurane | Yes  (BIS values kept between 40 – 60) | (1) At the time of discharge from PACU; (2) Those with suspected occurrence of awareness were also interviewed 7 days post-op | Specific questions not reported. Modified Brice interview (Brice et al., 1970): patients were asked whether or not they had any disturbing dreams or recalled any intra-operative events during the current operation. | 0.2% | 0.2% |
| Chen et al. (2022)^123^ | no | China | RCT (Double-Blind) | 146  (2 groups of 73) | Range: 45 – 65  Group 1: M = 57.05 ± 5.60 | 26% F, 74% M | Flexible bronchoscopy. | Group 1: Remimazolam | Group 1: Remimazolam + Remifentanil; | - | Right before discharge from recovery room | Modified Brice interview (Brice et al., 1970). No specific questions reported. | Group 1 (Remimazolam + Remifentanil): 35.6% | - |
|  |  |  |  |  | Group 2: M = 56.05 ± 5.99 |  |  | Group 2: Dexmedetomidine | Group 2. Dexmedetomidine + Remifentanil |  |  |  | Group 2 (Dexmedetomidine + Remifentanil): 41.1% |  |
| Chow et al. (2022)^124^ | yes | USA | Case study | 1 | 26 | F | Surgical repair of dominant right hand | Fentanyl + Propofol (TIVA) | Propofol (TIVA) | Yes (SedLine) | Immediately upon emergence | - | Reported a dream | 0% |
| Ghanaie et al. (2022)^125^ | no | Iran | Prosp. obs. | 174 | Range: 20 – 46  M = 32.76 ± 5.74 | 100% F | Caesarean section | Propofol | Isoflurane + N₂O/O_2_ | - | After patients were fully awake | No specific questions reported. Patients were asked about the first and last memories before and after anaesthesia, as well as any intraoperative experiences of awareness. | 27.8% | 6.9% |
| Imantalab et al. (2022)^126^ | no | Iran | Prosp. obs. | 322 | M = 58.45 ± 6.8;  Range: 38 – 76 | 40.7% F,  59.3% M | Cardiac (exclusive coronary artery bypass surgery under cardiopulmonary bypass pump | Midazolam + Propofol | Propofol | Yes  (BIS kept between 40 – 60) | When the patient was well cooperative | Questionnaire in Persian from Arefian & Fathi (2007) | 1.9% | 5.3% |
| Matus et al. (2022)^127^ | yes | Croatia | Prosp. obs. | 124  (130 enrolled) | M = 53;  Mdn: 56; Range: 42.5 – 65 | 61% F, 39% M | Gastrointestinal endoscopy | Propofol (TIVA) | Propofol (TIVA) | Yes (BIS) | Upon emergence and before transferring patients to recovery room | No specific questions reported. Patients were asked whether they had any dreams during anaesthesia and whether their dreams were pleasant or not. | 43% | - |
| Schäfer et al. (2022)^128^ | yes | Germany | RCT | 80 (Suggestion group: 40; Control group: 40) | Suggestion group: M = 50 ± 22 | 41% F, 59% M | Maxillofacial surgery | Propofol + Remifentanil | Remifentanil | - | (1) 10min after emergence in the recovery room;  (2) 60min after emergence in the recovery room | Immediately upon emergence, patients were requested to keep their dreams in mind in order to be able to report them later. Then, a standardized interview was used. No specific questions reported but participants were asked to report their dreams. Emotionality of dreams was assessed by asking the patients whether their dreams were pleasant or not. All questions had limited answer options (yes vs. no, agreeable vs. not agreeable or rating scale). | Suggestion group: 57.5% | - |
|  |  |  |  |  | Control group: M = 57 ± 21 |  |  |  |  |  |  |  | Control group: 30.0% |  |
| Dikkala et al. (2023)^129^ | no | India | Prosp. obs. | 98 | Inclusion criteria: 18 – 60 (age of sample not reported) | - | Elective surgeries | Propofol or Thiopentone | N₂O/O_2_ + volatile anaesthetics (not specified) | Yes  (BIS; Basal: M = 93.47 ± 7.20; After 10 min: M = 46.29 ± 3.34; After 30 min: M = 47.81 ± 4.41; After 60 min: M = 49.83 ± 4.76; After 90 min: M = 51.01 ± 4.16; After 120 min: M = 52.31 ± 6.77; 10 min before reversal: M = 80.26 ± 7.84 | (1) 30min post-extubation;  (2) 1^st^ post-op day | Modified Brice interview (Brice et al., 1970):  (1) “What is the last thing you remember before you went to sleep for your operation?”  (2) “What is the first thing you remember after your operation?”  (3) “Can you remember anything in between?”  (4) “Did you dream during your operation?” (5) “What was the worst thing about your operation?” | 1.0% | 1.0% |
| Zhao et al. (2023)^130^ | no | China | Prosp. obs. | 360 | M = 43.97 ± 12.56 | 60.2% F,  39.7% M | Gastric or gastrointestinal endoscopy | Fentanyl or Sufentanil + Propofol and/or Etomidate. | Propofol (continuous infusion) | - | 30min post-op | No specific questions reported. Patients were asked briefly whether they had a dream and, if so, whether the dreams were pleasant. | 31.7% | - |
| Clinical Studies on Children/Adolescents | | | | | | | | | | | | | | |
| McKie & Thorp (1973)^131^ | yes | Australia | Prosp. obs. | 202 | M = 10.4y;  Range: 7 – 14y | 39.6% F,  60.4% M | Different surgical procedures (general, ear/nose/throat, orthopaedic, eye, thoracic, neurosurgery) | Different anaesthetics and their combinations used: Ketamine, Thiopentone, Halothane, N_2_O/O_2_; Methoxyflurane  (Also included patients who were sedated) | | - | (1) 72h post-op (interview);  (2) 1 month post-op (questionnaire) | 72h post-op:  “2. What is the last thing you remember before going to sleep?  […]  4. What is the next thing you remember, after going to sleep?  5. Do you remember having any odd feelings or seeing or hearing anything during your operation?”  1 month post-op: A questionnaire was sent home to which parents responded: "Would you ask (your child) if he remembers any of the following happening between the time he went to sleep for his operation and the time he woke up again. (1) Hearing noises of any sort? (2) Hearing anyone talking? (3) Seeing any lights, any people, or anything else? (4) Feeling anything at all? (5) Having any dreams during the operation ? If yes, describe the dreams (6) Does he remember anything else happening?” | 5.9%  (interview + questionnaire) | 2.5%  (interview + questionnaire) |
| Feingold & MacMahon (1975)^132^ | no | USA | Case study | 1 | 17y | F | Debridement and dressing changes | 17 anaesthetic procedures: First ten with Halothane + N_2_0 (3 times), Enflurane + N_2_0 (4 times), Fluroxene + N_2_0 (once), Thiopental + N_2_0 (once), Innovar® + N_2_0.  The 11th procedure involved N_2_0 +Thiopental. The next 6 involved N_2_0 + Ketamine. | | - | - | - | 5.9% (11^th^ procedure involving N_2_0 +Thiopental) | - |
| Elliott et al. (1976)^133^ | no | UK | Prosp. obs. | 61 | Range: 17m – 13y | - | Various procedures (lumbar puncture, bone marrow aspiration, chest aspiration or chest drain insertion and pericardial aspiration, skin dressings, other procedures) | Ketamine i.v. | Ketamine i.v. | - | - | - | 6.6% | - |
| Krantz et al. (1980)^134^ | no | South Africa | Prosp. obs. | 60  (12 patients interviewed postoperatively) | - | - | Outpatient procedures (incision an drainage of abscesses, meibomian cysts, removal of foreign body, excision of dermoid cyst, probing of lacrimal duct, insertion of scleral shell, and digital examination) | Ketamine i.m. | N_2_O/O_2_ | - | Post-op  (no more details provided) | No specific questions reported. Patients were interviewed postoperatively about dreams or unpleasant sensations. | 50% | - |
| Hobbs et al. (1988)^135^ | yes | UK | Prosp. obs. | 120 | M = 9y; Range: 5 – 17y | 34.2% F, 65.8% M | Various procedures (ear/nose/throat, dental, ophthalmology, orthopaedic, plastic, and general surgery) | Sodium thiopentone | N_2_O/O_2_ | - | Before discharge | Modified Brice interview (Brice et al., 1970), but no specific questions reported. | 19% | 0% |
| O'Sullivan et al. (1988)^136^ | yes | UK | Prosp. obs. | 144 | Range: 5 – 14y | 41.7% F, 58.3% M | Various procedures (ear/nose/throat, ophthalmic, dental, urogenital, orthopaedic) | Thiopentone (Some patients were premedicated with tubocurarine) | N_2_O/O_2_ | - | Before discharge on the day of surgery | Modified Brice interview (Brice et al., 1970): 1. “Were you upset or worried about your operation?” 2. “What is the last thing you remember before going to sleep?” 3. “How did the doctor put you to sleep?” 4. “What is the next thing you remember after going to sleep?” 5. “Did you have any dreams or odd feelings while you were asleep?” 6. “Were you sore after the operation?” What did you like least about having an operation? Do you have dreams at home?” | Overall: 9.7%  Non-premedicated group: 16.7%  Premedicated group: 2.8% | 0% |
| Maldini (1996)^137^ | no | Croatia | Retrosp./prosp. obs. | 547 | M = 4.5y; Range: 2m – 14y | 28.7% F, 71.3% M | Necrectomy, skin grafting, debridement, and dressing changes for burn treatment | Ketamine i.v. | Ketamine i.v. | - | (1) 2h post-op;  (2) 1^st^ post-op day | No specific questions reported. Anaesthesia nurse asked patients especially about their dreams. | 0% | - |
| Tamminga et al. (2000)^138^ | no | Netherlands | RCT (Double-Blind) | 16 | Mdn = 8.5; Range: 4 – 16y | 31.2% F, 68.8% M | Bone marrow punctures | Ketamine i.v. | Ketamine i.v. | - | 45min after procedure | 1. “How do you feel?” 2. “How was awakening?” 3. “How was falling asleep?” 4. “Did you have dreams? If so, can you tell more about your dreams?” 5. “Was the procedure different from previous ones?” | 5.3% | - |
| Wathen et al. (2000)^139^ | no | USA | Field RCT (Double-Blind) | 266  (Group 1: 129; Group 2: 137) | 4.5m – 16y; Group 1: Mdn = 6.8; | Group 1: 43.4% F, 56.6% M; | Fractures, lacerations, other | Group 1: Ketamine (i.v.) | - | - | - | Observed by nurse | Group 1 (Ketamine): 10.9% |  |
|  |  |  |  |  | Group 2: Mdn = 5.6 | Group 2: 44.5% F, 55.5% M |  | Group 2: Ketamine (i.v.) + Midazolam (i.v.) |  |  |  |  | Group 2 (Ketamine + Midazolam): 4.4% |  |
| Huang et al. (2005)^140^ | yes | Australia | Prosp. obs. | 864 | Range: 5 – 12y | 43% F,  57% M | Various procedures (ear/nose/throat, general surgery, orthopaedic, dental, plastic, dermatological, gastroenterological, ophthalmological, radiological, gynaecological, urological, respiratory, other) | Propofol  or  Sevoflurane  or  Halothane  or Thiopentone | Isoflurane  or  Sevoflurane  or  Halothane  or  N_2_O  or  Propofol | - | (1) Day of anaesthesia or next day (if on the day of anaesthesia was not possible); (2) 3 days after anaesthesia (by child’s parents);  (3) 30 days after anaesthesia (by child's parents) | Modified Brice interview (Brice et al., 1970):  1. “What was the last thing you remember before the operation?” 2. “What is the next thing you remember after the operation?”  3 “Did you have any dreams or feel or hear anything while you were having the operation?” | 10.4% | 0.8% |
| da Silva et al. (2011)^141^ | no | Brazil | Prosp. obs. | 20 | Mdn (25-75th percentile) = 8y (5 – 10y) | 40% F, 60% M | Bone marrow aspiration | Ketofol (1:1 Ketamine + Propofol) (i.v.) | Ketofol (i.v.; additional boluses) | - | Post-procedure, once the patients were fully awake and oriented | - | 5% | 0% |
| Experimental Studies | | | | | | | | | | | | | | |
| Wilson et al. (1973)^142^ | no | USA | Within-subject (repeated measures) | 13 | M = 23.6;  Range 22 – 25 | 100% M | - | CL-1848C (Etoxadrol; i.v.) | - | Yes (EEG) | - | - | 76.9% | - |
| Morse et al. (2004)^143^ | no | Japan | Within-subject (repeated measures) | 10 | M = 29 | - | - | Midazolam + Ketamine (boluses) | Midazolam + Ketamine + Saline (infusion) delivered over1h at a constant rate | - | - | - | 100% | - |
| Noreika et al. (2011)^144^ | yes | Finland | Between-subject with repeated measures in some groups | 40  (4 groups of 10) | 18 - 30 | 100% M | - | All groups: LOR induced by escalating stepwise increases in concentrations (except for more abrupt Xenon sessions).  Group 1: Dexmedetomidine (TCI)  Group 2: Propofol (TCI)  Group 3: Sevoflurane  Group 4: Xenon | | Yes (BIS and EEG) | 5 min upon return of responsiveness | Structured interview: 1. “Tell me everything that was going on in your mind starting from anaesthesia induction to the awakening from anaesthesia.” 2. “If you had some experiences related to the laboratory surroundings or the experiment, describe them as precisely as possible.” 3. “If you had some moods or feelings, specify them.” 4. “If you had visual experiences, list the figures, objects and characters you have seen.” 5 “If you had some experiences of your own body or self, describe them.” 6. “If some changes happened in your experiences, for example one object or feeling was replaced by another, describe them.” 7. “Tell me if you remember something else that is not reported yet.” Additional open questions were asked depending on the report. | Session 1: 62.5%  Group 1 (Dexmedetomidine): 90%  Group 2 (Propofol): 30%  Group 3 (Sevoflurane): 60%  Group 4 (Xenon): 70%  All sessions (2 sessions): 58.6%  Group 1 (Dexmedetomidine): 73.7%  Group 2 (Propofol): 36.8%  Sevoflurane): 60%  Group 4 (Xenon): 70% | - |
| Sarasso et al. (2015)^145^ ‡ | yes | Belgium | Between-subject (within-condition comparison) | 18  (3 groups of 6) | 18 - 28 | 56% F,  44% M | - | Group 1: Propofol (TIVA) | Group 1: Propofol (TIVA) | Yes (EEG used but not for monitoring anaesthetic depth) | (1) Immediately upon awakening  (2) Report confirmed with participants 1h after recovering consciousness | “What was going on through your mind before awakening?” | Group 1 (Propofol): 0% | - |
|  |  |  |  |  |  |  |  | Group 2: Propofol bolus + Xenon | Group 2: Xenon |  |  |  | Group 2 (Xenon): 0% |  |
|  |  |  |  |  |  |  |  | Group 3: Ketamine (i.v.) | Group 3: Ketamine (i.v. infusion) |  |  |  | Group 3 (Ketamine): 100% |  |
| Bonhomme et al. (2016)^146^ | no | Belgium | Within-subject (repeated measures) | 8  (14 enrolled) | M = 25;  Range: 19-31 | 36% F, 64% M | - | Ketamine (computer-controlled i.v. infusion) was step-wise increased in steps until LOR | | Yes (EEG used but not for monitoring anaesthetic depth) | By phone on average 327 days after the experiment (range: 66-821 days) | No specific questions reported. A detailed description of dream themes was recorded. | 50% | - |
| Darraq et al. (2018)^147^ ‡ | yes | Belgium, USA | Secondary analysis of Sarasso et al. (2015) | 12  (Group 1: 6; Group 2: 6) | - | - | - | Group 1: Propofol (TIVA)  Group 2: Ketamine (i.v.) | Group 1: Propofol (TIVA)  Group 2: Ketamine (i.v. infusion) | Yes (EEG used but not for monitoring anaesthetic depth) | Same as in Sarasso et al. (2015) | Same as in Sarasso et al. (2015) | Same as in Sarasso et al. (2015) | - |
| Radek et al (2018)^148^** | yes | Finland | RCT (randomized open-label, parallel-group) | 47  (Group 1: 23; Group 2: 24) | M = 24;  Range 20 -30 | 100% M | - | All groups: Increasing step-wise concentrations till LOR  Group 1: Dexmedetomidine (TCI) | | Yes (EEG used but not for monitoring anaesthetic depth) | After every successful awakening, immediately upon emergence | Modified Brice interview (Brice et al., 1970): 1. “Did you dream during unresponsiveness?” 2. “Did you experience anything related to the research environment during unresponsiveness?” 3. “Did you hear anything during unresponsiveness?” 4. “Did you sense anything (else) during unresponsiveness?” 5. “Do you remember anything else that you have not already mentioned?” Additional questions asked after the last return of responsiveness: 6. “What is the last thing you remember before falling asleep for the first time?” 7. “What is the first thing you remember after awakening?” If participants answered yes to any question, they were asked to describe the experience in as much detail as possible. | Overall: 85.9%  Group 1 (Dexmedetomidine): 92.5% | Overall: 16.7%  Group 1 (Dexmedetomidine): 22.6% |
|  |  |  |  |  |  |  |  | Group 2: Propofol (TCI) | |  |  |  | Group 2 (Propofol): 72% | Group 2 (Propofol): 4% |
| Colombo et al. (2019)^149^ ‡ | Yes | Belgium | Secondary analysis of Sarasso et al. (2015) | Same sample as in Sarasso et al but n = 15 (3 groups of 5) | Range: 18 - 28 | 67% F,  33% M | - | Group 1: Propofol (TIVA)  Group 2: Propofol bolus + Xenon  Group 3: Ketamine (i.v.) | Group 1: Propofol (TIVA)  Group 2: Xenon  Group 3: Ketamine (i.v. infusion) | Yes (EEG used but not for monitoring anaesthetic depth) | Same as in Sarasso et al. (2015) | Same as in Sarasso et al. (2015) | Same as in Sarasso et al. (2015) | - |
| Radek et al. (2021)^150^*** | yes | Finland | RCT (randomized open-label, parallel-group) | 133  (Group 1: 40, Group 2: 39, Group 3: 35, Group 4: 19; 160 enrolled) | M = 24.1 ± 2.9;  Range: 18-30 | 100% M | - | All groups: 20min stabilization phase followed by 40min steady-state anaesthesia  Group 1: Dexmedetomidine (TCI) | | Yes (EEG used but not for monitoring anaesthetic depth) | Immediately upon emergence | Modified Brice interview (Brice et al., 1970):  1. “Did you dream during the anaesthetic administration?”  2. “Did you experience anything related to the research environment during the anaesthetic administration?”  3. “Do you remember anything else that you have not already mentioned?”  4. “What is the last thing you remember before falling asleep?”  5. “What is the first thing you remember after awakening?” | Overall: 45.9%  Group 1 (Dexmedetomidine): 68.4% | Overall: 2.2%  Group 1 (Dexmedetomidine): 0% |
|  |  |  |  |  |  |  |  | Group 2: Propofol (TCI) | |  |  |  | Group 2 (Propofol): 31.4% | Group 2 (Propofol): 0% |
|  |  |  |  |  |  |  |  | Group 3: Sevoflurane | |  |  |  | Group 3 (Sevoflurane): 25.0% | Group 3 (Sevoflurane): 0% |
|  |  |  |  |  |  |  |  | Group 4: S-Ketamine (TCI) | |  |  |  | Group 4 (S-Ketamine): 44% | Group 4 (S-Ketamine): 25% |
| Scheinin et al. (2021)^151^** | yes | Finland | Secondary analysis of Radek et al. (2018) | 39  (Group 1: 20; Group 2: 19) | Range: 20 - 30 | 100% M | - | Group 1: Dexmedetomidine (TCI)  Group 2: Propofol (TCI)  All groups: Increasing step-wise concentrations till LOR | | Yes (EEG used but not for monitoring anaesthetic depth) | In experiment 1, participants were asked whether dreaming had been present during the unresponsive period (yes/no). A more detailed interview followed after PET scan.  In experiment 2, interview was conducted immediately upon emergence. | Same as in Radek et al. (2018) | 80% | 5.1% |
| Casey, Tanabe, et al. (2022)^152^† | yes | USA | Within-subject (repeated-measures, serial awakening) | 26  (Group 1: 6; Group 2: 20) | Range: 18 - 40 | - | - | Group 1: Propofol (TCI) | | Yes (EEG used but not for monitoring anaesthetic depth) | Immediately after each "rest" period | Structured interview 1. “What was the last thing going through your mind before I spoke to you?”  2. “Do you think you were awake, having a dream, or were unconscious?”  3 “Do you think you were asleep or awake?”  4. “Were you aware of the external world?” | Group 1 (Propofol): 15.9% | Group 1 (Propofol):  44.3% |
|  |  |  |  |  |  |  |  | Group 2: Dexmedetomidine (infusion in steps) | |  |  |  | Group 2 (Dexmedetomidine): 45.8% | Group 2 (Dexmedetomidine): 15.2% |
| Casey et al. (2022)^153^ | yes | USA | Within-subject | 18  (20 enrolled) | Range: 18 - 32 | - | - | Dexmedetomidine (induction🡪infusion in steps)  Duration of anaesthesia limited to 4h | | Yes (EEG used but not for monitoring anaesthetic depth) | Immediately after each "rest" period | - | - | - |
| Guay et al. (2023)^154^ | no | USA | Within-subject, crossover trial | 16 | Range: 18 - 40 | - | - | Dexmedetomidine (TCI) Concentration increased in increments until LOR | | Yes (EEG used but not for monitoring anaesthetic depth) | - | Modified Brice interview (Brice et al., 1970). No specific questions reported. | 35.7% | - |
| Kantonen et al. (2023)^155^*** | yes | Finland | Secondary analysis of Radek et al. (2021) | 160  (Group 1: 40; Group 2: 40; Group 3: 40, Group 4: 20, Group 5: 20) | Range: 18 - 30 | 100% M | - | All groups: 20min stabilization phase followed by 40min steady-state anaesthesia  Group 1: Propofol (TCI) | | Yes (EEG used but not for monitoring anaesthetic depth) | After the end of steady-state anaesthesia | Same as in Radek et al. (2021). | Group 1 (Propofol): 36% | 0% |
|  |  |  |  |  |  |  |  | Group 2: Dexmedetomidine (TCI) | |  |  |  | Group 2 (Dexmedetomidine): 83% |  |
|  |  |  |  |  |  |  |  | Group 3: Sevoflurane | |  |  |  | Group 3 (Sevoflurane: 45% |  |
|  |  |  |  |  |  |  |  | Group 4: S-Ketamine (TCI) | |  |  |  | Group 4: None of subjects could be interviewed right after the drug infusion because of residual drug-effects |  |
|  |  |  |  |  |  |  |  | Group 5: Placebo | |  |  |  | Group 5: N/A |  |
| Valli et al. (2023)^156^** | yes | Finland | Secondary analysis of Radek et al. (2018) | 39  (Group 1: 20; Group 2: 19) | Range: 20 - 30 | 100% M | - | All groups: Increasing step-wise concentrations till LOR  Group 1: Dexmedetomidine (TCI) | | Yes (EEG used but not for monitoring anaesthetic depth) | Immediately after unresponsiveness periods. The median time from termination of infusion to full interview was 32.0 min (range: 8.5 - 61.1) in participants receiving dexmedetomidine and 14.9 min (range: 9.4 - 30.0) in participants receiving propofol. | "Did you have a dream?”, followed by modified Brice interview (Brice et al., 1970):  1. “Did you dream during anaesthesia/sleep?”  2. “Did you experience anything related to this room or situation during anaesthesia/sleep?”  3. “Did you hear anything during anaesthesia/sleep?”  4. “Did you sense anything during anaesthesia/sleep?”  5 “Do you remember anything else from during anaesthesia/sleep that you have not already mentioned?”  6. “What is the last thing you remember before falling asleep?” 7. “What is the first thing you remember after awakening?” | Overall: 62.3%  Group 1 (Dexmedetomidine): 51.6% | Overall: 5.7%  Group 1 (Dexmedetomidine): 3.2% |
|  |  |  |  |  |  |  |  | Group 2: Propofol (TCI) | |  |  |  | Group 2 (Propofol): 77.3% | Group 2 (Propofol): 9.1% |
| Casey et al. (2024)^157^ † | yes | USA | Secondary analysis of Casey, Tanabe et al. (2022a) | 26  (Dex: 20; Prop: 6) | Range: 18 - 40 | - | - | Same as in Casey, Tanabe, et al. (2022a) | Same as in Casey, Tanabe, et al. (2022a) | Yes (EEG used but not for monitoring anaesthetic depth) | Same as in Casey, Tanabe, et al. (2022a) | Same as in Casey, Tanabe, et al. (2022a). | Same as in Casey, Tanabe, et al. (2022a) | Same as in Casey, Tanabe, et al. (2022a) |

*Note*. Studies sharing the same superscript (*, **, ***, †, ‡) are based on overlapping datasets. Prosp. = Prospective. Prosp. obsv. = Prospective observational. RCT = randomized controlled trial. Retrosp. obsv. = retrospective observational. TCI = target-controlled infusion.

**References**

1.	Smith LG. Speech defect resulting from ether shock. Pedagogical Seminary 1921; Available from: https://www.tandfonline.com/doi/abs/10.1080/08919402.1921.10533994

2.	Hutchinson R. Awareness during surgery. Br J Anaesthesia 1961; 33: 463–9

3.	Brice DD, Hetherington RR, Utting JE. A simple study of awareness and dreaming during anaesthesia. Br J Anaesth 1970; 42: 535–42

4.	Harris TJB, Brice DD, Hetherington RR, Utting JE. Dreaming associated with anaesthesia: The influence of morphine premedication and two volatile adjuvants. Br J Anaesth 1971; 43: 172–8

5.	Smith JM, Utting JE, Whitford JH. Dreaming under anaesthesia. Br J Anaesth 1972; 44: 415–6

6.	Crawford JS, Davies P. A return to trichloroethylene for obstetric anaesthesia. Br J Anaesth 1975; 47: 482–90

7.	Hejja P, Galloon S. A consideration of ketamine dreams. Can Anaesth Soc J 1975; 22: 100–5

8.	Wilson SL, Vaughan RW, Stephen CR. Awareness, dreams, and hallucinations associated with general anesthesia. Anesthesia & Analgesia 1975; 54: 609

9.	Abouleish E, Taylor FH. Effect of morphine-diazepam on signs of anesthesia, awareness, and dreams of patients under N20 for cesarean section. Anesth Analg 1976; 55: 702–5

10.	Crawford JS, James FM, Davies P, Crawley M. A further study of general anaesthesia for caesarean section. Br J Anaesth 1976; 48: 661–7

11.	Downing JW, Mahomedy MC, Jeal DE, Allen PJ. Anaesthesia for caesarean section with ketamine. Anaesthesia 1976; 31: 883–92

12.	Famewo CE. Awareness and dreams during general anaesthesia for caesarian section a study of incidence. Canad Anaesth Soc J 1976; 23: 636–9

13.	Frederickson EL, Longnecker DE, Allen GW. Clinical Investigation of a new intravenous anesthetic—etoxadrol hydrochloride (CL-1848C; U-37862A). Anesth Analg 1976; 55: 335-9

14.	Freuchen I, Ostergaard J, Kuhl JB, Mikkelsen BO. Reduction of psychotomimetic side effects of Ketalar® (ketamine) by Rohypnol® (flunitrazepam): A randomized, double‐blind trial. Acta Anaesthesiol Scand 1976; 20: 97–103

15.	Mahomedy, MC, Downing, JW, Jeal DE, Allen PJ. Ketamine for anaesthetic induction at caesarean section. S Afr Med J 1976; 50: 846–8

16.	Ellingson A, Haram K, Sagen N. Ketamine and diazepam as anaesthesia for forceps delivery: A comparative study. Acta Anaesthesiol Scand 1977; 21: 37–40

17.	Figallo EM, Casali H, Mckenzie R, Tantisira B, Wadhwa RK, Taylor FH. Ketamine as the sole anaesthetic agent for laparoscopic sterilization: the effects of premedication on the frequency of adverse clinical reactions. Br J Anaesth 1977; 49: 1159–65

18.	Figallo EM, McKenzie R, Tantisira B, Wadhwa RK, Sinchioco CS. Anaesthesia for dilatation, evacuation and curettage in outpatients: Comparison of subanaesthetic doses of ketamine and sodium methohexitone-nitrous oxide anaesthesia. Can Anaesth Soc J 1977; 24: 110–7

19.	Latto IP, Waldron BA. Anasthesia for caesarean section: Analysis of blood concentrations of halothane using 0.2% or 0.65% halothane with 50% nitrous oxide in oxygen. Br J Anaesth 1977; 49: 371–8

20.	Tammisto T, Tigerstedt I. The need for fentanyl supplementation of N_2_O‐O_2_-relaxant anaesthesia in chronic alcoholics. Acta Anaesthesiol Scand 1977; 21: 216–21

21.	Tammisto T, Tigerstedt I. The need for halothane supplementation of N_2_O‐O_2_‐relaxant anaesthesia in chronic alcoholics. Acta Anaesthesiol Scand 1977; 21: 17–23

22.	Dunn GL, Houlton PJ, Morison DH, Rajagopalan R. A comparative assessment of alfathesin for use in outpatient anaesthesia. Can Anaesth Soc J 1978; 25: 125–9

23.	Drummond JC, Brebner J, Galloon S, Young PS. A randomized evaluation of the reversal of ketamine by physostigmine. Can J Anesth 1979; 26: 288–95

24.	Mattila MA, Larni HM, Nummi SE, Pekkola PO. Effect of diazepam on emergence from ketamine anaesthesia. A double-blind study. Anaesthesist 1979; 28: 20–3

25.	White PF, Ham J, Way WL, Trevor AJ. Pharmacology of ketamine isomers in surgical patients. Anesthesiology 1980; 52: 231–9

26.	Purdell-Lewis JG, Blair DM, McLeod CA. Studies in fentanyl-supplemented anaesthesia: Awareness and effect of naloxone on early post-operative recovery. Can Anaesth Soc J 1981; 28: 57–61

27.	Dich-Nielsen J, Holasek J. Ketamine as induction agent for caesarean section. Acta Anaesthesiol Scand 1982; 26: 139–42

28.	White PF. Comparative evaluation of intravenous agents for rapid sequence induction—thiopental, ketamine, and midazolam. Anesthesiology; 1982; 57: 279–84

29.	Carli F, Stribley GC, Clark M. Etomidate infusion in thoracic anaesthesia. Anaesthesia 1983; 38: 784–8

30.	Klausen N-O, Wiberg Jørgensen F, Chræmmer-Jørgensen B. Psychomimetic reactions after low-dose ketamine infusion. Br J Anaesth 1983; 55: 297–301

31.	Cartwright PD, Pingel SM. Midazolam and diazepam in ketamine anaesthesia. Anaesthesia 1984; 39: 439–42

32.	Mankowitz E, Downing JW, Brock-Utne JG, Maharaj RJ, Morrell DF. Total intravenous anaesthesia using low-dose ketamine infusion for caesarean section. A comparison with a standard inhalation anaesthetic technique. S Afr Med J 1984; 65: 246–50

33.	Sechzer PH. Dreams with low-dose ketamine in obstetrical patients. Curr Ther Res 1984; 35: 396–404

34.	Crawford JS, Lewis M, Davis P. Maternal and neonatal responses related to the volatile agent used to maintain anaesthesia at caesarean section. Br J Anaesth 1985; 57: 482–7

35.	Iqbal KM, Hussain P, Afzalunnessa B. Study of awareness during noninhalational anaesthesia. Bangladesh Med Res Counc Bull 1985; 11: 8–14

36.	Coad NR, Mills PJ, Verma R, Ramasubramanian R. Evaluation of blood loss during suction termination of pregnancy: ketamine compared with methohexitone. Acta Anaesthesiol Scand 1986; 30: 253–5

37.	Schultetus RR, Hill CR, Dharamraj CM, Banner TE, Berman LS. Wakefulness during cesarean section after anesthetic induction with ketamine, thiopental, or ketamine and thiopental combined. Anesth Analg 1986; 65: 723-8

38.	De Grood PMRM, Mitsukuri S, Van Egmond J, Rutten JMJ, Crul JF. Comparison of etomidate and propofol for anaesthesia in microlaryngeal surgery. Anaesthesia 1987; 42: 366–72

39.	Moore JK, Seymour AH. Awareness during bronchoscopy. Ann R Coll Surg Engl 1987; 69: 45–7

40.	Abu Khalaf A, Takrouri M, Toukan A, Abu Khalaf MA, Amr S. Ketamine hydrochloride as sole anesthetic for open liver biopsy. Middle East J Anaesthesiol 1988; 9: 537–43

41.	Galletly DC, Short TG. Total intravenous anaesthesia using propofol infusion — 50 consecutive cases. Anaesth Intensive Care 1988; 16: 150–7

42.	Ghaly RG, Flynn RJ, Moore J. Isoflurane as an alternative to halothane for caesarean section. Anaesthesia 1988; 43: 5–7

43.	Gunawardene RD. Propofol and dreaming. Anaesthesia 1989; 44: 266–7

44.	Bogod DG, Orton JK, Yau HM, Oh TE. Detecting awareness during general anaesthetic caesarean section: An evaluation of two methods. Anaesthesia 1990; 45: 279–84

45.	Eger EI, Lampe GH, Wauk LZ, Whitendale P, Donegan JH. Clinical pharmacology of nitrous oxide: An argument for its continued use. Anesth Analg 1990; 71: 575–85

46.	Piggott SE, Bogod DG, Rosen M, Rees GAD, Harmer M. Isoflurane with either 100% oxygen or 50% nitrous oxide in oxygen for caesarean section. Br J Anaesth 1990; 65: 325–9

47.	Restall J, Johnston IG, Robinson DN. Flumazenil in ketamine and midazolam anaesthesia. Anaesthesia 1990; 45: 938–40

48.	Guit JBM, Koning HM, Coster ML, Niemeijer RPE, Mackie DP. Ketamine as analgesic for total intravenous anaesthesia with propofol. Anaesthesia 1991; 46: 24–7

49.	Liu WHD, Thorp TAS, Graham SG, Aitkenhead AR. Incidence of awareness with recall during general anaesthesia. Anaesthesia 1991; 46: 435–7

50.	Lyons G, Macdonald R. Awareness during caesarean section. Anaesthesia 1991; 46: 62–4

51.	Marsch SC, Schaefer HG, Tschan C, Meier B. Dreaming and anaesthesia: total i.v. anaesthesia with propofol versus balanced volatile anaesthesia with enflurane. Eur J Anaesthesiol 1992; 9: 331–3

52.	Jakobsson J, Oddby E, Rane K. Patient evaluation of four different combinations of intravenous anaesthetics for short outpatient procedures. Anaesthesia 1993; 48: 1005–7

53.	Oddby‐Muhrbeck E, Jakobsson J. Recall of music: a comparison between anaesthesia with propofol and isoflurane. Acta Anaesthesiol Scand 1993; 37: 33–7

54.	Krissel J, Dick WF, Leyser KH, Gervais H, Brockerhoff P, Schranz D. Thiopentone, thiopentone/ketamine, and ketamine for induction of anaesthesia in caesarean section. Eur J Anaesthesiol 1994; 11: 115–22

55.	Liou CM, Kang HM, Lai HC, et al. Will epidural with light general anesthesia increase the incidence of awareness with recall or dream postoperatively. Acta Anaesthesiol Sin 1994; 32: 229–36

56.	Oxorn D, Orser B, Ferris LE, Harrington E. Propofol and thiopental anesthesia: A comparison of the incidence of dreams and perioperative mood alterations. Anesth Analg 1994; 79: 553–7

57.	Suárez M, Mendoza CU, Castañeda R, Lux E, Rufino C, Sánchez R. Incidence of memory of transoperative events during total intravenous general anesthesia with propofol. Arch Med Res 1994; 25: 413–7

58.	Dutton RC, Smith WD, Smith NT. Brief wakeful response to command indicates wakefulness with suppression of memory formation during surgical anesthesia. J Clin Monitor Comput 1995; 11: 41–6

59.	Adesunkanmi ARK. Where there is no anaesthetist: A study of 282 consecutive patients using intravenous, spinal and local infiltration anaesthetic techniques. Trop Doct 1997; 27: 79–82

60.	Brandner B, Blagrove M, McCallum G, Bromley LM. Dreams, images and emotions associated with propofol anaesthesia. Anaesthesia 1997; 52: 750–5

61.	Da Silva JM, Mapleson WW, Vickers MD. Quantitative study of Lowe’s square-root-of-time method of closed-system anaesthesia. Br J Anaesth 1997; 79: 103–12

62.	Kee WDN, Khaw KS, Ma ML, Mainland P-A, Gin T. Postoperative analgesic requirement after cesarean section: A comparison of anesthetic induction with ketamine or thiopental. Anesth Analg 1997; 85: 1294

63.	Nordström O, Engström AM, Persson S, Sandin R. Incidence of awareness in total i.v. anaesthesia based on propofol, alfentanil and neuromuscular blockade. Acta Anaesthesiol Scand 1997; 41: 978–84

64.	Oxorn DC, Ferris LE, Harrington E, Orser BA. The effects of midazolam on propofol-induced anesthesia: propofol dose requirements, mood profiles, and perioperative dreams. Anesth Analg 1997; 85: 553–9

65.	Ranta SO, Laurila R, Saario J, Ali-Melkkilä T, Hynynen M. Awareness with recall during general anesthesia: incidence and risk factors. Anesth Analg 1998; 86: 1084–9

66.	Gray C, Swinhoe CF, Myint Y, Mason D. Target controlled infusion of ketamine as analgesia for TIVA with propofol. Can J Anesth 1999; 46: 957–61

67.	Kudoh A, Matsuki A. Middle latency auditory evoked potentials during total intravenous anesthesia with droperidol, ketamine and fentanyl. Clin Electroencephalogr 1999; 30: 79–83

68.	Yeh FC, Hsu CS, So EC, Chan YF, Chen JY, Shieh JP. Low dose ketamine and midazolam as supplements for spinal anesthesia. Acta Anaesthesiol Sin 1999; 37: 15–9

69.	Chudnofsky CR, Weber JE, Stoyanoff PJ, et al. A combination of midazolam and ketamine for procedural sedation and analgesia in adult emergency department patients. Acad Emerg Med 2000; 7: 228–35

70.	Ghoneim MM, Block RI, Dhanaraj VJ, Todd MM, Choi WW, Brown CK. Auditory evoked responses and learning and awareness during general anesthesia. Acta Anaesthesiol Scand 2000; 44: 133–43

71.	Grace RF, Lesteour T, Sala T, Stewart J. A randomized comparison of low-dose ketamine and lignocaine infiltration with ketamine-diazepam anaesthesia for post-partum tubal ligation in Vanuatu. Anaesth Intensive Care 2001; 29: 30–3

72.	Aceto P, Valente A, Adducci E, Gorgoglione M, De Cosmo G. Implicit memory, dream and auditory evoked responses during anaesthesia. Acta Medica Romana 2002; 40: 21–9

73.	Chaiwat O, Vacharaksa K, Prakanrattana U. Awareness under general anesthesia. J Med Assoc Thai 2002; 85 Suppl 3: S948-955

74.	Vallejo MC, Romeo RC, Davis DJ, Ramanathan S. Propofol-ketamine versus propofol-fentanyl for outpatient laparoscopy: Comparison of postoperative nausea, emesis, analgesia, and recovery. J Clin Anesth 2002; 14: 426–31

75.	Yeo SN, Lo WK. Bispectral index in assessment of adequacy of general anaesthesia for lower segment caesarean section. Anaesth Intensive Care 2002; 30: 36–40

76.	Aceto P, Valente A, Gorgoglione M, Adducci E, De Cosmo G. Relationship between awareness and middle latency auditory evoked responses during surgical anaesthesia. Br J Anaesth 2003; 90: 630–5

77.	Grace RF. The effect of variable-dose diazepam on dreaming and emergence phenomena in 400 cases of ketamine-fentanyl anaesthesia. Anaesthesia 2003; 58: 904–10

78.	Hellwagner K, Holzer A, Gustorff B, et al. Recollection of dreams after short general anaesthesia: influence on patient anxiety and satisfaction. Eur J Anaesthesiol 2003; 20: 282–8

79.	Iwama H, Obara S, Ozawa S, et al. A survey of combined epidural-propofol anesthesia with noninvasive positive pressure ventilation as a minimally invasive anesthetic protocol. Med Sci Monit 2003; 9: CR316–23

80.	Kudoh A, Takase H, Takazawa T. A comparison of anesthetic quality in propofol-spinal anesthesia and propofol-fentanyl anesthesia for total knee arthroplasty in elderly patients. J Clin Anesth 2004; 16: 405–10

81.	Kwok RFK, Lim J, Chan MTV, Gin T, Chiu WKY. Preoperative ketamine improves postoperative analgesia after gynecologic laparoscopic surgery. Anesth Analg 2004; 1044–9

82.	Myles PS, Leslie K, McNeil J, Forbes A, Chan MTV. Bispectral index monitoring to prevent awareness during anaesthesia: the B-Aware randomised controlled trial. The Lancet 2004; 363: 1757–63

83.	Sebel PS, Bowdle TA, Ghoneim MM, et al. The incidence of awareness during anesthesia: A multicenter United States study. Anesth Analg 2004; 99: 833

84.	Wanna O, Werawatganon T, Piriyakitphaiboon S, Taesiri B. A comparison of propofol and ketamine as induction agents for cesarean section. J Med Assoc Thai 2004; 87: 774–9

85.	Leslie K, Myles PS, Forbes A, Chan MTV, Swallow SK, Short TG. Dreaming during anaesthesia in patients at high risk of awareness. Anaesthesia 2005; 60: 239–44

86.	Aceto P, Congedo E, Lai C, Valente A, Gualtieri E, De Cosmo G. Dreams recall and auditory evoked potentials during propofol anaesthesia. NeuroReport 2007; 18: 823–6

87.	Leslie K, Skrzypek H, Paech MJ, Kurowski I, Whybrow T. Dreaming during anesthesia and anesthetic depth in elective surgery patients: a prospective cohort study. Anesthesiology 2007; 106: 33–42

88.	Toscano A, Pancaro C, Peduto VA. Scopolamine prevents dreams during general anesthesia. Anesthesiology 2007; 106: 952–5

89.	Errando CL, Sigl JC, Robles M, et al. Awareness with recall during general anaesthesia: a prospective observational evaluation of 4001 patients. Br J Anaesth 2008; 101: 178–85

90.	Samuelsson P, Brudin L, Sandin RH. BIS does not predict dreams reported after anaesthesia. Acta Anaesthesiol Scand 2008; 52: 810–4

91.	Samuelsson P, Brudin L, Sandin RH. Intraoperative dreams reported after general anaesthesia are not early interpretations of delayed awareness. Acta Anaesthesiol Scand 2008; 52: 805–9

92.	Stait ML, Leslie K, Bailey R. Dreaming and recall during sedation for colonoscopy. Anaesth Intensive Care 2008; 36: 685–90

93.	Bejjani G, Lequeux P-Y, Schmartz D, Engelman E, Barvais L. No evidence of memory processing during propofol-remifentanil target-controlled infusion anesthesia with bispectral index monitoring in cardiac surgery. J Cardiothorac Vasc Anesth 2009; 23: 175–81

94.	Eer AS, Padmanabhan U, Leslie K. Propofol dose and incidence of dreaming during sedation. Eur J Anaesth 2009; 26: 833–6

95.	Leslie K, Sleigh J, Paech MJ, Voss L, Lim CW, Sleigh C. Dreaming and electroencephalographic changes during anesthesia maintained with propofol or desflurane. Anesthesiology 2009; 111: 547–55

96.	Padmanabhan U, Leslie K, Eer AS, Maruff P, Silbert BS. Early cognitive impairment after sedation for colonoscopy: The effect of adding midazolam and/or fentanyl to propofol. Anesth Analg 2009; 109: 1448–55

97.	Paech MJ, Scott KL, Clavisi O, Chua S, McDonnell N. A prospective study of awareness and recall associated with general anaesthesia for caesarean section. Int J Obstet Anesth 2009; 17: 298–303

98.	Xu L, Wu A ‐S., Yue Y. The incidence of intra‐operative awareness during general anesthesia in China: a multi‐center observational study. Acta Anaesthesiol Scand 2009; 53: 873–82

99.	Cheong SH, Lee KM, Lim SH, et al. The effect of suggestion on unpleasant dreams induced by ketamine administration. Anesth Analg 2011; 112: 1082

100.	Kim D-K, Joo Y, Sung T-Y, Kim S-Y, Shin H-Y. Dreaming in sedation during spinal anesthesia: a comparison of propofol and midazolam infusion. Anesth Analg 2011; 112: 1076–81

101.	Zhang C, Xu L, Ma Y, et al. Bispectral index monitoring prevent awareness during total intravenous anesthesia: a prospective, randomized, double-blinded, multi-center controlled trial. Chin Med J 2011; 124: 3664

102.	Xu G, Liu X, Chen J, Zhang J, Wang K. First-trimester surgical abortion increasing the incidence of dreaming: Influence on patient satisfaction during propofol short sedation. Neurosurg Q 2012; 22: 261

103.	Xu GH, Liu XS, Yu FQ, Gu EW, Zhang J, Wang K. Dreaming during sevoflurane or propofol short-term sedation: a randomised controlled trial. Anaesth Intensive Care 2012; 40: 505–10

104.	Aceto P, Lai C, Perilli V, et al. Stress-related biomarkers of dream recall and implicit memory under anaesthesia. Anaesthesia 2013; 68: 1141–7

105.	Aktas G, Sahin E, Turkay Aydogmus M, Erkin Y. The assessment of memory under total intravenous anesthesia. Braz J Anesthesiol 2013; 63: 301–6

106.	Czarko K, Kwiatosz-Muc M, Fijałkowska A, Kowalczyk M, Rutyna R. Intraoperative awareness--comparison of its incidence in women undergoing general anaesthesia for caesarean section and for gynaecological procedures. Anaesthesiol Intensive Ther 2013; 45: 200–4

107.	Matsuyama T, Iranami H, Fujii K, Hirayama M, Kawashima K. Is recall of dreaming during anesthesia a sign of occurrence of postoperative nausea and vomiting? J Anesth 2013; 27: 945–8

108.	Russell IF. The ability of bispectral index to detect intra‐operative wakefulness during isoflurane/air anaesthesia, compared with the isolated forearm technique. Anaesthesia 2013; 68: 1010–20

109.	Xu G, Liu X, Sheng Q, Yu F, Wang K. Sex differences in dreaming during short propofol sedation for upper gastrointestinal endoscopy. Neuroreport 2013; 24: 797–802

110.	Tezcan AH, Ornek DH, Ozlu O, et al. Abuse potential assessment of propofol by its subjective effects after sedation. Pak J Med Sci 2014; 30: 1247–52

111.	Aceto P, Perilli V, Lai C, et al. Minimum alveolar concentration threshold of sevoflurane for postoperative dream recall. Minerva Anestesiol 2015; 81: 1201–9

112.	Gyulaházi J, Varga K, Iglói E, Redl P, Kormos J, Fülesdi B. The effect of preoperative suggestions on perioperative dreams and dream recalls after administration of different general anesthetic combinations: a randomized trial in maxillofacial surgery. BMC Anesthesiol 2015; 15: 11

113.	Chikkahanumanthappa NB, Dasara SG, Pawar SD, Takkalki VV. A comparative study of sedation with dexmedetomidine or midazolam during spinal anesthesia. APICARE 2016; 20

114.	Gyulaházi J, Redl P, Karányi Z, Varga K, Fülesdi B. Dreaming under anesthesia: is it a real possiblity? Investigation of the effect of preoperative imagination on the quality of postoperative dream recalls. BMC Anesthesiology 2016; 16: 53

115.	Kabukcu HK, Sahin N, Ozkaloglu K, Golbasi I, Titiz TA. Bispectral index monitoring in patients undergoing open heart surgery. Braz J Cardiovasc Surg 2016. Available from: http://www.gnresearch.org/doi/10.5935/1678-9741.20160038

116.	Cascella M, Fusco R, Caliendo D, et al. Anesthetic dreaming, anesthesia awareness and patient satisfaction after deep sedation with propofol target controlled infusion: A prospective cohort study of patients undergoing day case breast surgery. Oncotarget 2017; 8: 79248–56

117.	Kundra TS, Kaur P, Nagaraja PS, Manjunatha N. To evaluate dexmedetomidine as an additive to propofol for sedation for elective cardioversion in a cardiac intensive care unit: A double-blind randomized controlled trial. Ann Card Anaesth 2017; 20: 337–40

118.	Villegas S, Suarez S, Owuor J, et al. Intraoperative awareness and experience with a ketamine-based anaesthesia package to support emergency and essential surgery when no anaesthetist is available. Afr J Emerg Med 2019; 9: S56–60

119.	Chen L, Zhang J, He W, Liu W. Comparative effects of dexmedetomidine and midazolam on dreaming of patients undergoing flexible bronchoscopy during general anesthesia. Med Sci Monit 2020; 27 Available from: https://www.medscimonit.com/abstract/index/idArt/929000

120.	Hao L, Hu X, Zhu B, Li W, Huang X, Kang F. Clinical observation of the combined use of propofol and etomidate in painless gastroscopy. Medicine 2020; 99: e23061

121.	Yoshida A, Fujii K, Yoshikawa T, Kawamata T. Factors associated with quality of dreams during general anesthesia: a prospective observational study. J Anesth 2021; 35: 576–80

122.	Alam T, Qureshi R, Aziz M, Khan A. Incidence of accidental awareness during general anesthesia — a tertiary cancer care hospital experience. J Postgrad Med Inst 2022; 36: 3–8

123.	Chen X, Xin D, Xu G, Zhao J, Lv Q. The efficacy and safety of remimazolam tosilate versus dexmedetomidine in outpatients undergoing flexible bronchoscopy: A prospective, randomized, blind, non-inferiority trial. Front Pharmacol 2022; 13: 902065

124.	Chow HS, Hack LM, Kawai M, Heifets BD. Anesthetic-induced intraoperative dream associated with remission of a psychiatric disorder: A case report. A A Pract 2022; 16: e01613

125.	Ghanaie MM, Hosseinzadeh F, Soltanipour S, et al. Intraoperative awareness during cesarean delivery under general anesthesia. Caspian J Neurol Sci 2022; 8: 21–221

126.	Imantalab V, Mohammadzadeh Jouryabi A, Sedighinejad A, et al. Intraoperative awareness in patients undergoing cardiac surgery in an academic center in the north of Iran. Anesth Pain Med 2022; 12 Available from: https://brieflands.com/journals/aapm/articles/131795

127.	Matus H, Kvolik S, Rakipovic A, Borzan V. Bispectral index monitoring and observer rating scale correlate with dreaming during propofol anesthesia for gastrointestinal endoscopies. Medicina 2022; 58: 62

128.	Schäfer B, Klose P, Wiltfang J, Fudickar A. Incidence of dreaming and subjective quality of general anesthesia with and without standardized stress relaxation and dream suggestion by the anesthesiologist: A prospective interventional randomized trial using an adaptive design. Dreaming 2023; 33: 264–74

129.	Dikkala DB, Vamshi Yaswanth, Guntreddy AS. Clinical effectiveness of bispectral index to reduce the risk of awareness for elective surgical patients under general anesthesia: An observational study. Asian J Pharm Clin Res 2023; 212–5

130.	Zhao K, Yang N, Yue J, et al. Factors associated with euphoria in a large subset of cases using propofol sedation during gastrointestinal endoscopy. Front Psychiatry 2023; 14 Available from: https://www.frontiersin.org/journals/psychiatry/articles/10.3389/fpsyt.2023.1001626/full

131.	McKie DBD, Thorp EA. Awareness and dreaming during anaesthesia in a paediatric hospital. Anaesth Intensive Care 1973; 1: 404–14

132.	Feingold A, MacMahon S. Patient preference for ketamine: A case report of multiple anesthesias. Anesth Analg 1975; 54: 35–7

133.	Elliott E, Hanid TK, Arthur LJ, Kay B. Ketamine anaesthesia for medical procedures in children. Arch Dis Child 1976; 51: 56–9

134.	Krantz EM. Low-dose intramuscular ketamine and hyaluronidase for induction of anaesthesia in non-premedicated children. S Afr Med J 1980; 58: 161–2

135.	Hobbs AJ, Bush GH, Downham DY. Peri‐operative dreaming and awareness in children. Anaesthesia 1988; 43: 560–2

136.	O’Sullivan EP, Childs D, Bush GH. Peri‐operative dreaming in paediatric patients who receive suxamethonium. Anaesthesia 1988; 43: 104–6

137.	Maldini B. Ketamine anesthesia in children with acute burns and scalds. Acta Anaesthesiol Scand 1996; 40: 1108–11

138.	Tamminga RYJ, Noordhoek M, Kroon J, Faber-Nijholt R. Ketamine anesthesia with or without diazepam premedication for bone marrow punctures in children with acute lymphoblastic leukemia. Pediatr Hematol Oncol 2000; 17: 383–8

139.	Wathen JE, Roback MG, Mackenzie T, Bothner JP. Does midazolam alter the clinical effects of intravenous ketamine sedation in children? A double-blind, randomized, controlled, emergency department trial. Ann Emerg Med; 2000; 36: 579–88

140.	Huang GH, Davidson AJ, Stargatt R. Dreaming during anaesthesia in children: incidence, nature and associations. Anaesthesia 2005; 60: 854–61

141.	Da Silva PSL, De Aguiar VE, Waisberg DR, Passos RMA, Park MVF. Use of Ketofol for procedural sedation and analgesia in children with hematological diseases. Pediatr Int 2011; 53: 62–7

142.	Wilson RD, Allen CR, Creson D, Schmitt R, Barratt E, Traber DL. Operant behavior and neurophysiological observations of normal human volunteers receiving CL-1848C, a new dissociative anesthetic. Tex Rep Biol Med 1973; 31: 239–48

143.	Morse Z, Sano K, Kanri T. Effects of a midazolam-ketamine admixture in human volunteers. Anesth Prog 2004; 51: 76–9

144.	Noreika V, Jylhänkangas L, Móró L, et al. Consciousness lost and found: subjective experiences in an unresponsive state. Brain Cogn 2011; 77: 327–34

145.	Sarasso S, Boly M, Napolitani M, et al. Consciousness and complexity during unresponsiveness induced by propofol, xenon, and ketamine. Curr Biol 2015; 25: 3099–105

146.	Bonhomme V, Vanhaudenhuyse A, Demertzi A, et al. Resting-state network-specific breakdown of functional connectivity during ketamine alteration of consciousness in volunteers. Anesthesiology 2016; 125: 873–88

147.	Darracq M, Funk CM, Polyakov D, et al. Evoked alpha power is reduced in disconnected consciousness during sleep and anesthesia. Sci Rep 2018; 8: 16664

148.	Radek L, Kallionpää RE, Karvonen M, et al. Dreaming and awareness during dexmedetomidine- and propofol-induced unresponsiveness. Br J Anaesth 2018; 121: 260–9

149.	Colombo MA, Napolitani M, Boly M, et al. The spectral exponent of the resting EEG indexes the presence of consciousness during unresponsiveness induced by propofol, xenon, and ketamine. Neuroimage 2019; 189: 631–44

150.	Radek L, Koskinen L, Sandman N, et al. On no man’s land: Subjective experiences during unresponsive and responsive sedative states induced by four different anesthetic agents. Conscious Cogn 2021; 96: 103239

151.	Scheinin A, Kantonen O, Alkire M, et al. Foundations of human consciousness: Imaging the twilight zone. J Neurosci 2021; 41: 1769–78

152.	Casey CP, Tanabe S, Farahbakhsh Z, et al. Distinct EEG signatures differentiate unconsciousness and disconnection during anaesthesia and sleep. Br J Anaesth 2022; 128: 1006–18

153.	Casey CP, Tanabe S, Farahbakhsh Z, et al. Dynamic causal modelling of auditory surprise during disconnected consciousness: The role of feedback connectivity. NeuroImage 2022; 263: 119657

154.	Guay CS, Hight D, Gupta G, et al. Breathe–squeeze: pharmacodynamics of a stimulus-free behavioural paradigm to track conscious states during sedation. Br J Anaesth 2023; 130: 557–66

155.	Kantonen O, Laaksonen L, Alkire M, et al. Decreased thalamic activity is a correlate for disconnectedness during anesthesia with propofol, dexmedetomidine and sevoflurane but not S-ketamine. J Neurosci 2023; 43: 4884–95

156.	Valli K, Radek L, Kallionpää RE, et al. Subjective experiences during dexmedetomidine- or propofol-induced unresponsiveness and non-rapid eye movement sleep in healthy male subjects. Br J Anaesth 2023; 131: 348–59

157.	Casey CP, Tanabe S, Farahbakhsh ZZ, et al. Evaluation of putative signatures of consciousness using specific definitions of responsiveness, connectedness, and consciousness. Br J Anaesth 2024; 132: 300–11
