# Supplementary material for: Dreaming during anaesthesia: a scoping review
Source: Br J Anaesth. 2026 Jun 11;137(2):506–24. doi: 10.1016/j.bja.2026.05.002 (PMC13494102; doi:10.1016/j.bja.2026.05.002)
Supplement: Supplementary file 5 [file mmc5.docx]

**Table S1**. Emotional valence and content of anaesthesia dreams.

| **Study**  **(Author, Year)** | **Induction**  **aanaesthetics** | **Maintenance anaesthetics** | **Dream valence** | **Frequency of Nightmares** | **Dream Content** |
| --- | --- | --- | --- | --- | --- |
| Clinical Studies on Adults | | | | | |
| Smith (1921)^1^ | Ether | ꟷ | unpleasant | Not mentioned explicitly, but seems like it was a nightmare | A fierce and violent competition, patient’s tongue stuck in gold, agony because of the pain caused by trying to free the tongue (the patient was undergoing tonsil and adenoid removal so the dream may have been related to the pain experienced during surgery) |
| Brice et al. (1970)^2^ | Thiopentone sodium | N₂O/O₂ | 12% unpleasant.  (Pleasant not specified, although it seems that there were also pleasant dreams) | ꟷ | Everyday/concrete situations: Social events, familiar settings (e.g., a party at a pub, often involving alcohol).  Medical contexts: References to prior anaesthesia experiences.  Abstract/philosophical content: Dreams exploring existential or spiritual topics (e.g., conversations with a deity, reflections on death or the universe).  Religious themes: Encounters with divine figures or sacred experiences.  Communication difficulty: Patients often struggled to articulate the abstract or spiritual aspects of their dreams. |
| Harris et al. (1971)^3^ | Group 1 Thiopentone sodium  Group 2 Thiopentone sodium + Morphine before induction  Group 3: Thiopentone sodium  Group 4: Thiopentone sodium | Group 1:  N₂O/O₂  Group 2 (Premedication with Morphine): N₂O/O₂  Group 3: Halothane (0.3-0.5%)  Group 4: Methoxyflurane (0.1-0.3%) | Across all groups:  23% (7/31) pleasant  16% (5/31) unpleasant  (others not specified) | ꟷ | Pleasant social scenarios: Social interactions, often involving alcohol (e.g., pubs, wine lodges).  Unpleasant physical sensations: Bodily experiences possibly linked to surgery, but not indicative of awareness.  Transcendental/spiritual content: Encounters with divine figures, reflections on good and evil.  Auditory elements: Hearing voices or noises.  Medical contexts: Recollections of previous surgeries.  Personal relationships: Conversations with relatives.  Travel imagery: Visits to foreign or unfamiliar places. |
| Crawford & Davies (1975)^4^ | Group 1 and 2: Thiopentone | Group 1: N₂O/O₂ + Methoxyflurane (0.1%) | Group 1:  50% (2/4) pleasant  50% (2/4) unpleasant | ꟷ | ꟷ |
|  |  | Group 2: N₂O/O₂ + Trichloroethylene (0.1%) | Group 2:  80% (4/5) pleasant  20% (1/5) unpleasant | ꟷ | ꟷ |
| Hejja & Galloon (1975)^5^ | Ketamine | Ketamine | 67% (35/52) pleasant 33% (17/52) unpleasant | ꟷ | Dreams were mostly visual (26%ꟷ36%) and olfactory (30%ꟷ38%), less auditory (4ꟷ14%) and tactile (2%) |
| Wilson et al. (1975)^6^ | N₂O/O₂: 100%  Halothane: 64% Narcotics: 19% Enflurane: 9% Innwar@: 4% Ketamine: 2% N₂O/O₂ - + relaxant: 0.2% Other: 1.8% | | 50% (17/34) pleasant or not unpleasant  50% (17/34) unpleasant | ꟷ | Pleasant dreams: Spending time with family, being outdoors, on a vacation/travelling, work or school, hospital- or surgery-related dreams.  Unpleasant dreams: Family member in danger, accidents, illness and death-related themes, people arguing or fighting, late for work. |
| Abouleish & Taylor (1976)^7^ | Thiamylal | N₂O/O₂ | 75% (2/3) pleasant  25% (1/3) unpleasant | ꟷ | ꟷ |
| Crawford et al. (1976)^8^ | Thiopentone | Methoxyflurane (0.1% or 0.2%) or Trichloroethylene (0.1% or 0.2%) + N₂O/O₂ | 0.1 % methoxyflurane: 40% (2/5) pleasant  60% (3/5) unpleasant  0.1% trichloroethylene: 80% (4/5) pleasant  20% (1/5) unpleasant  0.2% methoxyflurane: 83% (5/6) pleasant  17% (1/6) unpleasant  0.2% trichloroethylene: 75% (3/4) pleasant  25% (1/4) unpleasant | ꟷ | ꟷ |
|  |  |  |  | ꟷ | ꟷ |
|  |  |  |  | ꟷ | ꟷ |
|  |  |  |  | ꟷ | ꟷ |
| Downing et al. (1976)^9^ | Ketamine | N₂O/O₂  before delivery, followed by the addition of Halothane (0.5%) or Enflurane (0.6%) after delivery | 67% (10/15) pleasant  33% (5/15) unpleasant | ꟷ | ꟷ |
| Frederickson et al. (1976)^10^ | Etoxadrol | Etoxadrol;  11 patients received also N₂O/O₂ | 71% (15/21) pleasant  29% (6/21) unpleasant | ꟷ | Most commonly included comments such as "floating", "puffy", and "out of this world". |
| Freuchen et al. (1976)^11^ | Group A: Ketamine + flunitrazepam | Group A and B: Ketamine | Group A:  100% (1/1) pleasant | Group A:  0% (0/36); | ꟷ |
|  | Group B: Ketamine + placebo |  | Group B:  44.4% (16/36) pleasant 55.6% (20/36) unpleasant | Group B:  33.3% (12/36) | ꟷ |
| Mahomedy et al. (1976)^12^ | Ketamine | N₂O/O₂ | 33% (5/15) unpleasant | ꟷ | ꟷ |
| Ellingson et al. (1977)^13^ | Group 1: Ketamine | Group 1: Ketamine (as needed) | Group 1:  30% (3/10) pleasant  70% (7/10) unpleasant | ꟷ | Across groups: Included visual disturbances and sensations such as a rotating room and the presence of many people. One patient had the feeling that her child was a rabbit. |
|  | Group 2: Diazepam | Group 2: N₂O/O₂ | Group 2: no dreams | ꟷ |  |
| Figallo, Casali et al. (1977)^14^ | Ketamine | Ketamine | 34.8% (16/46) unpleasant | ꟷ | ꟷ |
| Figallo, McKenzie et al. (1977)^15^ | Group 1: Methohexitone | Group 1: N_2_O/O_2_ | Group 1:  0% (0/3) unpleasant | ꟷ | ꟷ |
|  | Group 2: Ketamine | Group 2: Ketamine i.v. (fractional doses) | Group 2:  100% (6/6) unpleasant | ꟷ | ꟷ |
| Latto & Waldron (1977)^16^ | Sodium Thiopentone | Group 1: Halothane (0.65%) + N₂O/O₂  Group 2: Halothane (0.2%) + N₂O/O₂ | Across both groups:  0% unpleasant | 0% | ꟷ |
| Tammisto & Tigerstedt (1977^17^) | Thiopental | Thiopental + Halothane (0.5%) + N₂O/O₂ | 67% (2/3) unpleasant | 1.8% | ꟷ |
| Drummond et al. (1979)^18^ | Ketamine | Ketamine | 50.7% (34/67) unpleasant | ꟷ | ꟷ |
| Mattila et al. (1979)^19^ | Group 1: Ketamine + Diazepam | Group 1 and 2: Ketamine (as needed) | Group 1: 87% (20/23) pleasant 13% (3/23) unpleasant | ꟷ | Across groups: Unreal in character, coloured and with motion. Often the patients felt that they were outside observers. There was no distinct border between dream and reality, rather a "psychic twilight". Absurd dreams were often found pleasant. |
|  | Group 2: Ketamine |  | Group 2: 72% (28/39) pleasant 28% (11/39) unpleasant | ꟷ |  |
| White et al. (1980)^20^ | Group 1: Racemic ketamine (2mg/kg) | Group 1: Ketamine bolus (1mg/kg) as needed | Group 1:  70% pleasant  5% unpleasant | ꟷ | Across groups: 70ꟷ80% stated that the dreams were unlike any they had experienced previously. Many patients described the dreams as a sensation of traveling through space and time while consciousness was dissociated from the physical body. |
|  | Group 2: +Ketamine (1mg/kg) | Group 2: Ketamine bolus (0.5 mg/kg) as needed | Group 2:  63% pleasant  11% unpleasant | ꟷ |  |
|  | Group 3: -Ketamine (3 mg/kg) | Group 3: Ketamine bolus (1.5 mg/kg) as needed | Group 3:  56% pleasant  22% unpleasant | ꟷ |  |
| Dich-Nielsen et al. (1982)^21^ | Ketamine | N₂O/O₂ | 73% (8/11) pleasant 27% (3/11) unpleasant | ꟷ | ꟷ |
| White (1982)^22^ | Group 1: Thiopental | Group 1, 2, 3, and 4: Enflurane (1- 2%) + N₂O/O₂ | Group 1: no information presented | ꟷ | ꟷ |
|  | Group 2: Ketamine |  | Group 2: 20% unpleasant | ꟷ | ꟷ |
|  | Group 3: Midazolam |  | Group 3: no dreams | ꟷ | ꟷ |
|  | Group 4: Ketamine + Midazolam |  | Group 4: 0% unpleasant | ꟷ | ꟷ |
| Klausen et al. (1983)^23^ | Group 1 (Neuroleptanesthesia): Droperidol + Fentanyl | Group 1 (Neuroleptanesthesia): Fentanyl (when necessary) + N₂O/O₂ | Group 1:  100% (2/2) pleasant  0% (0/2) unpleasant | ꟷ | ꟷ |
|  | Group 2: Ketamine | Group 2: Ketamine + N₂O/O₂ | Group 2:  50% (4/8) pleasant  50% (4/8) unpleasant | ꟷ | ꟷ |
| Cartwright & Pingel (1984)^24^ | Group 1: Preinduction with Midazolam, then Ketamine | Group 1 and 2: Additional boluses of Ketamine (if necessary) | Group 1:  25% (2/8) unpleasant | ꟷ | ꟷ |
|  | Group 2: Preinduction with Diazepam, then Ketamine |  | Group 2:  57% (8/14) unpleasant | ꟷ | ꟷ |
| Mankowitz et al. (1984)^25^ | Group 1 and 2: Thiopentone | Group 1: N₂O/O₂ + Enflurane (0.6–0.8%) | Overall:  75% (6/8) pleasant  25% (2/8) unpleasant  Group 1:  100% (2/2) pleasant  0% (0/0) unpleasant | ꟷ | ꟷ |
|  |  | Group 2: N₂O/O₂ + Ketamine | Group 2:  66.7% (4/6) pleasant  33.3% (2/6) unpleasant | ꟷ | ꟷ |
| Sechzer (1984)^26^ | Ketamine  and/or  Thiamylal | N_2_O/O_2_ (in some cases) | Ketamine:  65% (85/130) pleasant  35% (45/130) unpleasant  Non-ketamine:  48% (10/21) pleasant  52% (11/21) unpleasant | ꟷ | Dream content fell into different categories: Floating or twirling in outer space; colourful visions or some moving objects; sounds, music, noise, popping, etc; unpleasant, distorted, going to die etc; miscellaneous. |
| Crawford et al. (1985)^27^ | Thiopentone | N₂O/O₂ + Trichloroethylene (0.2 or 0.3 %) or Halothane (0.2, 0.3, 0.4, or 0.5 %) | Trichloroethylene 0.2 vol.%:  6 unpleasant  Trichloroethylene 0.3 vol.%:  No dreams  Halothane 0.2 vol.%:  4 unpleasant  Halothane 0.3 vol.%:  3 unpleasant  Halothane 0.4 vol.%:  No dreams  Halothane 0.5 vol.%:  No dreams  (since only the number of unpleasant dreams has been reported, it is not possible to know whether all dreams were unpleasant) | ꟷ | ꟷ |
| Schultetus et al. (1986)^28^ | Group 1: Ketamine (1mg/kg) | Group 1, 2, and 3: N₂O/O₂ | Group 1:  No dreams | ꟷ | ꟷ |
|  | Group 2: Thiopental |  | Group 2: 100% (2/2) pleasant | ꟷ | ꟷ |
|  | Group 3: Ketamine + Thiopental |  | Group 3: 100% (2/2) pleasant | ꟷ | ꟷ |
| de Grood et al. (1987)^29^ | Group 1: Propofol | Group 1: Propofol | Group 1:  100 % (1/1) unpleasant | ꟷ | ꟷ |
|  | Group 2: Etomidate | Group 2: Etomidate | Group 2: no dreams | ꟷ | ꟷ |
| Moore & Seymour (1987)^30^ | Thiopentone | Thiopentone (as needed) | 50% (2/4) unpleasant | ꟷ | ꟷ |
| Abu Khalaf et al. (1988)^31^ | Ketamine | Ketamine (as necessary) | 33.3% (2/6) pleasant  33.3% (2/6) unpleasant  33.3% (2/6) neutral | ꟷ | ꟷ |
| Galletly & Short (1988)^32^ | Fentanyl + Propofol | Propofol | 66% (2/3) pleasant | ꟷ | ꟷ |
| Gunawardene (1989)^33^ | Propofol + Enflurane + N₂O/O | | 100% pleasant (12/12) | 0% | Related to family life |
| Restall et al. (1990)^34^ | Midazolam + Ketamine + Alfentanil | Ketamine + Midazolam + Alfentanil | Group 1:  50% (1/2) pleasant  50% (1/2) unpleasant | ꟷ | ꟷ |
|  |  | Ketamine + Midazolam + Alfentanil + Flumazenil post-anaesthesia | Group 2:  53.8% (7/13) pleasant  38.5% (5/13) unpleasant 7.7% (1/13) unspecified | ꟷ | ꟷ |
| Liu et al. (1991)^35^ | Those 9 patients who dreamed received Thiopentone (n = 6), Propofol (n = 2),  or Etomidate (n = 1) | ꟷ | 100% pleasant (9/9) | 0% | ꟷ |
| Lyons & Macdonald (1991)^36^ | Thiopentone | Up to year 1985: Halothane (0.5%) + N₂O/O₂  Since 1985: Isoflurane (1%) + N₂O/O₂ | ꟷ | ꟷ | The most typical dream theme was friends and relatives in pubs and discotheques, and one woman dreamt that she was taking part in a television program called ‘Game for a Laugh’. |
| Marsch et al. (1992)^37^ | Group 1: Propofol + Fentanyl | Group 1: Propofol + Fentanyl (as needed) + oxygen enriched air (40% oxygen) | Group 1:  54% (7/13) very pleasant  31% (4/13) pleasant  15% (2/13) unspecific | 0% | ꟷ |
|  | Group 2: Thiopentone + Fentanyl | Group 2: N_2_O/Enflurane/Oxygen (40%) + Fentanyl (as needed) | Group 2:  67% (2/3) pleasant  33% (1/3) unpleasant | 0% | ꟷ |
| Jakobsson et al. (1993)^38^ | Group 1: Propofol + Ketamine  Group 2: Propofol + Fentanyl  Group 3: Thiopentone + Fentanyl Group 4: Methohexitone + Fentanyl | N₂O/O₂ + additional doses of Propofol, Methohexitone, or Thiopentone, if necessary | Group 1:  89.7% (26/29) pleasant  (not reported for other groups) | ꟷ | ꟷ |
| Oddby-Muhrbeck & Jakobsson (1993)^39^ | Propofol TIVA | Group 1: Propofol TIVA + N₂O/O₂  Group 2: Isoflurane (1.2%) + N₂O/O₂ | 100% (4/4) pleasant | ꟷ | ꟷ |
| Krissel et al. (1994)^40^ | Group 1: Thiopentone | Group 1:  Before delivery: N₂O/O₂ + Enflurane (0.8%)  After delivery: N₂O/O₂+ Enflurane (1–1.5%) | Group 1:  No dreams | ꟷ | ꟷ |
|  | Group 2: Thiopentone + Ketamine | Group 2:  Before delivery:N₂O/O₂ + Enflurane (0.8%)  After delivery: N₂O/O₂+ Enflurane (1–1.5%) | Group 2:  100% (1/1) pleasant | ꟷ | ꟷ |
|  | Group 3: Ketamine | Group 3:  Before delivery:N₂O/O₂ After delivery: N₂O/O₂+ Enflurane (1–1.5%) | Group 3:  100% (3/3) unpleasant | ꟷ | ꟷ |
| Oxorn et al. (1994)^41^ | Group 1: Propofol  Group 2: Thiopental | Group 1: Propofol + N₂O/O₂  Group 2: Thiopental: + N₂O/O₂ | 1h postop: Group 1:  100% (6/6) pleasant  0% (0/6) unpleasant  Group 2:  75% (3/4) pleasant  25% (1/4) unpleasant  24h postop: Group 1:  100% (5/5) pleasant  0% (0/5) unpleasant  Group 2:  60% (3/5) pleasant  40% (2/5) unpleasant | ꟷ | ꟷ |
| Brandner et al (1997)^42^ | Group 1: Propofol (TIVA)  Group 2: Propofol (induction only)  Group 3: Thiopentone | Group 1: Propofol  Group 2: N₂O/O₂+ Isoflurane (1-1.5%)  Group 3: N₂O/O₂+ Isoflurane (1-1.5%) | No specific data reported. However, none of the dreams were unpleasant. Most patients who received propofol reported their dreams as being ‘very pleasant’, something that was not reported by the thiopentone/enflurane group. | ꟷ | 19 out of 24 had no sexual content, the TIVA propofol group had two dreams with sexual content, the propofol induction group had one sexual dream, the thiopentone induction group had two dreams with sexual content.  Most of these dreams were only vaguely related to sex, e.g. two of them were of meeting people in a happy atmosphere. In one dream a male patient reported being with his wife and wanting to have sex with her. |
| Kee et al. (1997)^43^ | Group 1: Ketamine  Group 2: Thiopental | N₂O/O₂+ Isoflurane (0.5%) | 100% (3/3) pleasant | 0% | ꟷ |
| Nordström et al. (1997)^44^ | Alfentanil + Propofol (TIVA) | Propofol (TIVA) + Alfentanil | After the 2nd interview: 18.5% (5/27) unpleasant | ꟷ | ꟷ |
| Oxorn et al. (1997)^45^ | Group 1: (Pre-induction Midazlam) + Propofol  Group 2: (Pre-induction Placebo) + Propofol | Propofol (boluses) + N₂O/O₂ | The vast majority were pleasant, no actual rates reported. | ꟷ | ꟷ |
| Ranta et al. (1998)^46^ | Thiopental or Propofol | N₂O/O₂+ volatile anaesthetic (Isoflurane or Enflurane) + opioids (Fentanyl or alfentanil) | Most dreams were pleasant or neutral.  0.4% of women (2/8 had possible awareness) and 0% of men had unpleasant dreams. | ꟷ | ꟷ |
| Chudnofsky et al. (2000)^47^ | Midazolam + Ketamine | ꟷ | 67% (12/18) pleasant  11% (2/18) unpleasant  17% (3/18) both pleasant and unpleasant  5% (1/18) neither | ꟷ | ꟷ |
| Ghoneim et al. (2000)^48^ | Group 1: Fentanyl + Thiopental    Group 2: Alfentanil +Thiopental  Group 3: Fentanyl + Thiopental    Group 4: Fentanyl + Thiopental | Group 1: N₂O/O₂+ Fentanyl boluses (as needed)  Group 2: Alfentanil infusion + N₂O/O₂  Group 3: N₂O/O₂+ Isoflurane ( 0.3%)+ Fentanyl boluses (as needed)  (4) Group 4: N₂O/O₂+ Isoflurane (0.7%) | Predominantly positive, no actual rates reported. | 0% | Two of the dreams in patients who received the opioid bolus regimen were related to intraoperative events. The remaining dreams were pleasant with predominantly positive emotions. |
| Grace et al. (2001)^49^ | Group 1: Ketamine (+ Diazepam)  Group 2: Ketamine (+ Lignocaine) | Ketamine | Dreams were mostly rated as pleasant: Group 1: 4.0 (SD = 0.6)  Group 2: 3.4 (SD = 0.7)  (Scale: 1 = very  unpleasant, 2 = unpleasant, 3 = neither pleasant nor  unpleasant, 4 = pleasant, 5 = very pleasant) | ꟷ | ꟷ |
| Aceto et al. (2002)^50^ | Thiopental sodium + Fentanyl | Group 1: Sevoflurane + O_2 (_40%)  Group 2: Sevoflurane + N₂O/O₂  Group 3: Isoflurane + O_2 (_40%)  Group 4: Isoflurane + + N₂O/O₂ | 100% (1/1) pleasant | 0% | ꟷ |
| Vallejo et al. (2002)^51^ | Group 1: Propofol + Ketamine | Group 1 and 2: Isoflurane (0.5%–1.5%) + N₂O/O₂ | Group 1:  20% (2/10) unpleasant | ꟷ | ꟷ |
|  | Group 2: Propofol + Fentanyl |  | Group 2:  100% (1/1) pleasant | ꟷ | ꟷ |
| Aceto et al. (2003)^52^ | Group 1, 2, 3, and 4: Thiopental sodium + Fentanyl | Group 1: Sevoflurane + O_2 (_40%) | Group 1: no dreams | 0% | ꟷ |
|  |  | Group 2: Sevoflurane + N₂O/O₂ | Group 2: 100% (1/1) pleasant | 0% | The dream was related to the story played during anaesthesia: "The dream which I had during anaesthesia came to mind. I was surrounded by sounding-depth made of paper-pulp with many fishes and basketfuls of bread. I dreamed I heard your voice which made me feel very relaxed but I don't remember what you said". |
|  |  | Group 3: Isoflurane + O_2 (_40%) | Group 3: no dreams | 0% | ꟷ |
|  |  | Group 4: Isoflurane + + N₂O/O₂ | Group 4: no dreams | 0% | ꟷ |
| Grace (2003)^53^ | (All groups received Fentanyl, followed by 0 mg/kg/min Diazepam, 0.025 mg/kg/min Diazepam, 0.05 mg/kg/min Diazepam, 0.1 mg/kg/min Diazepam, or 0.175 mg/kg/min Diazepam)  + Ketamine (infusion) | ꟷ | The median scores in all the groups were either 4 (pleasant) or 5 (very pleasant). | ꟷ | Dreams typically involved the sensation of floating, flying, seeing flowers, or looking down on one's body from above. |
| Hellwagner et al. (2003)^54^ | Group 1: Propofol + Fentanyl  Group 2: Methohexital-Isoflurane + Fentanyl | Group 1: Propofol  Group 2: Isoflurane (1%) | 30% pleasant  4% unpleasant | ꟷ | The main dream themes: spending time with family; spending time at home or outdoors; being on the beach; pets (dog). No sexual dreams or dreams related to surgery. |
| Iwama et al. (2003)^55^ | Propofol | Propofol | 87% (122/141) good  13% (19/141) usual  0% (0/141) bad | 0% | ꟷ |
| Kudoh et al. (2004)^56^ | Group 1 and 2: Propofol | Group 1: Propofol | Group 1:  98% (47/48) good  2% (1/48) bad | ꟷ | ꟷ |
|  |  | Group 2: Propofol + Fentanyl (given until skin incision) | Group 2:  70% (7/10) good  30% (3/10) bad | ꟷ |  |
| Kwok et al. (2004)^57^ | Propofol and Fentanyl  (Group 1: Ketamine before incision  Group 2: Ketamine after wound closure  Group 3: Placebo group with no Ketamine) | N₂O/O₂ + Isoflurane (0.5%–1%) | No patients complained of bad dreams. | ꟷ | ꟷ |
| Myles et al. (2004)^58^ | Different combinations of:  Propofol, Thiopentone, or Midazolam | Propofol (TIVA) or N_2_O and/or Volatile anaesthetics and/or Fentanyl | ꟷ | ꟷ | Surgery/hospital related; Aliens taking over the operation; Dying; Floating; Talking with a friend; Car headlights; Fishing in a boat that sank during a storm; A nightmare of nurses calling the patient the wrong name, but patient couldn’t talk; Terrible pain and “screaming out”. Some of these considered as possible awareness. |
| Wanna et al. (2004)^59^ | Group 1: Propofol  Group 2: Ketamine | Sevoflurane + N₂O/O₂ | Group 1:  8% good dream  (Rates not reported for Group 2; only mentioned that the good dream incidence was lower) | ꟷ | ꟷ |
| Leslie et al. (2005)^60^ | Anaesthetics were used according to the anaesthetists' discretion:  Propofol, Thiopental | Anaesthetics were used according to the anaesthetists' discretion:  Propofol (TIVA), Inhalational agents (not specified), N_2_O, Fentanyl | ꟷ | ꟷ | Some patients reported dreams about hospital experiences (e.g., “I dreamt that I saw my daughter, who was pregnant, having a Caesarean Section”; “I dreamt that I was having a conversation with my anaesthetist about the research trial. The dream was interrupted by the anaesthetist’s  voice trying to wake me up”), whereas others reported dreams about unrelated subjects (e.g., “I had vivid dreams about rescuing a pup from a tunnel, flying a plane, swimming and being stuck in a boat. I felt restricted in my movements and felt I was gagging”). |
| Aceto et al. (2007)^61^ | Propofol + Remifentanil | Propofol + Remifentanil | Pleasant (no specific rates reported) | ꟷ | Dreams content related to patients’ habitual life with an unusually pleasant atmosphere. |
| Leslie et al. (2007)^62^ | Anaesthetics were used according to the anaesthetists' discretion:  Mostly Propofol, seven patients induced with Sevoflurane and three with Thiopentone bolus | Anaesthetics were used according to the anaesthetists' discretion:  Propofol TCI,  Desflurane, Sevoflurane, N_2_O, Fentanyl, Remifentanil | Mostly pleasant (the median score of emotional valence was 4 on a 5-point scale, where 1 = very negative and 5 = very positive) | ꟷ | Most dreams were pleasant, not strange, and meaningful to the patient (i.e., the dream involved family, friends, work or recreation) Vividness: Mdn = 4 (from 1 = not at all vivid to 5 = most vivid ever) Memorability: Mdn = 3 (from 1 = only  remember dreaming to 5 = most memorable ever) Amont of sound: Mdn = 4 (from 1 = no sound to 5 = most sound ever) Emotional intensity: Mdn = 3 (from 1 = not at all intense to 5 = most intense ever) Meaningfulness: Mdn = 4 (from 1 = not at all meaningful to 5 = most meaningful ever) Amount of movement: Mdn = 4 (from 1 = no movement to 5 = most movement ever) Strangeness: Mdn = 1 (from 1 = not at all strange to 5 = most strange ever) |
| Toscano et al. (2007)^63^ | Propofol  (30 min before procedure, 50 patients received scopolamine and 50 received atropine) | Propofol | All dreams were pleasant or neutral | 0% | The content was not related to the surgery. |
| Errando et al. (2008)^64^ | (1) TIVA: Propofol  (2) Balanced Anaesthesia: Any i.v. induction drug  (3) Mixed Anaesthesia: Any i.v. drug,    (4) Other Anaesthetic Combinations: Various induction methods (e.g., bolus dose of Propofol for short procedures, Midazolam-Ketamine, and Midazolam- Opioids) | (1) TIVA: Propofol  (2) Balanced Anaesthesia: Inhalation anaesthetics  (3) Mixed Anaesthesia: N₂O/O₂  (4) Other Anaesthetic Combinations: Ketamine, Midazolam, Opioids | Most dreams were pleasant (32.4%), with a small percentage being unpleasant (1.9%) or indifferent (1.0%) | ꟷ | ꟷ |
| Samuelsson et al. (2008)^65^ | Thiopentone or Propofol | Sevoflurane or Sevoflurane + N_2_O or Propofol | 90% (189/211) pleasant/neutral  10% (22/211) unpleasant | ꟷ | ꟷ |
| Samuelsson et al. (2008)^66^ | Thiopentone or Propofol | Volatile anaesthetics (no specific agents mentioned) | 77% (178/232) pleasant/neutral  23% (54/232) unpleasant | ꟷ | ꟷ |
| Stait et al. (2008)^67^ | Propofol + Midazolam and/or Fentanyl | Same as induction – intermittent IV bolus, not continuous infusion | Mostly positive (the median score of emotional valence was 4 on a 5-point scale, where 1 = very negative and 5 = very positive) | ꟷ | Dreams were generally short, simple dreams about family, friends, work, and recreation. |
| Eer et al. (2009)^68^ | Group 1: Propofol only  Group 2: Propofol + Midazolam  Group 3: Propofol + Fentanyl  Group 4: Propofol + Fentanyl + Midazolam | | Most of the dreams were pleasant; specific rates are not reported. | ꟷ | Most of the dreams were simple, pleasant dreams about everyday life. Main dream themes: family; home; working; being on the beach. |
| Leslie et al. (2009)^69^ | Fentanyl + Propofol | Group 1: Propofol TCI  Group 2: Desflurane | ꟷ | ꟷ | Patients reported simple dreams about family, friends, work, and recreation. |
| Paech et al. (2009)^70^ | The most common anaesthetics used: Thiopental (83% cases), Propofol (15% cases), Ketamine (1.6% cases) | The most common anaesthetics used: Sevoflurane (63% cases), Isoflurane (28% cases), Desflurane (8.5% cases), Propofol (1.3% cases) N_2_O (69% cases) | 96% (24/25) pleasant/neutral  4% (1/25) unpleasant | ꟷ | People; Noises; Being on an island; Gardens with flowers and birds; Family; Surgery/Hospital; Continuous motion; Being at home; Giving birth/babies |
| Cheong et al. (2011)^71^ | (1. spinal anaesthetic bupivacaine) +  2. i/v Ketamine | | Suggestion group:  54% (27/50) pleasant  46% (23/50) neither pleasant or unpleasant or mixed  0% (0/50) unpleasant | Suggestion group: 0% (0/50) | Neither or mixed: body floating, travelled to a 4-dimensional world, cave or in a maze; saw a strange scene and colour; wandered around;  Quite pleasant: saw the palace; travelled; mountain climbing; auto racing; walking in a flower garden; having fun with my baby Very pleasant: flying; high as a kite |
|  |  |  | No-suggestion group:  22% (11/50) pleasant  70% (35/50) neither pleasant or unpleasant or mixed  8% (4/50) unpleasant | No-suggestion group: 6% (3/50) | Quite unpleasant: family died  Neither or mixed: body floating, travelled to a 4-dimensional world, cave or in a maze; saw a strange scene and colour; wandered around; being in the hospital  Quite pleasant: saw the palace; travelled; mountain climbing; walking in a flower garden; went to the movies;  Very pleasant: flying; high as a kite |
| Kim et al. (2011)^72^ | Group 1: Propofol  Group 2: Midazolam | | Most dreams were pleasant | ꟷ | Pleasant ruminations about everyday life, such as travelling with friends, dreaming about work or planning to do something (n = 36); dreams about the imaginary experiences operation, such as surgery was successful (n = 5); being pursued by someone ( n = 6); others, such as flying, mining gold (n = 4) |
| Xu et al. (2012)^73^ | Propofol + Fentanyl | None | Emotional content: median rating of 3 in both groups (from 1 = very negative to 5 = very positive)  Emotional intensity: median rating of 2 in Group 1 and 3 in Group 2 (from 1 = not at all intense to 5 = most intense ever experienced) | ꟷ | Simple, pleasant, meaningful dreams.  Vividness: Mdn = 4 (Group 1) and Mdn = 3 (Group 2) (from 1 = not at all vivid to 5 = most vivid ever) Memorability: Mdn = 3 (Groups 1 and 2) (from 1 = only remember dreaming to 5 = most memorable ever) Amont of sound: Mdn = 4 (Groups 1 and 2) (from 1 = no sound to 5 = most sound ever) Meaningfulness: Mdn = 4 (Groups 1 and 2) (from 1 = not at all meaningful to 5 = most meaningful ever) Amount of movement: Mdn = 1 (Group 1) and Mdn = 2 (Group 2) (from 1 = no movement to 5 = most movement ever) Strangeness: Mdn = 3 (Group 1 and 2) (from 1 = not at all strange to 5 = most strange ever) |
| Xu et al. (2012)^74^ | Group 1: Propofol (iv bolus)  Group 2: Sevoflurane | Group 1: Propofol (infusion)  Group 2: Sevoflurane | 49% (46/93) positive  10% (9/93) negative  The rest not specified  Emotional content scores: median 3 (on a scale from 1 = very negative to 5 = very positive) for both groups | ꟷ | Most dreams were simple, pleasant, about everyday life, and meaningful.  Vividness: Mdn = 3 (Group 1 and 2) (from 1 = not at all vivid to 5 = most vivid ever) Memorability: Mdn = 3 (Groups 1) and Mdn = 2 (Group 2) (from 1 = only remember dreaming to 5 = most memorable ever) Amont of sound: Mdn = 2 (Group 1) and Mdn : 1 (Group 2) (from 1 = no sound to 5 = most sound ever) Meaningfulness: Mdn = 4 (Groups 1 and 2) (from 1 = not at all meaningful to 5 = most meaningful ever) Amount of movement: Mdn = 3 (Group 1) and Mdn = 1 (Group 2) (from 1 = no movement to 5 = most movement ever) Strangeness: Mdn = 3 (Group 1 and 2) (from 1 = not at all strange to 5 = most strange ever) |
| Aceto et al. (2013)^75^ | Propofol + Remifentanil | Sevoflurane + Remifentanil | Mostly pleasant | ꟷ | Dreams were characterized by positive emotions and contained references to life events. |
| Czarko et al. (2013)^76^ | Group 1: Propofol + Remifentanil (TIVA) | Group 1: Propofol + Remifentanil (TIVA) | Group 1:  14% (1/7) pleasant  0% (0/7) unpleasant  86 (6/7) unidentified | ꟷ | ꟷ |
|  | Group 2: Thiopentone + Fentanyl | Group 2: Sevoflurane  + N₂O/O_2_ | Group 2:  63% (5/8) pleasant  25% (2/8) unpleasant  12% (1/8) unidentified | ꟷ | ꟷ |
|  | Group 3: Propofol + Fentanyl | Group 3: Sevoflurane  + N₂O/O_2_ | Group 3:  100% (2/2) pleasant  0% (0/2) unpleasant  0% (0/2) unidentified | ꟷ | ꟷ |
|  | Group 4: Thiopentone + Fentanyl | Group 4: Sevoflurane  + N₂O/O_2_ | Group 4:  50% (13/26) pleasant  12% (3/26) unpleasant  38% (10/26) unidentified | ꟷ | ꟷ |
| Russell (2013)^77^ | Fentanyl + Propofol | Isoflurane | 100% pleasant | 0% | ꟷ |
| Xu et al. (2013)^78^ | Propofol (bolus) + Fentanyl | Propofol (infusion) | Men:  45% (14/31) positive  6% (2/31) negative  Women:  18% (3/17) positive  29% (5/17) negative  (The rest are not specified) | ꟷ | Men reported dreams that were more vivid, meaningful, familiar, and memorable than those of women (e.g., drinking with good friends). |
| Tezcan et al. (2014)^79^ | Propofol | Propofol (additional injections as needed) | All pleasant (median rating of emotional content 2.9 on a scale from 0-5) | 0% | Simple pleasant dreams. |
| Aceto et al. (2015)^80^ | Propofol + Fentanyl | Sevoflurane | 90% (26/29) pleasant  10% (3/29) negative | ꟷ | Dreams contained life episodes: working, being with family, making a journey. In 3 patients dreams contained fantasy subjects and were always positive. The negative dreams were about not being able to finish a job, not succeeding in passing an exam. |
| Gyulaházi et al. (2015)^81^* | In all 3 groups, 3 subgroups: Subgroup 1: Etomidate  Subgroup 2: Propofol  Subgroup 3: Propofol | In all 3 groups, 3 subgroups: Subgroup 1: Sevoflurane  Subgroup 2: Sevoflurane  Subgroup 3: Propofol (TIVA) | ꟷ | ꟷ | Dreams were related to the suggestion in the suggestion groups. |
| Gyulaházi et al. (2016)^82^* | In all 3 groups, 3 subgroups: Subgroup 1: Etomidate  Subgroup 2: Propofol  Subgroup 3: Propofol | In all 3 groups, 3 subgroups: Subgroup 1: Sevoflurane  Subgroup 2: Sevoflurane  Subgroup 3: Propofol (TIVA) | Control group:  87.5% positive  Suggestion groups:  97.5% positive | ꟷ | Nature (thought or cinematic like):  Control group: 0/16 thought-like, 13/16 cinematic like, 3/16 didn’t remember  Suggestion groups: 15/82 thought-like, 62/82 cinematic like, 5/82 didn’t remember  Quality (colour or black and white):  Control group: 87.5% colourful; 12.5% black and white  Suggestion groups: 85.5% colourful, 14.4% black and white  Participant of the dream (yes/no)  Control group: 100% yes  Suggestion groups: 97.7% yes  Content: 1) loved ones; 2) work; 3) sports; 4) holidays; 5) erotic; 6) fairy tale; 7) religious; 8) other; 9) surgery |
| Cascella et al. (2017)^83^ | Propofol (TCI) | Propofol (TCI) | Predominantly positive emotions; no specific rates reported | ꟷ | Themes reported: swimming, partner, travelling with family, surgery, people from life. |
| Villegas et al. (2019)^84^ | Ketamine | Ketamine | Procedural sedation: 52% (11/21) pleasant  33% (7/21) frightening  10% (2/21) neither  5% (1/21) do not remember  Emergence surgery: 20% (1/5) pleasant  40% (2/5) frightening  0% (0/5) neither  40% (2/5) do not remember | ꟷ | ꟷ |
| Chen et al. (2020)^85^ | Group 1: (Pre-induction Dexmedetomidine) + Fentanyl + Propofol  Group 2: (Pre-induction Midazolam) + Fentanyl + Propofol | Propofol (TIVA) | In both groups most of the dreams were pleasant (median score 4; on a scale from 1 = very unpleasant to 5 = very pleasant) | ꟷ | In both groups dreams contained a little talking and a little action (as assessed on scales measuring these variables) but the memorability of the dream was rated better in the midazolam than dexmedetomidine group |
| Hao et al. (2020)^86^ | Group 1. Propofol | Group 1, 2, 3 and 3: If needed during the procedure, a top-up dose | Group 1:  44% (15/34) good  29.4% (10/34) ordinary 26.5% (9/34) bad | ꟷ | ꟷ |
|  | Group 2: Propofol + Etomidate (10ml) |  | Group 2:  38.5% (5/13) good  38.5% (5/13) ordinary  20% (3/15) bad | ꟷ | ꟷ |
|  | Group 3: Propofol + Etomidate (5ml) |  | Group 3:  38.5% (10/26) good  34.6% (9/26) ordinary 26.9% (7/26) bad | ꟷ | ꟷ |
| Yoshida et al. (2021)^87^ | ꟷ | Propofol + Remifentanil or Sevoflurane + Remifentanil or Desflurane + Remifentanil | 50.2% (147/293) pleasant  47.1% indifferent 2.7% unpleasant | ꟷ | ꟷ |
| Alam et al. (2022)^88^ | Propofol (TIVA) or other i.v. induction agents (not specified) | Propofol (TIVA) or Sevoflurane | 100% (2/2) unpleasant | ꟷ | ꟷ |
| Matus et al. (2022)^89^ | Propofol (TIVA) | Propofol (TIVA) | 98% (51/52) pleasant  2% (1/52) unpleasant | ꟷ | ꟷ |
| Schäfer et al. (2022)^90^ | Propofol + Remifentanil | Remifentanil | Suggestion group:  0% (0/17) unpleasant  Control group:  6% (1/7) unpleasant | ꟷ | The most frequent content: vacations (50%) sports or music (22.5%), being with friends or family (20%) |
| **Clinical Studies on Children and/or Adolescents** | | | | | |
| McKie & Thorp (1973)^91^ | Different anaesthetics and their combinations used: Ketamine, Thiopentone, Halothane, N_2_O/O_2_; Methoxyflurane  (Also included patients who were lightly sedated) | | Details not presented, although patients generally tended to be relieved and not upset, but some dreams were unpleasant | At least 1 nightmare | Family; superman nearly shot; dreamt of being the fastest runner in the school; fell into a creek; was building a house and hit left thumb with a hammer; deep voice; "horrible dream" (no details); surgery-related (being cut open by a big square box; crying and told to be quiet by the nurses; being tied to the railway line with the train coming and being saved by the doctor; “getting needles, stitches, and hospitals”. |
| Feingold & MacMahon (1975)^92^ | 17 anaesthetic procedures: First ten with Halothane + N_2_0 (3 times), Enflurane + N_2_0 (4 times), Fluroxene + N_2_0 (once), Thiopental + N_2_0 (once), Innovar® + N_2_0.  The 11th procedure involved N_2_0 +Thiopental. The next 6 involved N_2_0 + Ketamine. | | The dream during the 11th procedure was very unpleasant | Seems like the dream was a nightmare although not explicitly stated as such | In that unpleasant dream the patient was the last person alive in the universe and was herself dying. |
| Elliott et al. (1976)^93^ | Ketamine hydrochloride | Ketamine | 0% unpleasant | 0% | ꟷ |
| Krantz et al. (1980)^94^ | Ketamine i.m. | N_2_O/O_2_ | 0% unpleasant | 0% | Twelve children between 8 and 13 years old were told immediately after the injection that they would dream during the procedure. They were told they were at a friend's birthday party, were enjoying themselves, eating cookies, drinking cold drinks, and playing football. Six of the 12 children dreamt of the suggested content. |
| Tamminga et al. (2000)^95^ | Ketamine i.v. | Ketamine i.v. | 1/3 unpleasant | ꟷ | ꟷ |
| Wathen et al. (2000)^96^ | Group 1: Ketamine (i.v.) | ꟷ | Mostly pleasant (no further details) | Group 1: 3.1% | ꟷ |
|  | Group 2: Ketamine (i.v.) + Midazolam (i.v.) | ꟷ |  | Group 2: 0% | ꟷ |
| Huang et al. (2005)^97^ | Propofol or Sevoflurane or Halothane or Thiopentone | Isoflurane or Sevoflurane or Halothane or N_2_O or Propofol | Mostly pleasant  10% (9/90) "bad dreams" | Explicitly mentioned at least 1 nightmare (although other “bad dreams” can also be nightmares) | Animals, playing sports, or hospital- or illness-related events, familiar characters (family, friends, fictional figures), fantastical (fairy tale characters) or travel scenarios, food, toys, or unusual sensory experiences, ranging from pleasant to scary. |
| da Silva et al. (2011)^98^ | Ketofol (1:1 Ketamine + Propofol) (i.v.) | Ketofol (i.v.; additional boluses) | 0% unpleasant | 0% | ꟷ |
| Experimental Studies | | | | | |
| Wilson et al. (1973)^99^ | CL-1848C (Etoxradrol; i.v.) | ꟷ | None felt that "this dream process represented a threat to their security and over half of the individuals felt the experience was either extremely interesting or very pleasant" | 0% | Space flight, the supernatural |
| Morse et al. (2004)^100^ | Midazolam + Ketamine (boluses) | Midazolam + Ketamine + Saline (infusion) delivered over 1h at a constant rate | 100% pleasant | ꟷ | ꟷ |
| Noreika et al. (2011)^101^ | All groups: LOR induced by escalating stepwise increases in concentrations (except for more abrupt Xenon sessions).  Group 1: Dexmedetomidine (TCI) | | Group 1:  10% positive  10% negative.  Across 2 sessions:  21% positive  5% negative | ꟷ | Across groups: Some examples: playing football, being a pirate, head coming out of the body; Vacationing; One of the study nurses being suspended from work, being on a beach; Spending time with a friend |
|  | Group 2: Propofol (TCI) | | Group 2:  20% positive  0% negative  Across 2 sessions:  21% positive  0% negative | ꟷ |  |
|  | Group 3: Sevoflurane | | Group 3:  10% positive  10% negative | ꟷ |  |
|  | Group 4: Xenon | | Group 4:  40% positive  40% negative | ꟷ |  |
| Sarasso et al. (2015)^102^ | Group 3: Ketamine (i.v.) | Group 3: Ketamine (i.v. infusion) | ꟷ | ꟷ | (1) many vivid experiences rich in visual and emotional components; (2) an explicit narrative structure; (3) extended in time; and (4) unrelated to the anaesthesia environment |
| Bonhomme et al. (2016)^103^ | Ketamine (computer-controlled i.v. infusion) | | ꟷ | ꟷ | Major themes: sense of well-being, joy and peace; unearthly environment; flying; bright light; daydreaming; sensation of dissolution in the environment/universe; body distortion; falling sensation; imprisonment/oppression sensation; dying sensation |
| Radek et al (2018)^104^** | Group 1: Dexmedetomidine (TCI)  Group 2: Propofol (TCI) | | 20% positive  12% negative | ꟷ | Complexity:  Static - 37.5%  Scenery - 34.4%  Dynamic - 28.1%  Modalities:  Visual - 89.1%  Auditory - 31.1%  Kinaesthetic - 27.8%  Gustatory - 9.1%  Tactile - 9.1%  Interoception - 8.3%  Pain/temperature - 6.1%  Olfactory - 0%  Cognition: 30.1% Out of body experiences: 0% Sense of presence: 2.5% |
| Radek et al. (2021)^105^ | Group 1: Dexmedetomidine (TCI) | | Group 1:  53.8% positive  11.5% negative | ꟷ | Static reports more common than dynamic reports (Group 1: 69.2% static, 23.1% dynamic;  Group 2: 63.6% static, 36.4% dynamic;  Group 3: 25% static, 50% dynamic)  Group 4: 0% static, 100% dynamic) |
|  | Group 2: Propofol (TCI) | | Group 2:  63.6% positive  9.1% negative |  |  |
|  | Group 3: Sevoflurane | | Group 3:  75% positive  0% negative |  |  |
|  | Group 4: S-Ketamine (TCI) | | Group 4:  50% positive  0% negative |  |  |
| Valli et al. (2023)^106^** | Group 1: Dexmedetomidine (TCI)  Group 2: Propofol (TCI) | | 39% positive 33% negative | ꟷ | Complexity: No perceptual content - 9.1% Static - 27.3% Dynamic - 63.6%  Modality: Visual - 90.1% Auditory - 45.5%  Kinaesthetic -25% Interoceptive - 6% Tactile - 3% Nociceptive - 3%  Gustatory - 0% Olfactory - 0%  Cognition: 45.5% Out of body experience: 0% Sense of presence: 6% |

*Note*. Studies sharing the same superscript (*, **) are based on overlapping datasets. TCI = target-controlled infusion.

**References**

1.	Smith LG. Speech defect resulting from ether shock. The Pedagogical Seminary 1921; Available from: https://www.tandfonline.com/doi/abs/10.1080/08919402.1921.10533994

2.	Brice DD, Hetherington RR, Utting JE. A simple study of awareness and dreaming during anaesthesia. Br J Anaesth 1970; 42: 535–42

3.	Harris TJB, Brice DD, Hetherington RR, Utting JE. Dreaming associated with anaesthesia: The influence of morphine premedication and two volatile adjuvants. Br J Anaesth 1971; 43: 172–8

4.	Crawford JS, Davies P. A return to trichloroethylene for obstetric anaesthesia. Br J Anaesth 1975; 47: 482–90

5.	Hejja P, Galloon S. A consideration of ketamine dreams. Can Anaesth Soc J 1975; 22: 100–5

6.	Wilson SL, Vaughan RW, Stephen CR. Awareness, dreams, and hallucinations associated with general anesthesia. Anesth Analg 1975; 54: 609

7.	Abouleish E, Taylor FH. Effect of morphine-diazepam on signs of anesthesia, awareness, and dreams of patients under N20 for cesarean section. Anesth Analg 1976; 55: 702–5

8.	Crawford JS, James FM, Davies P, Crawley M. A further study of general anaesthesia for caesarean section. Br J Anaesth 1976; 48: 661–7

9.	Downing JW, Mahomedy MC, Jeal DE, Allen PJ. Anaesthesia for caesarean section with ketamine. Anaesthesia 1976; 31: 883–92

10.	Frederickson EL, Longnecker DE, Allen GW. Clinical Investigation of a new intravenous anesthetic—etoxadrol hydrochloride (CL-1848C; U-37862A). Anesth Analg 1976; 55: 335-9

11.	Freuchen I, Ostergaard J, Kuhl JB, Mikkelsen BO. Reduction of psychotomimetic side effects of Ketalar® (ketamine) by Rohypnol® (flunitrazepam): A randomized, double‐blind trial. Acta Anaesthesiol Scand 1976; 20: 97–103

12.	Mahomedy, MC, Downing, JW, Jeal DE, Allen PJ. Ketamine for anaesthetic induction at caesarean section. S Afr Med J 1976; 50: 846–8

13.	Ellingson A, Haram K, Sagen N. Ketamine and diazepam as anaesthesia for forceps delivery: A comparative study. Acta Anaesthesiol Scand 1977; 21: 37–40

14.	Figallo EM, Casali H, Mckenzie R, Tantisira B, Wadhwa RK, Taylor FH. Ketamine as the sole anaesthetic agent for laparoscopic sterilization: the effects of premedication on the frequency of adverse clinical reactions. Br J Anaesth 1977; 49: 1159–65

15.	Figallo EM, McKenzie R, Tantisira B, Wadhwa RK, Sinchioco CS. Anaesthesia for dilatation, evacuation and curettage in outpatients: Comparison of subanaesthetic doses of ketamine and sodium methohexitone-nitrous oxide anaesthesia. Can Anaesth Soc J 1977; 24: 110–7

16.	Latto IP, Waldron BA. Anasthesia for caesarean section: Analysis of blood concentrations of halothane using 0.2% or 0.65% halothane with 50% nitrous oxide in oxygen. Br J Anaesth 1977; 49: 371–8

17.	Tammisto T, Tigerstedt I. The need for halothane supplementation of N_2_O‐O_2_‐relaxant anaesthesia in chronic alcoholics. Acta Anaesthesiol Scand 1977; 21: 17–23

18.	Drummond JC, Brebner J, Galloon S, Young PS. A randomized evaluation of the reversal of ketamine by physostigmine. Can J Anesth 1979; 26: 288–95

19.	Mattila MA, Larni HM, Nummi SE, Pekkola PO. Effect of diazepam on emergence from ketamine anaesthesia. A double-blind study. Anaesthesist 1979; 28: 20–3

20.	White PF, Ham J, Way WL, Trevor AJ. Pharmacology of ketamine isomers in surgical patients. Anesthesiology 1980; 52: 231–9

21.	Dich-Nielsen J, Holasek J. Ketamine as induction agent for caesarean section. Acta Anaesthesiol Scand 1982; 26: 139–42

22.	White PF. Comparative Evaluation of intravenous agents for rapid sequence induction—thiopental, ketamine, and midazolam. Anesthesiology 1982; 57: 279–84

23.	Klausen N-O, Wiberg Jørgensen F, Chræmmer-Jørgensen B. Psychomimetic reactions after low-dose ketamine infusion. Br J Anaesth 1983; 55: 297–301

24.	Cartwright PD, Pingel SM. Midazolam and diazepam in ketamine anaesthesia. Anaesthesia 1984; 39: 439–42

25.	Mankowitz E, Downing JW, Brock-Utne JG, Maharaj RJ, Morrell DF. Total intravenous anaesthesia using low-dose ketamine infusion for caesarean section: A comparison with a standard inhalation anaesthetic technique. S Afr MedJ 1984; 65: 246–50

26.	Sechzer PH. Dreams with low-dose ketamine in obstetrical patients. Curr Ther Res 1984; 35: 396–404

27.	Crawford JS, Lewis M, Davis P. Maternal and neonatal responses related to the volatile agent used to maintain anaesthesia at caesarean section. Br J Anaesth 1985; 57: 482–7

28.	Schultetus RR, Hill CR, Dharamraj CM, Banner TE, Berman LS. Wakefulness during cesarean section after anesthetic induction with ketamine, thiopental, or ketamine and thiopental combined. Anesth Analg 1986; 65: 723-8

29.	De Grood PMRM, Mitsukuri S, Van Egmond J, Rutten JMJ, Crul JF. Comparison of etomidate and propofol for anaesthesia in microlaryngeal surgery. Anaesthesia 1987; 42: 366–72

30.	Moore JK, Seymour AH. Awareness during bronchoscopy. Ann R Coll Surg Engl 1987; 69: 45–7

31.	Abu Khalaf A, Takrouri M, Toukan A, Abu Khalaf MA, Amr S. Ketamine hydrochloride as sole anesthetic for open liver biopsy. Middle East J Anaesthesiol 1988; 9: 537–43

32.	Galletly DC, Short TG. Total intravenous anaesthesia using propofol infusion — 50 consecutive cases. Anaesth Intensive Care 1988; 16: 150–7

33.	Gunawardene RD. Propofol and dreaming. Anaesthesia 1989; 44: 266–7

34.	Restall J, Johnston IG, Robinson DN. Flumazenil in ketamine and midazolam anaesthesia. Anaesthesia 1990; 45: 938–40

35.	Liu WHD, Thorp TAS, Graham SG, Aitkenhead AR. Incidence of awareness with recall during general anaesthesia. Anaesthesia 1991; 46: 435–7

36.	Lyons G, Macdonald R. Awareness during Caesarean section. Anaesthesia 1991; 46: 62–4

37.	Marsch SC, Schaefer HG, Tschan C, Meier B. Dreaming and anaesthesia: total i.v. anaesthesia with propofol versus balanced volatile anaesthesia with enflurane. Eur J Anaesthesiol 1992; 9: 331–3

38.	Jakobsson J, Oddby E, Rane K. Patient evaluation of four different combinations of intravenous anaesthetics for short outpatient procedures. Anaesthesia 1993; 48: 1005–7

39.	Oddby‐Muhrbeck E, Jakobsson J. Recall of music: a comparison between anaesthesia with propofol and isoflurane. Acta Anaesthesiol Scand 1993; 37: 33–7

40.	Krissel J, Dick WF, Leyser KH, Gervais H, Brockerhoff P, Schranz D. Thiopentone, thiopentone/ketamine, and ketamine for induction of anaesthesia in caesarean section. Eur J Anaesthesiol 1994; 11: 115–22

41.	Oxorn D, Orser B, Ferris LE, Harrington E. Propofol and thiopental anesthesia: A comparison of the incidence of dreams and perioperative mood alterations. Anesth Analg 1994; 79: 553–7

42.	Brandner B, Blagrove M, McCallum G, Bromley LM. Dreams, images and emotions associated with propofol anaesthesia. Anaesthesia 1997; 52: 750–5

43.	Kee WDN, Khaw KS, Ma ML, Mainland P-A, Gin T. Postoperative analgesic requirement after cesarean section: A comparison of anesthetic induction with ketamine or thiopental. Anesth Analg 1997; 85: 1294

44.	Nordström O, Engström AM, Persson S, Sandin R. Incidence of awareness in total i.v. anaesthesia based on propofol, alfentanil and neuromuscular blockade. Acta Anaesthesiol Scand 1997; 41: 978–84

45.	Oxorn DC, Ferris LE, Harrington E, Orser BA. The effects of midazolam on propofol-induced anesthesia: propofol dose requirements, mood profiles, and perioperative dreams. Anesth Analg 1997; 85: 553–9

46.	Ranta SO, Laurila R, Saario J, Ali-Melkkilä T, Hynynen M. Awareness with recall during general anesthesia: incidence and risk factors. Anesth Analg 1998; 86: 1084–9

47.	Chudnofsky CR, Weber JE, Stoyanoff PJ, et al. A combination of midazolam and ketamine for procedural sedation and analgesia in adult emergency department patients. Acad Emerg Med 2000; 7: 228–35

48.	Ghoneim MM, Block RI, Dhanaraj VJ, Todd MM, Choi WW, Brown CK. Auditory evoked responses and learning and awareness during general anesthesia. Acta Anaesthesiol Scand 2000; 44: 133–43

49.	Grace RF, Lesteour T, Sala T, Stewart J. A randomized comparison of low-dose ketamine and lignocaine infiltration with ketamine-diazepam anaesthesia for post-partum tubal ligation in Vanuatu. Anaesth Intensive Care 2001; 29: 30–3

50.	Aceto P, Valente A, Adducci E, Gorgoglione M, De Cosmo G. Implicit memory, dream and auditory evoked responses during anaesthesia. Acta Medica Romana 2002; 40: 21–9

51.	Vallejo MC, Romeo RC, Davis DJ, Ramanathan S. Propofol-ketamine versus propofol-fentanyl for outpatient laparoscopy. J Clin Anesth 2002; 14: 426–31

52.	Aceto P, Valente A, Gorgoglione M, Adducci E, De Cosmo G. Relationship between awareness and middle latency auditory evoked responses during surgical anaesthesia. Br J Anaesth 2003; 90: 630–5

53.	Grace RF. The effect of variable-dose diazepam on dreaming and emergence phenomena in 400 cases of ketamine-fentanyl anaesthesia. Anaesthesia 2003; 58: 904–10

54.	Hellwagner K, Holzer A, Gustorff B, et al. Recollection of dreams after short general anaesthesia: influence on patient anxiety and satisfaction. Eur J Anaesthesiol 2003; 20: 282–8

55.	Iwama H, Obara S, Ozawa S, et al. A survey of combined epidural-propofol anesthesia with noninvasive positive pressure ventilation as a minimally invasive anesthetic protocol. Med Sci Monit 2003; 9: CR316–23

56.	Kudoh A, Takase H, Takazawa T. A comparison of anesthetic quality in propofol-spinal anesthesia and propofol-fentanyl anesthesia for total knee arthroplasty in elderly patients. J Clin Anesth 2004; 16: 405–10

57.	Kwok RFK, Lim J, Chan MTV, Gin T, Chiu WKY. Preoperative ketamine improves postoperative analgesia after gynecologic laparoscopic surgery. Anesth Analg 2004; 1044–9

58.	Myles PS, Leslie K, McNeil J, Forbes A, Chan MTV. Bispectral index monitoring to prevent awareness during anaesthesia: the B-Aware randomised controlled trial. The Lancet 2004; 363: 1757–63

59.	Wanna O, Werawatganon T, Piriyakitphaiboon S, Taesiri B. A comparison of propofol and ketamine as induction agents for cesarean section. J Med Assoc Thai 2004; 87: 774–9

60.	Leslie K, Myles PS, Forbes A, Chan MTV, Swallow SK, Short TG. Dreaming during anaesthesia in patients at high risk of awareness. Anaesthesia 2005; 60: 239–44

61.	Aceto P, Congedo E, Lai C, Valente A, Gualtieri E, De Cosmo G. Dreams recall and auditory evoked potentials during propofol anaesthesia. NeuroReport 2007; 18: 823–6

62.	Leslie K, Skrzypek H, Paech MJ, Kurowski I, Whybrow T. Dreaming during anesthesia and anesthetic depth in elective surgery patients: a prospective cohort study. Anesthesiology 2007; 106: 33–42

63.	Toscano A, Pancaro C, Peduto VA. Scopolamine prevents dreams during general anesthesia. Anesthesiology 2007; 106: 952–5

64.	Errando CL, Sigl JC, Robles M, et al. Awareness with recall during general anaesthesia: a prospective observational evaluation of 4001 patients. Br J Anaesth 2008; 101: 178–85

65.	Samuelsson P, Brudin L, Sandin RH. BIS does not predict dreams reported after anaesthesia. Acta Anaesthesiol Scand 2008; 52: 810–4

66.	Samuelsson P, Brudin L, Sandin RH. Intraoperative dreams reported after general anaesthesia are not early interpretations of delayed awareness. Acta Anaesthesiol Scand 2008; 52: 805–9

67.	Stait ML, Leslie K, Bailey R. Dreaming and recall during sedation for colonoscopy. Anaesth Intensive Care 2008; 36: 685–90

68.	Eer AS, Padmanabhan U, Leslie K. Propofol dose and incidence of dreaming during sedation. Eur J Anaesth 2009; 26: 833–6

69.	Leslie K, Sleigh J, Paech MJ, Voss L, Lim CW, Sleigh C. Dreaming and electroencephalographic changes during anesthesia maintained with propofol or desflurane. Anesthesiology 2009; 111: 547–55

70.	Paech MJ, Scott KL, Clavisi O, Chua S, McDonnell N. A prospective study of awareness and recall associated with general anaesthesia for caesarean section. Int J Obstet Anesth 2009; 17: 298–303

71.	Cheong SH, Lee KM, Lim SH, et al. The effect of suggestion on unpleasant dreams induced by ketamine administration. Anesth Analg 2011; 112: 1082

72.	Kim D-K, Joo Y, Sung T-Y, Kim S-Y, Shin H-Y. Dreaming in sedation during spinal anesthesia: a comparison of propofol and midazolam infusion. Anesth Analg 2011; 112: 1076–81

73.	Xu G, Liu X, Chen J, Zhang J, Wang K. First-trimester surgical abortion increasing the incidence of dreaming: Influence on patient satisfaction during propofol short sedation. Neurosurg Q 2012; 22: 261

74.	Xu GH, Liu XS, Yu FQ, Gu EW, Zhang J, Wang K. Dreaming during sevoflurane or propofol short-term sedation: a randomised controlled trial. Anaesth Intensive Care 2012; 40: 505–10

75.	Aceto P, Lai C, Perilli V, et al. Stress-related biomarkers of dream recall and implicit memory under anaesthesia. Anaesthesia 2013; 68: 1141–7

76.	Czarko K, Kwiatosz-Muc M, Fijałkowska A, Kowalczyk M, Rutyna R. Intraoperative awareness--comparison of its incidence in women undergoing general anaesthesia for caesarean section and for gynaecological procedures. Anaesthesiol Intensive Ther 2013; 45: 200–4

77.	Russell IF. The ability of bispectral index to detect intra‐operative wakefulness during isoflurane/air anaesthesia, compared with the isolated forearm technique. Anaesthesia 2013; 68: 1010–20

78.	Xu G, Liu X, Sheng Q, Yu F, Wang K. Sex differences in dreaming during short propofol sedation for upper gastrointestinal endoscopy. NeuroReport 2013; 24: 797–802

79.	Tezcan AH, Ornek DH, Ozlu O, et al. Abuse potential assessment of propofol by its subjective effects after sedation. Pak J Med Sci 2014; 30: 1247–52

80.	Aceto P, Perilli V, Lai C, et al. Minimum alveolar concentration threshold of sevoflurane for postoperative dream recall. Minerva Anestesiol 2015; 81: 1201–9

81.	Gyulaházi J, Varga K, Iglói E, Redl P, Kormos J, Fülesdi B. The effect of preoperative suggestions on perioperative dreams and dream recalls after administration of different general anesthetic combinations: a randomized trial in maxillofacial surgery. BMC Anesthesiol 2015; 15: 11

82.	Gyulaházi J, Redl P, Karányi Z, Varga K, Fülesdi B. Dreaming under anesthesia: is it a real possiblity? Investigation of the effect of preoperative imagination on the quality of postoperative dream recalls. BMC Anesthesiology 2016; 16: 53

83.	Cascella M, Fusco R, Caliendo D, et al. Anesthetic dreaming, anesthesia awareness and patient satisfaction after deep sedation with propofol target controlled infusion: A prospective cohort study of patients undergoing day case breast surgery. Oncotarget 2017; 8: 79248–56

84.	Villegas S, Suarez S, Owuor J, et al. Intraoperative awareness and experience with a ketamine-based anaesthesia package to support emergency and essential surgery when no anaesthetist is available. Afr J Emerg Med 2019; 9: S56–60

85.	Chen L, Zhang J, He W, Liu W. Comparative effects of dexmedetomidine and midazolam on dreaming of patients undergoing flexible bronchoscopy during general anesthesia. Med Sci Monit 2020; 27. Available from: https://www.medscimonit.com/abstract/index/idArt/929000

86.	Hao L, Hu X, Zhu B, Li W, Huang X, Kang F. Clinical observation of the combined use of propofol and etomidate in painless gastroscopy. Medicine 2020; 99: e23061

87.	Yoshida A, Fujii K, Yoshikawa T, Kawamata T. Factors associated with quality of dreams during general anesthesia: a prospective observational study. J Anesth 2021; 35: 576–80

88.	Alam T, Qureshi R, Aziz M, Khan A. Incidence of accidental awareness during general anesthesia — a tertiary cancer care hospital experience. J Postgrad Med Inst 2022; 36: 3–8

89.	Matus H, Kvolik S, Rakipovic A, Borzan V. Bispectral index monitoring and observer rating scale correlate with dreaming during propofol anesthesia for gastrointestinal endoscopies. Medicina 2022; 58: 62

90.	Schäfer B, Klose P, Wiltfang J, Fudickar A. Incidence of dreaming and subjective quality of general anesthesia with and without standardized stress relaxation and dream suggestion by the anesthesiologist: A prospective interventional randomized trial using an adaptive design. Dreaming 2023; 33: 264–74

91.	McKie DBD, Thorp EA. Awareness and dreaming during anaesthesia in a paediatric hospital. Anaesth Intensive Care 1973; 1: 404–14

92.	Feingold A, MacMahon S. Patient preference for ketamine: A case report of multiple anesthesias. Anesth Analg 1975; 54: 35–7

93.	Elliott E, Hanid TK, Arthur LJ, Kay B. Ketamine anaesthesia for medical procedures in children. Arch Dis Child 1976; 51: 56–9

94.	Krantz EM. Low-dose intramuscular ketamine and hyaluronidase for induction of anaesthesia in non-premedicated children. S Afr Med J 1980; 58: 161–2

95.	Tamminga RYJ, Noordhoek M, Kroon J, Faber-Nijholt R. Ketamine anesthesia with or without diazepam premedication for bone marrow punctures in children with acute lymphoblastic leukemia. Pediatr Hematol Oncol 2000; 17: 383–8

96.	Wathen JE, Roback MG, Mackenzie T, Bothner JP. Does midazolam alter the clinical effects of intravenous ketamine sedation in children? A double-blind, randomized, controlled, emergency department trial. Ann Emerg Med; 2000; 36: 579–88

97.	Huang GH, Davidson AJ, Stargatt R. Dreaming during anaesthesia in children: incidence, nature and associations. Anaesthesia 2005; 60: 854–61

98.	Da Silva PSL, De Aguiar VE, Waisberg DR, Passos RMA, Park MVF. Use of Ketofol for procedural sedation and analgesia in children with hematological diseases. Pediatr Int 2011; 53: 62–7

99.	Wilson RD, Allen CR, Creson D, Schmitt R, Barratt E, Traber DL. Operant behavior and neurophysiological observations of normal human volunteers receiving CL-1848C, a new dissociative anesthetic. Tex Rep Biol Med 1973; 31: 239–48

100.	Morse Z, Sano K, Kanri T. Effects of a midazolam-ketamine admixture in human volunteers. Anesth Prog 2004; 51: 76–9

101.	Noreika V, Jylhänkangas L, Móró L, et al. Consciousness lost and found: subjective experiences in an unresponsive state. Brain Cogn 2011; 77: 327–34

102.	Sarasso S, Boly M, Napolitani M, et al. Consciousness and complexity during unresponsiveness induced by propofol, xenon, and ketamine. Curr Biol 2015; 25: 3099–105

103.	Bonhomme V, Vanhaudenhuyse A, Demertzi A, et al. Resting-state network-specific breakdown of functional connectivity during ketamine alteration of consciousness in volunteers. Anesthesiology 2016; 125: 873–88

104.	Radek L, Kallionpää RE, Karvonen M, et al. Dreaming and awareness during dexmedetomidine- and propofol-induced unresponsiveness. Br J Anaesth 2018; 121: 260–9

105.	Radek L, Koskinen L, Sandman N, et al. On no man’s land: Subjective experiences during unresponsive and responsive sedative states induced by four different anesthetic agents. Conscious Cogn 2021; 96: 103239

106.	Valli K, Radek L, Kallionpää RE, et al. Subjective experiences during dexmedetomidine- or propofol-induced unresponsiveness and non-rapid eye movement sleep in healthy male subjects. Br J Anaesth; 2023; 131: 348–59
